# *o*-Halogenation and -Alkoxylation of Phenylglycine Derivatives by Pd-Mediated C-H Functionalization: Scope and Limitations

**DOI:** 10.3390/molecules30020236

**Published:** 2025-01-09

**Authors:** Eduardo Laga, Sonia Nieto, Carlos Cativiela, Esteban P. Urriolabeitia

**Affiliations:** Instituto de Síntesis Química y Catálisis Homogénea, ISQCH (CSIC—Universidad de Zaragoza), Pedro Cerbuna 12, 50009 Zaragoza, Spain; edulala@gmail.com (E.L.); snietoap@gmail.com (S.N.); cativiela@unizar.es (C.C.)

**Keywords:** amino acids, palladium, C-H bond activation, halogenation, alkoxylation

## Abstract

Orthopalladated derivatives from substituted phenylglycines [Pd(μ-Cl)(C_6_H_3_R_1_C(R_2_)(R_3_)N(R_4_)_2_]_2_ (**1**) react with halogenating reagents (PhICl_2_, Br_2_, I_2_) (**2**) to give the corresponding o-halogenated amino acids C_6_H_3_(X)R_1_C(R_2_)(R_3_)N(R_4_)_2_ (**3**). The reaction is general and tolerates a variety of functional groups (R_1_ to R_4_) at the aryl ring, the Cα, and the N atom. On the other hand, the reaction of [Pd(μ-Cl)(C_6_H_3_R_1_C(R_2_)(R_3_)N(R_4_)_2_]_2_ (**1**) with PhI(OAc)_2_ in the presence of a variety of alcohols R_5_OH (**4**) gives the o-alkoxylated phenylglycines C_6_H_3_(OR_5_)R_1_C(R_2_)(R_3_)N(R_4_)_2_ (**5**), also as a general process. A partial loss of the enantiomeric excess is observed when the starting phenylglycine is enantiomerically pure, this arising from the formation of bridging azavinylidene (**6**) and imine intermediate species (**7**), which were characterized by X-ray diffraction methods.

## 1. Introduction

The methodology of functionalizing organic molecules through C-H bond activation promoted by transition metals has established itself as one of the most powerful and versatile synthesis tools available to preparative chemists [1,2,3,4,5,6,7,8,9,10,11,12]. This approach enables the transformation of virtually any type of C-H bond (Csp^3^, Csp^2^, or Csp) into C-C, C-N, C-O, C-S, or C-hal bonds, among the most important instances, using a plethora of transition metals, including first-row metals. The breadth of the scope of organic synthesis through organometallics not only encompasses the types of bonds to be activated and metals but also substrates, including very fragile and/or reactive molecules. In recent years, new methodologies for C-H bond functionalization have also been successfully added to the standard toolkit. For instance, photochemistry [13,14,15,16,17], electrochemistry [17,18,19,20,21], and mechanochemistry [22,23,24,25,26], among others, have proven to be particularly efficient in this field.

α-Amino acids, fundamental chiral molecules that constitute proteins and are essential for life, belong to a category of reactive molecules whose functionalization is of great interest. The sequence of amino acids in a protein determines how it folds into a three-dimensional structure, which, in turn, defines the protein’s function. A change in the amino acid sequence or in the structure of any of them can lead to a change in folding and, consequently, a change in the activity of the protein to which they belong [27]. This fact opens the possibility of the targeted functionalization of amino acids to obtain proteins with properties tailored to specific needs. For this reason, the synthesis and modification of amino acids is of great interest in the field of biomedical research.

C-H functionalization is a suitable tool for the modification of amino acids [28,29,30,31,32,33,34,35,36,37,38,39,40,41,42,43]. However, it is not very common to carry out C-H activation processes directly on amino acids themselves, due to the large number of functional groups they contain and their incompatibility in certain cases with reaction conditions. In amino acids, both the amino group and the acid group can interact with the metal and coordinate to it. Since there are two coordinating groups, the usual reactivity of amino acids with metals involves the formation of N,O-chelates. When this occurs, the metal often loses its ability to perform C-H bond activation, as the positions containing the C-H bonds are far from the coordination sphere of the metal. For this reason, to carry out C-H activation processes in amino acids, it is usually necessary to modify them to reduce their chelating capacity. Generally, it is sufficient to protect the acid group as an ester while leaving the amino group free as a directing group to achieve appropriate reactivity and selectivity.

**Figure 1:**
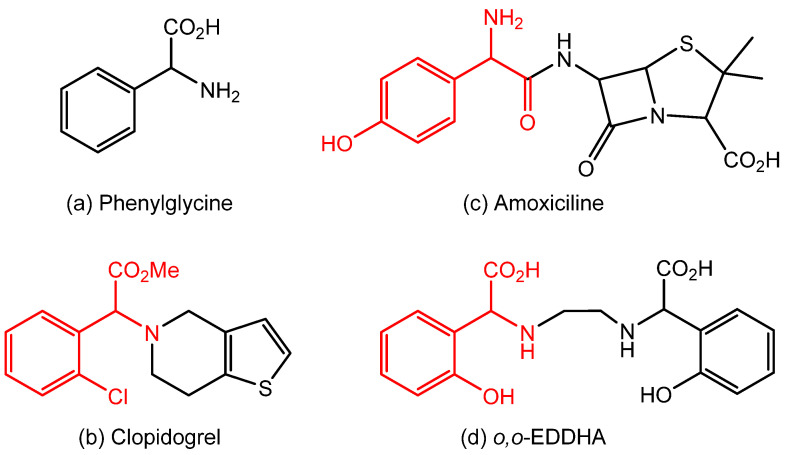
The amino acid phenylglycine (PhG) and some derivatives of interest that contain it.

Phenylglycine (Figure 1a) can be considered the simplest α-amino acid in the aromatic series. Although it is not one of the proteinogenic amino acids, it exhibits significant biological activity [44,45,46,47,48]. In fact, functionalized phenylglycines are components of several products listed among the top 200 pharmaceutical products by retail sales in the USA, such as clopidogrel (Figure 1b) and amoxicillin (Figure 1c) [49,50], and they are also present in the basic structure of vancomycin, teicoplanin, and cefoperazone. These are drugs that act as antiplatelets, antibiotics, and bacteriostatic agents. Furthermore, other alkoxylated derivatives, such as those shown in Figure 1d, serve as precursors in the synthesis of products with agrochemical applications [51,52]. For example, *o*,*o*-EDDHA is one of the most widely used chelating agents for iron sequestration and for treating iron chlorosis in plants [53].

Although the uses of phenylglycine are of such an important extension, the classical synthesis of this molecule is restricted to a scarce number of strategies. In addition, tedious procedures and/or low conversions are usually involved [54]. Due to the high value and interest of the final species, the development of alternative procedures, such as CH functionalization, is desirable. However, very few examples are known where phenylglycine is functionalized through C-H activation promoted by transition metals [55,56,57,58]. In general, the functionalization of amino acids requires not only the protection of the acid group as an ester, as mentioned earlier, but also the protection of the amino group with standard protecting agents. Furthermore, in a significant portion of the reported studies [59,60,61,62], it is not only necessary to protect the N group but also to install a chelating organic auxiliary group to assist C-H activation and subsequent functionalization. This auxiliary group must then be removed after functionalization. All protections and deprotections add extra synthetic steps to the overall process, resulting in longer, more expensive, and more energy-demanding processes, which are ultimately less sustainable. The ideal scenario would involve minimizing the number of protecting groups and ensuring their installation and removal are as simple as possible. For all these reasons combined, the straightforward functionalization of phenylglycine remains a challenge.

The contributions made by our research group in this area demonstrate that it is possible to regioselectively functionalize phenylglycine at the ortho position of the phenyl ring by protecting only the ester group and leaving the amino group free, both stoichiometrically [63,64,65,66] and catalytically [67]. An additional aspect to consider concerns the enantioselectivity of the functionalization processes when the starting phenylglycine is enantiomerically pure. While the incorporation of Pd into the molecular framework occurs with complete retention of configuration [63,68], and the resulting orthopalladated intermediate is also enantiomerically pure, the subsequent functionalization proceeds with some loss of chirality [63]. This phenomenon has been described in similar cases [58] and is related to the ease of racemization and degradation of phenylglycine itself [69].

The work presented here involves the halogenation and alkoxylation of phenylglycines substituted both on the aryl ring and at the Cα and N positions. It also includes a study of how the chirality of the precursor molecule is degraded, identifying unprecedented intermediate species that are responsible for this degradation, and characterizing several intermediates relevant to the loss of chirality.

## 2. Results and Discussion

### 2.1. Halogenation Reactions of Phenylglycine

**Figure 2:**
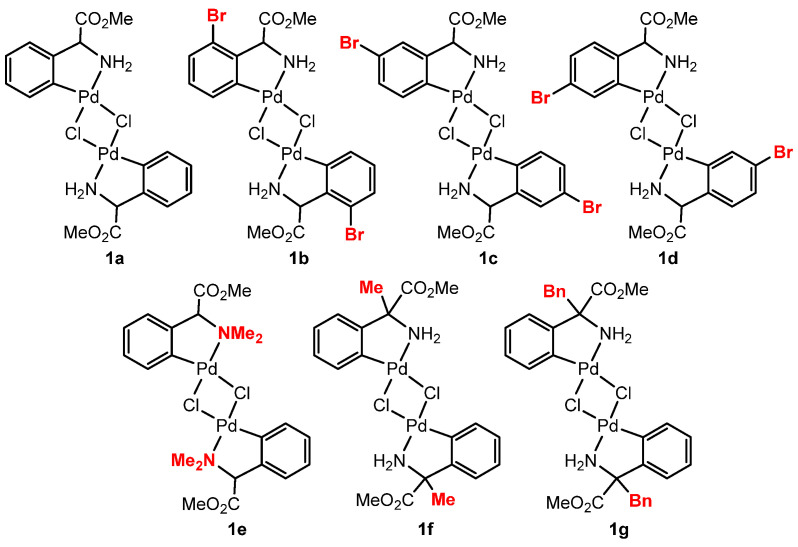
Pd precursors that have been tested in the halogenation reaction.

For the study of halogenation reactions, the dinuclear palladium precursors shown in Figure 2 have been used. These complexes feature a variety of substituents, aimed at covering different structural possibilities to analyze the influence of all potential factors that could modulate reactivity. Thus, complexes without substituents (**1a**) will be used as a reference, along with those where the phenyl ring is substituted with a Br atom in positions 2 (**1b**), 3 (**1c**), and 4 (**1d**); with two substituents on the amine nitrogen (NMe_2_) (**1e**); and with methyl (**1f**) and benzyl (**1g**) substituents on the Cα. All these complexes have been prepared through C-H activation from the corresponding phenylglycines, following methods described in the literature [65,68]. Since only complex (**1a**) was obtained enantiomerically pure and all the other derivatives (**1b**)–(**1g**) were obtained as racemic mixtures, the behavior of the enantiomeric excess was not considered here.

**Scheme 1:**
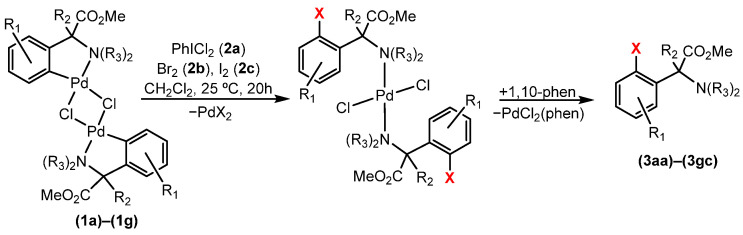
Two-step synthesis of ortho-halogenated phenylglycines (**3aa**)–(**3gc**).

**Figure 3:**
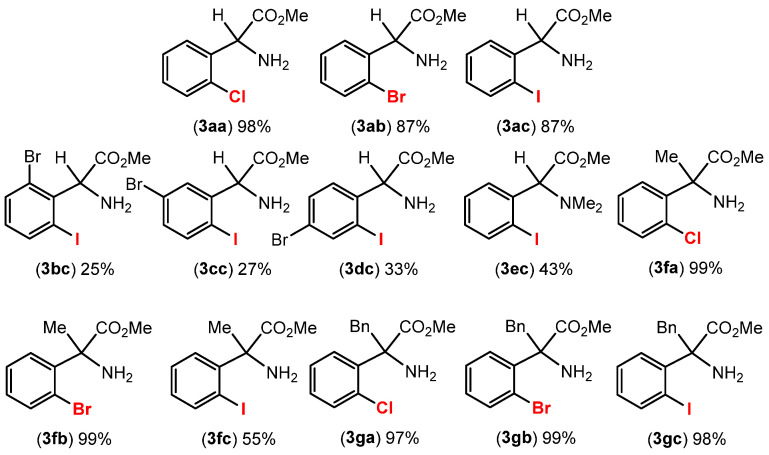
Scope of the halogenation reaction.

The method used to carry out the halogenation reactions involves treating the orthopalladates (**1a**)–(**1g**) with stoichiometric amounts of PhICl_2_ (**2a**) [70], Br_2_ (**2b**), or I_2_ (**2c**) in CH_2_Cl_2_ at room temperature for 20 h. Neither molecular Cl_2_ (dissolved in CCl_4_) nor other typical chlorinating agents, such as *N*-chlorosuccinimide, enabled the synthesis of chlorinated phenylglycines. As a result of the oxidative coupling process, an equimolar mixture of PdX_2_ (X = Cl, Br, I), which precipitates in the reaction medium, and a mononuclear complex with the stoichiometry PdCl_2_L_2_, where L is the ortho-halogenated phenylglycine, is obtained, as shown in Scheme 1. After removing the PdX_2_ by filtration, adding 1,10-phenanthroline to the PdCl_2_L_2_ complex displaces the halogenated phenylglycine ligand, leading to the synthesis of the corresponding chlorinated, brominated, and iodinated compounds. The reaction scope is shown in Figure 3.

The derivatives (**3aa**)–(**3ac**) have already been reported but are included for comparison purposes and because their synthesis has been improved in terms of yield compared to the methods described in the literature [63,71]. The derivatives (**3bc**), (**3dc**), (**3ec**), (**3ga**), (**3gb**), and (**3gc**) have not been previously described, while the derivatives (**3cc**), (**3fa**), (**3fb**), and (**3fc**) appear in SciFinder but lack any associated references. Therefore, they have been fully characterized in this work.

As shown in Figure 3, the halogenation of (**1a**) proceeds with very good yields for all three substrates, consistently generating the monosubstituted derivative with complete regioselectivity at the ortho position of the phenyl ring. In the case of the reactivity of (**1b**), (**1c**), and (**1d**)—complexes that feature a bromine atom on the cyclopalladated ring at different positions—clear results were only obtained in the iodination reactions. Despite observing a clear reaction, it was not possible to isolate pure compounds in the chlorination and bromination reactions. The dihalogenated derivatives (**3bc**), (**3cc**), and (**3dc**) are obtained with moderate to low yields. The yields are similar in all three cases, suggesting that the position of the bromine atom on the ring does not influence the reaction. However, they are noticeably lower than the yield observed for the unsubstituted derivative (**3ac**), indicating a negative effect of the bromine atom’s presence.

A possible explanation for this low yield is that the presence of an electron-withdrawing group on the phenyl ring reduces its electron density, making halogenation less favorable regardless of the mechanism by which halogenation occurs, whether via oxidative addition or electrophilic aromatic substitution [72,73,74,75]. The characterization of (**3bc**), (**3cc**), and (**3dc**) was based on their NMR data. In addition to the expected signals for the CαH, NH_2_, and CO_2_Me groups, the spin systems of the aromatic group in each compound are diagnostic. The synthetic interest in these types of dihalogenated derivatives with two distinct groups (one iodine and one bromine) is very high, as they enable subsequent orthogonal functionalizations that differentiate between the two halogens, allowing for the incorporation of two distinct groups at predefined and distinct positions on the aromatic ring [76].

Regarding the influence of the nitrogen atom substituents, the presence of the NMe_2_ group also has a somewhat negative effect on halogenation, as the derivative (**3ec**) is obtained with moderate yield. The NMe_2_ group is electron-donating, which should favor the oxidative addition reaction. However, it also increases steric hindrance. During the oxidative addition step, changes occur in the metal’s coordination sphere. If the metal center is sterically hindered, the destabilization of the oxidized intermediate is greater, making the process less favorable. Based on the results obtained for the synthesis of compound (**3ec**), it appears that adverse steric effects outweigh electronic effects and are the primary factors controlling the reaction. On the other hand, the presence of substituents on the Cα of phenylglycine has a clear positive effect on halogenation, as the derivatives (**3fa**)–(**3gc**) are obtained with very good yields in all cases. The increase in electron density caused by the methyl and benzyl groups on the Cα and the significant improvement in yield are consistent with both an oxidative addition mechanism (more likely in chlorination and bromination) and an electrophilic substitution mechanism (S_E_Ar, more probable in the case of iodination) [77].

In summary, the reactivity of Pd complexes (**1a**)–(**1g**) with simple halogenating agents such as PhICl_2_, Br_2_, or I_2_ allows the synthesis of ortho-monohalogenated phenylglycines across a wide range of substrates, generally achieving moderate to very good yields.

### 2.2. Alkoxylation Reactions of Phenylglycine

**Scheme 2:**
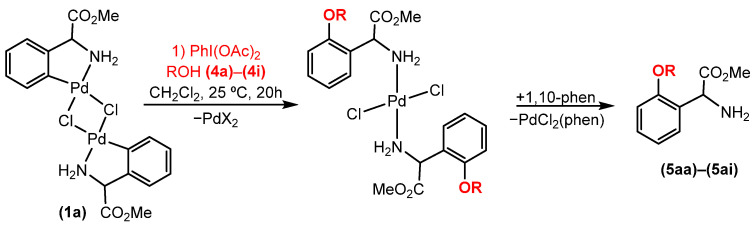
Two-step synthesis of ortho-alkoxylated phenylglycines (**5aa**)–(**5ai**).

The methodology used for the alkoxylation of phenylglycines was similar to that described for halogenation, with the logical difference in the nature of the substrates and the introduction of an additional step to optimize the outcome. Therefore, the dinuclear derivative (**1a**) is reacted with the oxidant PhI(OAc)_2_ using an alcohol as the solvent at room temperature for 20 h. After the reaction, the alcohol is evaporated to complete dryness, and the residue is redissolved in CH_2_Cl_2_. An insoluble fraction remains, which is discarded as it does not contain phenylglycine, and a soluble fraction containing the species with the stoichiometry PdCl_2_L_2_ is obtained, where L represents the alkoxylated phenylglycine as shown in Scheme 2. This species is purified by flash column chromatography, a key step in obtaining products with a high enantiomeric excess (ee, %). The isolation of the PdCl_2_L_2_ complex and its subsequent treatment with 1,10-phenanthroline allows for the removal of palladium as the insoluble species PdCl_2_(phen) and the release of the corresponding ortho-alkoxylated phenylglycines (**5**) in pure form, as described in the experimental section.

This methodology had previously been tested using methanol and ethanol as solvents, yielding the corresponding ortho-alkoxylated phenylglycines with yields around 60%. However, there was a significant loss of enantiomeric excess (ee = 22% and 50%, respectively, in the final products) [63]. The introduction of the additional purification step for the PdCl_2_L_2_ species allows for the methoxylated and ethoxylated derivatives to be obtained with ee values of 96%, representing a significant improvement over the previous method. Therefore, this synthetic methodology for the alkoxylation of phenylglycines described here will be employed. Unlike the previous section, this part uses the chiral complex (*R*,*R*)-(**1a**) [63,65,68], and the evolution of chirality throughout the reaction will be analyzed. In all cases, enantiomeric excesses (ee) were determined via chiral derivatization using Mosher’s acid chloride [*R*-(-)-*R*-methoxy-*R*-trifluoromethylphenylacetyl chloride, (*R*)-MTPACl] and recording the ^19^F spectra of the diastereomer mixture [78]. Other techniques described in the literature did not yield satisfactory results, either due to racemization occurring during derivatization or due to inadequate signal separation for the sample to be analyzed by NMR.

**Figure 4:**
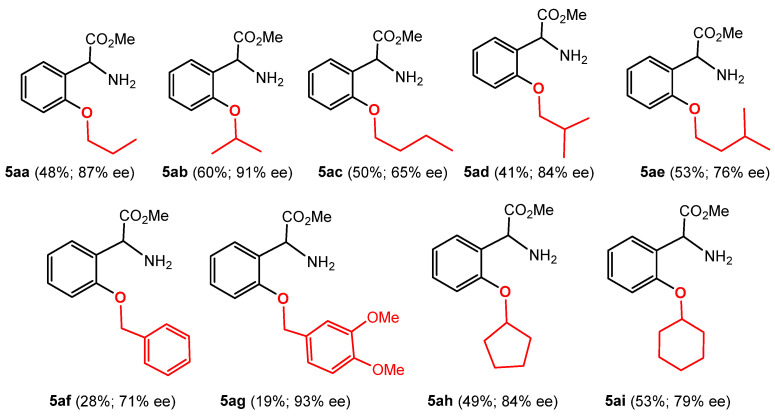
Pd-mediated ortho-alkoxylation of phenylglycines: reaction scope.

Additional attempts to optimize this process have been carried out by modifying (i) the orthopalladated complex, (ii) the oxidant, and (iii) the temperature (see Supplementary Material, Table S1). Regarding the Pd precursor complex, the dinuclear complex with chloride bridges (**1a**) has delivered the best results, both in terms of yield and enantiomeric excess, as the use of other dinuclear Pd complexes (**1b**, **1c**) or mononuclear ones (**1d**) consistently resulted in lower ee and yields, with instances where no reaction was observed at all. In the case of oxidant screening, PhI(OAc)_2_ provided the best results, as no other tested oxidant allowed for the alkoxylated phenylglycine to be obtained. Temperature modification has a notably negative influence on the progress of the reaction. For instance, when the reaction is performed at 100 °C, there is a sharp drop in yield and a complete loss of chirality, resulting in a low yield of a racemic product. Once the reaction conditions were optimized, the method was extended to different alcohols. The scope of the reaction is shown in Figure 4.

As shown in Figure 4, the reaction is general and works efficiently for primary and secondary alcohols, whether they have a linear or branched alkyl chain, a benzyl group, or a cycloalkane. The yields in all cases range from moderate (40–60%) to low (20–30%). On the other hand, the measured ee values are above 65% in all cases, reaching over 90% in some examples, indicating good preservation of chirality under the optimized conditions. However, this also implies that a certain degree of the original chiral information is lost during the reaction, a fact that must be further analyzed.

The characterization of phenylglycines (**5aa**)–(**5ai**) was performed based on their analytical and spectroscopic data (see Experimental). The presence of four peaks in the aromatic region of the ^1^H NMR spectra, two with hyperfine doublet structures and two as triplets, confirms the presence of a C_6_H_4_ fragment, 1,2-disubstituted. Signals corresponding to the NH_2_ and CO_2_Me groups, and those due to the incorporated alcohol group, are also observed with the expected integration. In the ^13^C (APT) NMR spectrum, the C_6_H_4_ ring is characterized by four signals assigned to C-H-type carbons and two out-of-phase signals corresponding to quaternary carbons. One of the quaternary carbon signals appears notably deshielded, around 150–160 ppm, and is assigned to the C-O carbon, confirming the alkoxylation.

**Figure 5:**
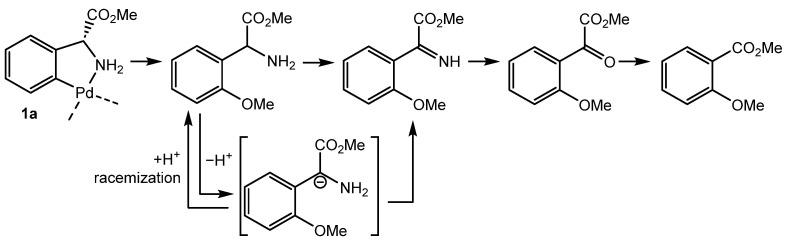
Proposed degradation of the methyl ester of phenylglycine.

As shown in Figure 4, the alkoxylation reaction at room temperature produces moderate yields of phenylglycines (**5aa**)–(**5ai**). When analyzing the crude reaction mixture by NMR before chromatographic purification, in addition to species containing phenylglycine, signals attributable to the presence of imines and ketoesters, products of phenylglycine degradation, are observed. In certain cases, signals assigned to the ester of benzoic acid, the final product of this degradation, are also detected. This suggests that the moderate-to-low yield may be due to the partial degradation of phenylglycine-containing species, which occurs even at room temperature. This degradation is directly related to the acidity of the H on the Cα, as the deprotonation of Cα is the first step in the formation of the imine. Furthermore, this deprotonation is logically linked to the loss of enantiomeric excess, as the formation of the carbanion inherently leads to the formation of a planar species, and subsequent reprotonation can occur from either side, resulting in racemization. The possible processes are depicted in Figure 5. As noted in the literature, the loss of ee in this type of compound is almost unavoidable [58]. When the temperature increases, both the deprotonation and subsequent imine formation are favored, leading to higher percentages of imine and ketoacid and a reduction in phenylglycine content. This explains the greater decrease in yield and the complete loss of chiral information at elevated temperatures.

**Figure 6:**
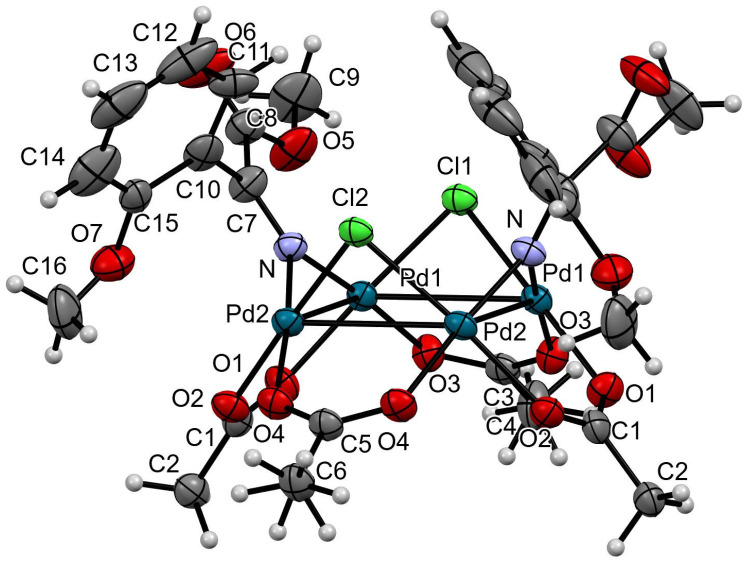
Structure of the derivative **6**. Ellipsoids of non-hydrogen atoms have been drawn at 50% probability.

Given that the formation of imine species is important for understanding how the reaction works, we have focused our efforts on demonstrating their presence. The formation of organometallic complexes has made it possible to isolate primary imines coordinated to palladium [79,80], and, moreover, the formation of imines requires oxidizing conditions [81]. For this reason, the reaction of compound (**1a**) in methanol was carried out with higher amounts of PhI(OAc)_2_ as the oxidizing agent (10 equivalents) for 6 h. Under these reaction conditions, in addition to the PdCl_2_L_2_ species, it was possible to isolate and crystallize complex **6**, which is air-stable. Its structural elucidation by X-ray diffraction revealed the connectivity shown in Figure 6. The key aspect of this structure is that it demonstrates the presence of an azavinylidene ligand bridging two palladium atoms. This ligand originates from a methyl phenylglycinate ligand in which the CH-NH_2_ group has been oxidized to a [C=N]^−^ group, meaning an α-iminoester anionic species coordinated to palladium is observed (see Figure 6).

Compound **6** crystallizes in the orthorhombic space group *P*nma. The metal atoms form a square arrangement of four palladium atoms (Pd_4_), with slight deviations from the ideal square geometry, supported by the presence of two bridging ligands on each side. One of the symmetry planes of the space group bisects the Pd1-Pd1* and Pd2-Pd2* sides and contains the two Cl1 and Cl2 atoms, running parallel to the Pd1-Pd2 axis. The slightly longer sides, Pd1-Pd1* and Pd2-Pd2* [3.1945(15) and 3.1546(15) Å], are those supported by an acetate ligand and a chloride ligand as bridges. In contrast, on the other two sides, Pd1-Pd2 [3.0951(10) Å], the palladium atoms are bridged by an acetate ligand and an azavinylidene bridging ligand. The four acetate ligands adopt a cisoid arrangement with respect to the plane formed by the palladium atoms. The distances between the palladium atoms are all greater than the Pd-Pd bond distance (2.7511 Å) [82], suggesting that there is no bonding interaction between them.

The coordination environment of the metal centers in all cases has a square-planar geometry, with the Pd1 atom deviating by 0.0120(7) Å from the least-squares plane defined by the atoms coordinated to it. The Cl bridge is symmetric, with a Pd1-Cl2-Pd1* angle close to perpendicularity [84.59(10)° and 87.17(11)°]. The Pd1-Cl2 and Pd2-Cl1 bond distances are 2.344(2) Å and 2.317(2) Å, respectively, falling within the range reported for cases where the Pd-Cl-Pd angles are less than 85° [2.390(51) Å] [83]. The azavinylidene ligands derived from the amino acid adopt a cisoid arrangement relative to each other and act as bridging ligands between two Pd centers, with a Pd1-N-Pd2 angle of 104.6(4)°. The Pd1-N and Pd2-N bond distances are 1.945(8) Å and 1.967(7) Å, respectively, which fall within the typical range for Pd-N bonds where N is part of a bridging azavinylidene ligand [84,85,86,87,88]. In cases where the ligand is terminal, it has been reported that the Pd-N bond is slightly longer [2.016(7) Å] [87]. The C7-N bond length is 1.273(11) Å, within the tabulated range for Csp^2^=N bonds [1.279(8) Å] [89], and is significantly shorter than the reported values for Csp^3^-NH_2_ bonds [1.469(10) Å] [89], supporting the presence of a multiple C=N bond.

The nitrogen atom is in a trigonal planar environment, where the nitrogen atom is displaced by 0.0280(77) Å from the least-squares plane defined by the atoms bonded to the nitrogen, and the sum of the angles around it is 360.1(3)°. On the other hand, the Cα atom (C7) is also in a trigonal planar environment, displaced by 0.0120(7) Å, and where the sum of the angles around it is 360.0(9)°. These data demonstrate that the Cα exhibits sp^2^ hybridization, forming a covalent double bond with the nitrogen, which also shows sp^2^ hybridization. This indicates that the chiral information at the asymmetric center of the aminoester fragment has been lost. The aromatic ring shows an ortho substitution by a methoxy group as a result of the oxidative coupling with methanol and the formation of the C-O bond.

**Figure 7:**
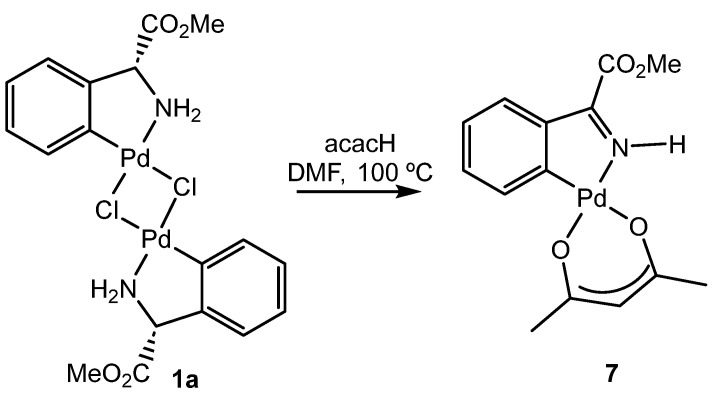
Synthesis of **7**: stabilization of the imine ligand formed from degradation of ortho-metalated phenylglycine **1a** by the chelating acetylacetonate ligand.

An additional way to demonstrate the formation of imines during the degradation of phenylglycine is to carry out the decomposition of complex **1a** at high temperature in the presence of a ligand that captures and stabilizes the transiently formed organometallic species. The reason for performing the reaction at high temperature is the previous observation that decomposition accelerates at higher temperatures compared to the reaction at room temperature. Therefore, complex **1a** was heated in *N*,*N*-dimethylformamide at 100 °C in the presence of acetylacetone, a chelating agent that provides high stability to the complexes it coordinates with. The process is outlined in Figure 7.

**Figure 8:**
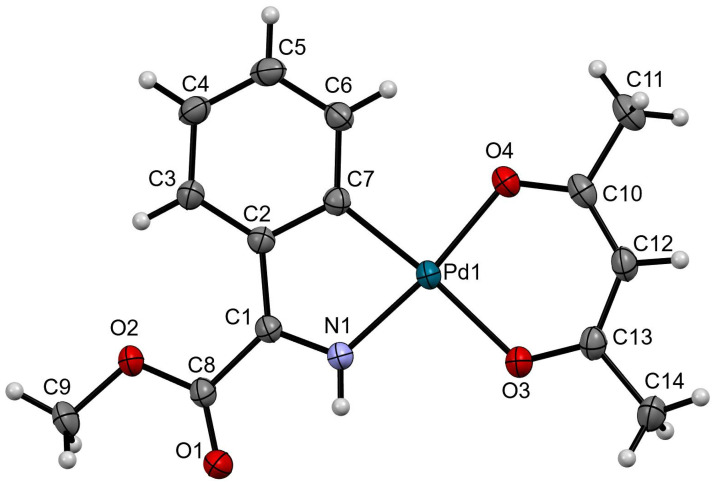
Structure of derivative **7**. Ellipsoids of non-hydrogen atoms have been drawn at 50% probability.

From the reaction mixture, complex **7** is isolated with a yield of 22%, characterized by its analytical and spectroscopic data and by X-ray diffraction. The ^1^H NMR spectrum shows, in the aromatic region, an AMXZ spin system (two doublets at 7.95 and 7.63 ppm, and two triplets at 7.30 and 7.09 ppm) assigned to the 1,2-disubstituted C_6_H_4_ ring. In the high-field region, signals corresponding to the acac ligand (5.43, 2.11, and 2.04 ppm) and CO_2_Me (4.01 ppm) are observed, but no signals corresponding to the H-Cα or NH_2_ groups are detected. Instead, a low-field resonance (9.85 ppm) is observed, which integrates for a single hydrogen and does not correlate with any ^13^C in the ^1^H-^13^C HSQC spectrum. These data suggest a structure like the one represented in Figure 7. Confirmation of this structure was achieved by determining its molecular structure through X-ray diffraction, as shown in Figure 8.

Compound **7** crystallizes in the centrosymmetric triclinic space group *P*-1. The palladium atom is in a square-planar environment, contained within the plane that best fits, by least squares, the one defined by atoms C7, N1, O3, and O4. The sum of the angles around Pd is 360.03(6)°. The Pd1-N1 bond distance is 1.9832(14) Å, which falls within the typical range for imines coordinated to palladium [90,91]. The Pd1-O4 bond distance [2.0048(12) Å], trans to N1, is shorter than the Pd1-O3 bond distance [2.0806(13) Å], trans to C7, due to the stronger trans influence exerted by the phenyl group compared to the imino group. The C1-N1 bond length is 1.287(2) Å, a value consistent with typical Csp^2^=N distances [1.279(8) Å] [89], but significantly shorter than the published values for single Csp^3^-NH_2_ bonds [1.469(10) Å] [89], confirming the presence of a C=N double bond in compound **7**.

Both the N1 and C1 atoms are in environments with trigonal planar geometry, which is only possible with sp^2^ hybridization. The formation of the chelate slightly distorts the angles around the C1 atom [115.3(1)°, 116.2(1)°, and 128.5(1)°], although their sum is 360.0(1)°. The C1 atom deviates by 0.0037(1) Å from the plane that best fits, by least squares, the one defined by the C2, C8, and N1 atoms, to which it is covalently bonded. Overall, the molecule exhibits perfect planarity. This characteristic, along with the high degree of delocalization of π-electrons in the molecular orbitals, imparts a certain degree of aromaticity, which provides special stability and explains why this primary metallic ketimine is air-stable. The conjugated electronic delocalization between the π-electrons of the organic fragment and those of the metal center, as observed in **7**, is known as metalloaromaticity. By analogy to aromatic rings, this phenomenon enhances the stabilization of the formed species [92,93].

## 3. Materials and Methods

### 3.1. General Methods

Solvents were obtained from commercial sources and used without further purification. All reactions were performed without special cautions against water and moisture. Thin-layer chromatography (TLC) was performed on Macherey-Nagel Polygram^®^ SIL G/UV254 silica gel over polyester sheets, with manganese-activated zinc silicate with green fluorescence for short-wave UV (254 nm) and special inorganic fluorescent pigment with blue fluorescence for long-wave UV (366 nm) as indicators. Fluka silica gel (pore size 60 Å, 70–230 mesh, 63–200 μm) was used for gravity column chromatography. Optical rotations were obtained on a JASCO P-1020 polarimeter, and the values were reported as absolute rotations: [α]_D_^T^ (concentration, c, in g/100 mL of solvent), where T stands for the temperature in Kelvin and D is the D line of sodium (589 nm). C, H, N, and S elemental microanalyses were carried out on a PerkinElmer 2400-B Series II Analyzer. Electrospray ionization (ESI) mass spectra were recorded using a Bruker Esquire3000 plus^TM^ ion-trap mass spectrometer equipped with a standard ESI source. Matrix-assisted laser desorption ionization (MALDI) mass spectra were recorded from dichloromethane solutions using dithranol (DIT) as a matrix on a Bruker MicroFlex^TM^ spectrometer or on a Bruker AutoFlex^TM^ III spectrometer, equipped with a time-of-flight (ToF) mass analyzer. High-resolution mass spectra-ESI (HRMS-ESI) mass spectra were recorded using a Bruker MicroToF-Q^TM^ equipped with an API-ESI source and a Q-ToF mass analyzer, which allows it to reach a maximum error in the measurement of 5 ppm. Acetonitrile and methanol were used as solvents. For all types of MS measurements, samples were introduced on a continuous flow of 0.2 mL/min, and nitrogen served both as the nebulizer gas and the dry gas. Infrared spectra were recorded on a Spectrum 100 PerkinElmer FTIR Spectrophotometer with a Universal Attenuated Total Reflectance (UATR) accessory made by thallium bromide–iodide crystals (KRS-5), which allows the observation of the electromagnetic spectrum in the region comprising between 4000 and 250 cm^−1^. The ^1^H, ^13^C{^1^H}, and ^31^P{^1^H} NMR spectra were recorded on a Bruker Avance-300 spectrometer (δ in ppm; J in Hz). All the experiments were recorded on solution, using CDCl_3_ as the deuterated solvent. The ^1^H and ^13^C{^1^H} spectra were referenced using the residual solvent signal as an internal standard, while the ^31^P{^1^H} spectra were referenced to H_3_PO_4_ (85%). All the experiments were recorded at 298 K (different conditions will be indicated). Assignment was performed, when necessary, with the help of the following 2D-NMR experiments: ^1^H-^1^H gradient-selected correlation spectroscopy (gCOSY), ^1^H-^13^C heteronuclear single quantum coherence (HSQC), ^1^H-^13^C heteronuclear multiple bond correlation (HMBC), and ^1^H-^1^H nuclear Overhauser enhancement spectroscopy (NOESY) experiments. The starting dinuclear complexes **1a**–**1g** [65,68] and the chlorinating reagent PhICl_2_ [70] were prepared following methods reported previously.

### 3.2. X-Ray Crystallography

#### 3.2.1. Data Collection

One selected crystal (size as indicated on each case) was mounted at the end of a quartz fiber in a random orientation, covered with perfluorinated oil (magic oil), and placed under a cold stream of nitrogen gas. Crystallographic measurements were carried out at 100 K (**6**) or 173 K (**7**) on a Bruker Smart APEX CCD diffractometer, using graphite monochromated Mo-Kα radiation (λ = 0.71073 Å). A hemisphere of data was collected in each case based on ω scan or φ scan runs. The diffraction frames were integrated using the program SAINT [94] and the integrated intensities were corrected for absorption with SADABS [95].

#### 3.2.2. Structure Solution and Refinement

The structures were solved and developed using the Patterson and Fourier methods [96,97]. All non-hydrogen atoms were refined with anisotropic displacement parameters. The hydrogen atoms were placed at idealized positions and treated as riding atoms. Each hydrogen atom was assigned an isotropic displacement parameter equal to 1.2 to 1.5 times the equivalent isotropic displacement parameter of its parent atom. The structures were refined to F_o_^2^, and all reflections were used in the least-squares calculations [98,99]. CCDC-1969499 (**6**) and 2408386 (**7**) contain the supplementary crystallographic data for this paper. These data can be obtained free of charge from the Cambridge Crystallographic Data Centre via www.ccdc.cam.ac.uk/structures/ (accessed 6 January 2025).

### 3.3. General Halogenation Procedure

To a suspension of the orthopalladated amino ester derivatives (**1a**–**1g**) (1 equiv.) in dichloromethane (10 mL), the halogenating reagent (freshly prepared PhICl_2_, **2a**; Br_2_ **2b** or I_2_, **2c**) (2 equiv.) was added. The reaction mixture was stirred for 20 h at room temperature and then filtered through a plug of celite. The resulting solution was concentrated under vacuum to ca. 10 mL, washed with a 10% sodium sulfite solution (3 × 15 mL) and with a saturated NaCl solution (2 × 10 mL). The resulting clear solution was reacted with the stoichiometric amount of 1,10-phenanthroline monohydrate (1 equiv.), giving almost instantaneously a yellow precipitate of Cl_2_Pd(phen). This suspension was stirred for 3 h at room temperature and then filtered to remove the Pd complex. The resulting solution was evaporated to dryness, and the residue was purified by column chromatography using silica as support (solvents were specified for each particular case), affording the corresponding amino esters (**3aa**–**3gc**) as yellow oils.

#### 3.3.1. Synthesis of Methyl-2′-(chloro)phenylglycinate (**3aa**)

Following the general procedure, compound **1a** (100.0 mg, 0.163 mmol) was reacted with freshly prepared PhICl_2_, **2a**, (89.8 mg, 0.326 mmol) in CH_2_Cl_2_ (10 mL) to give, after treatment with 1,10-phenanthroline monohydrate (32.4 mg, 0.163 mmol) and chromatographic purification (ethyl acetate), **3aa** as a yellow oil. The following were obtained: 64.0 mg, 0.320 mmol, 98% yield. Compound **3aa** is known [71], therefore it has been characterized by the comparison of its spectral data with those previously published.

#### 3.3.2. Synthesis of Methyl 2′-(Iodo)-6′-(bromo)phenylglycinate (**3bc**)

Following the general procedure, compound **1b** (100.0 mg, 0.130 mmol) was reacted with iodine, **2c**, (65.9 mg, 0.260 mmol) in CH_2_Cl_2_ (10 mL) to give, after treatment with 1,10-phenanthroline monohydrate (25.8 mg, 0.130 mmol) and chromatographic purification (*n*-hexane:ethyl acetate, 50:50), **3bc** as a yellow oil. Obtained: 24.1 mg, 0.065 mmol, 25% yield. Elemental analysis: Calc. for C_9_H_9_BrINO_2_ (369.98): C, 29.22; H, 2.45; N, 3.79. Exp.: C, 29.30; H, 2.53; N, 3.82. Mass spect. (ESI+) [*m*/*z*]: 369.9 [M + H]^+^. IR (ν, cm^−1^): 1737 (νC=O). ^1^H NMR (300.13 MHz, CDCl_3_): δ = 7.85 (dd, *J* = 7.9, 1.1 Hz, 1H, C_6_H_3_), 7.56 (dd, *J* = 8.0, 1.0 Hz, 1H, C_6_H_3_), 6.81 (t, *J* = 7.9 Hz, 1H, C_6_H_3_), 5.31 (br s, 1H, CH), 3.74 (s, 3H, OCH_3_), 2.08 (br s, 2H, NH_2_). ^13^C{^1^H} NMR (75.47 MHz, CDCl_3_): δ = 173.4 (s, CO), 141.5 (s, C, C_6_H_3_), 139.8 (s, CH, C_6_H_3_), 134.3 (s, CH, C_6_H_3_), 130.6 (s, CH, C_6_H_3_), 123.0 (s, C-Br, C_6_H_3_), 101.0 (s, C-I, C_6_H_3_), 64.9 (s, CH), 53.1 (s, OCH_3_).

#### 3.3.3. Synthesis of Methyl 5′-(Bromo)-2′-(iodo)phenylglycinate (**3cc**)

Following the general procedure, compound **1c** (50.0 mg, 0.065 mmol) was reacted with iodine, **2c**, (33.0 mg, 0.130 mmol) in CH_2_Cl_2_ (10 mL) to give, after treatment with 1,10-phenanthroline monohydrate (12.9 mg, 0.065 mmol) and chromatographic purification (*n*-hexane:ethyl acetate, 70:30), **3cc** as a yellow oil. Obtained: 13.0 mg, 0.035 mmol, 27% yield. HRMS (ESI+) [*m*/*z*]: Calc. for C_9_H_10_BrINO_2_ [M + H]^+^: 369.8934. Exp.: 369.8931. IR (ν, cm^−1^): 1736 (νC=O). ^1^H NMR (300.13 MHz, CDCl_3_): δ = 7.70 (d, *J* = 8.3 Hz, 1H, C_6_H_3_), 7.47 (d, *J* = 2.0 Hz, 1H, C_6_H_3_), 7.12 (dd, *J* = 8.3, 2.0 Hz, 1H, C_6_H_3_), 4.99 (s, 1H, CH), 3.74 (s, 3H, OCH_3_), 1.25 (s, 2H, NH_2_). ^13^C{^1^H} NMR (75.47 MHz, CDCl_3_): δ = 173.0 (s, CO), 143.5 (s, CH, C_6_H_3_), 141.2 (s, CH, C_6_H_3_), 132.9 (s, CH, C_6_H_3_), 131.0 (s, CH, C_6_H_3_), 123.3 (s, C-Br, C_6_H_3_), 97.9 (s, C-I, C_6_H_3_), 65.1 (s, CH), 52.9 (s, OCH_3_).

#### 3.3.4. Synthesis of Methyl 4′-(Bromo)-2′-(iodo)-phenylglycinate (**3dc**)

Following the general procedure, compound **1d** (50.0 mg, 0.065 mmol) was reacted with iodine, **2c**, (33.0 mg, 0.130 mmol) in CH_2_Cl_2_ (10 mL) to give, after treatment with 1,10-phenanthroline monohydrate (12.9 mg, 0.065 mmol) and chromatographic purification (*n*-hexane:ethyl acetate, 70:30), **3dc** as a yellow oil. Obtained: 15.7 mg, 0.042 mmol, 33% yield. HRMS (ESI+) [*m*/*z*]: Calc. for C_9_H_10_BrINO_2_ [M + H]^+^: 369.8934. Exp.: 369.8939. IR (ν, cm^−1^): 1734 (νC=O). ^1^H NMR (300.13 MHz, CDCl_3_): δ = 8.01 (d, *J* = 2.0 Hz, 1H, C_6_H_3_), 7.48 (dd, *J* = 8.3, 2.0 Hz, 1H, C_6_H_3_), 7.19 (d, *J* = 8.3 Hz, 1H, C_6_H_3_), 4.90 (s, 1H, CH), 3.72 (s, 3H, OCH_3_), 1.99 (br s, 2H, NH_2_). ^13^C{^1^H} NMR (75.47 MHz, CDCl_3_): δ = 173.5 (s, CO), 142.3 (s, C, C_6_H_3_), 141.9 (s, CH, C_6_H_3_), 132.1 (s, CH, C_6_H_3_), 128.8 (s, CH, C_6_H_3_), 122.6 (s, C-Br, C_6_H_3_), 100.3 (s, C-I, C_6_H_3_), 62.2 (s, CH), 52.8 (s, OCH_3_).

#### 3.3.5. Synthesis of Methyl *N*,*N*-Dimethyl-2′-(iodo)phenylglycinate (**3ec**)

Following the general procedure, compound **1e** (30.0 mg, 0.045 mmol) was reacted with iodine, **2c**, (22.8 mg, 0.090 mmol) in CH_2_Cl_2_ (10 mL) to give, after treatment with 1,10-phenanthroline monohydrate (8.9 mg, 0.045 mmol) and chromatographic purification (*n*-hexane:ethyl acetate, 70:30), **3ec** as a yellow oil. Obtained: 12.3 mg, 43% yield. HRMS (ESI+) [*m*/*z*]: Calc. for C_11_H_15_INO_2_ [M + H]^+^: 320.0142. Exp.: 320.0142. IR (ν, cm^−1^): 1736 (νC=O). ^1^H NMR (300.13 MHz, CDCl_3_): δ = 7.87 (dd, *J* = 7.9, 1.2 Hz, 1H, C_6_H_4_), 7.58 (dd, *J* = 7.8, 1.7 Hz, 1H, C_6_H_4_), 7.35 (td, *J* = 7.5, 1.1 Hz, 1H, C_6_H_4_), 7.00 (ddd, *J* = 7.9, 7.3, 1.7 Hz, 1H, C_6_H_4_), 4.42 (s, 1H, CH), 3.70 (s, 3H, OCH_3_), 2.31 (s, 6H, NMe_2_). ^13^C{^1^H} NMR (75.47 MHz, CDCl_3_): δ = 171.9 (s, CO), 140.0 (s, CH, C_6_H_4_), 139.2 (s, C, C_6_H_4_), 130.0 (s, CH, C_6_H_4_), 129.7 (s, CH, C_6_H_4_), 128.7 (s, CH, C_6_H_4_), 101.8 (s, C-I, C_6_H_4_), 77.0 (s, CH), 52.2 (s, OCH_3_), 43.2 (s, NMe_2_).

#### 3.3.6. Synthesis of Methyl α-Methyl-2′-(chloro)phenylglycinate (**3fa**)

Following the general procedure, compound **1f** (100.0 mg, 0.156 mmol) was reacted with freshly prepared PhICl_2_, **2a**, (85.9 mg, 0.312 mmol) in CH_2_Cl_2_ (10 mL) to give, after treatment with 1,10-phenanthroline monohydrate (31.0 mg, 0.156 mmol) and chromatographic purification (ethyl acetate), **3fa** as a yellow oil. Obtained: 66.0 mg, 0.309 mmol, 99% yield. Elemental analysis: Calc. for C_10_H_12_ClNO_2_ (213.66): C, 56.22; H, 5.66; N, 6.56. Exp.: C, 56.14; H, 5.68; N, 6.61. Mass spect. (ESI+) [*m*/*z*]: 213.9 [M + H]^+^. IR (ν, cm^−1^): 1729 (νC=O). ^1^H NMR (300.13 MHz, CDCl_3_): δ = 7.69 (d, *J* = 7.6 Hz, 1H, C_6_H_4_), 7.31 (t, *J* = 7.8 Hz, 1H, C_6_H_4_), 7.29–7.16 (m, 2H, C_6_H_4_), 3.68 (s, 3H, OCH_3_), 2.10 (s, 2H, NH_2_), 1.69 (s, 3H, CH_3_). ^13^C{^1^H} NMR (75.47 MHz, CDCl_3_): δ = 177.4 (s, CO), 141.2 (s, C, C_6_H_4_), 132.7 (s, C, C_6_H_4_), 130.5 (s, CH, C_6_H_4_), 128.9 (s, CH, C_6_H_4_), 127.1 (s, CH, C_6_H_4_), 127.0 (s, CH, C_6_H_4_), 60.1 (s, C), 52.9 (s, OCH_3_), 26.0 (s, CH_3_).

#### 3.3.7. Synthesis of Methyl α-Methyl-2′-(bromo)phenylglycinate (**3fb**)

Following the general procedure, compound **1f** (100.0 mg, 0.156 mmol) was reacted with bromine, **2b**, (16.0 μL, 0.312 mmol) in CH_2_Cl_2_ (10 mL) to give, after treatment with 1,10-phenanthroline monohydrate (31.0 mg, 0.156 mmol) and chromatographic purification (ethyl acetate), **3fb** as a yellow oil. Obtained: 80.3 mg, 0.311 mmol, 99% yield. Elemental analysis: Calc. for C_10_H_12_BrNO_2_ (258.12): C, 46.53; H, 4.69; N, 5.43. Exp.: C, 46.48; H, 4.60; N, 5.48. Mass spect. (ESI+) [*m*/*z*]: 257.9 [M + H]^+^. IR (ν, cm^−1^): 1728 (νC=O). ^1^H NMR (300.13 MHz, CDCl_3_): δ = 7.71 (dd, *J* = 7.9, 1.7 Hz, 1H, C_6_H_4_), 7.54 (dd, *J* = 7.9, 1.4 Hz, 1H, C_6_H_4_), 7.34 (td, *J* = 7.5, 1.4 Hz, 1H, C_6_H_4_), 7.14 (td, *J* = 7.7, 1.7 Hz, 1H, C_6_H_4_), 3.70 (s, 3H, OCH_3_), 2.13 (s, 2H, NH_2_), 1.72 (s, 3H, CH_3_). ^13^C{^1^H} NMR (75.47 MHz, CDCl_3_): δ = 177.4 (s, CO), 142.7 (s, C, C_6_H_4_), 134.1 (s, CH, C_6_H_4_), 129.1 (s, CH, C_6_H_4_), 127.7 (s, CH, C_6_H_4_), 127.4 (s, CH, C_6_H_4_), 122.6 (s, C-Br, C_6_H_4_), 61.4 (s, C), 52.9 (s, OCH_3_), 26.4 (s, CH_3_).

#### 3.3.8. Synthesis of Methyl α-Methyl-2′-(iodo)phenylglycinate (**3fc**)

Following the general procedure, compound **1f** (100.0 mg, 0.156 mmol) was reacted with iodine, **2c**, (79.3 mg, 0.312 mmol) in CH_2_Cl_2_ (10 mL) to give, after treatment with 1,10-phenanthroline monohydrate (31.0 mg, 0.156 mmol) and chromatographic purification (ethyl acetate), **3fc** as a yellow oil. Obtained: 52.2 mg, 0.171 mmol, 55% yield. Elemental analysis: Calc. for C_10_H_12_INO_2_ (305.12): C, 39.37; H, 3.96; N, 4.59. Exp.: C, 39.45; H, 3.99; N, 4.54. Mass spect. (ESI+) [*m*/*z*]: 305.9 [M + H]^+^. IR (ν, cm^−1^):1729 (νC=O). ^1^H NMR (300.13 MHz, CDCl_3_): δ = 7.88 (d, *J* = 7.9 Hz, 1H, C_6_H_4_), 7.71 (d, *J* = 7.9 Hz, 1H, C_6_H_4_), 7.38 (t, *J* = 7.6 Hz, 1H, C_6_H_4_), 6.97 (t, *J* = 7.5 Hz, 1H, C_6_H_4_), 3.73 (s, 3H, OCH_3_), 2.04 (s, 2H, NH_2_), 1.75 (s, 3H, CH_3_). ^13^C{^1^H} NMR (75.47 MHz, CDCl_3_): δ = 177.2 (s, CO), 145.2 (s, C, C_6_H_4_), 141.5 (s, CH, C_6_H_4_), 129.2 (s, CH, C_6_H_4_), 128.3 (s, CH, C_6_H_4_), 127.2 (s, CH, C_6_H_4_), 96.7 (s, C-I, C_6_H_4_), 63.0 (s, C), 53.0 (s, OCH_3_), 26.9 (s, CH_3_)

#### 3.3.9. Synthesis of Methyl α-Benzyl-2′-(chloro)phenylglycinate (**3ga**)

Following the general procedure, compound **1g** (100.0 mg, 0.127 mmol) was reacted with freshly prepared PhICl_2_, **2a**, (69.6 mg, 0.253 mmol) in CH_2_Cl_2_ (10 mL) to give, after treatment with 1,10-phenanthroline monohydrate (25.2 mg, 0.127 mmol) and chromatographic purification (*n*-hexane:dichloromethane, 20:80), **3ga** as a yellow oil. Obtained: 71.4 mg, 0.246 mmol, 97% yield. Elemental analysis: Calc. for C_16_H_16_ClNO_2_ (289.76): C, 66.32; H, 5.57; N, 4.83. Exp.: C, 66.28; H, 5.46; N, 4.78. Mass spect. (ESI+) [*m*/*z*]: 289.9 [M + H]^+^. IR (ν, cm^−1^): 1740 (νC=O). ^1^H NMR (300.13 MHz, CDCl_3_): δ = 7.83 (m, 1H, C_6_H_4_), 7.53–7.44 (m, 2H, C_6_H_4_), 7.39 (m, 1H, C_6_H_4_), 7.33–7.26 (m, 4H, C_6_H_5_), 7.23 (m, 1H, C_6_H_5_), 4.37 (m, 2H, NH_2_), 3.51 (s, 3H, OCH_3_), 3.40–3.14 (m, 2H, CH_2_). ^13^C{^1^H} NMR (75.47 MHz, CDCl_3_): δ = 171.8 (s, CO), 136.5 (s, C, C_6_H_5_), 134.3 (s, C, C_6_H_4_), 134.2 (s, C, C_6_H_4_), 131.0 (s, CH, Ar), 130.9 (s, CH, Ar), 130.6 (s, CH, Ar), 130.5 (s, CH, C_6_H_4_), 130.4 (s, CH, Ar), 128.9 (s, CH, C_6_H_4_), 128.0 (s, CH, Ar), 127.1 (s, CH, Ar), 126.9 (s, CH, Ar), 66.8 (s, C), 53.0 (s, OCH_3_), 41.0 (s, CH_2_).

#### 3.3.10. Synthesis of Methyl α-Benzyl-2′-(bromo)phenylglycinate (**3gb**)

Following the general procedure, compound **1g** (100.0 mg, 0.127 mmol) was reacted with bromine, **2b**, (13.0 μL, 0.253 mmol) in CH_2_Cl_2_ (10 mL) to give, after treatment with 1,10-phenanthroline monohydrate (25.2 mg, 0.127 mmol) and chromatographic purification (ethyl acetate), **3gb** as a yellow oil. Obtained: 83.9 mg, 0.251 mmol, 99% yield. Elemental analysis: Calc. for C_16_H_16_BrNO_2_ (334.21): C, 57.50; H, 4.83; N, 4.19. Exp.: C, 57.43; H, 4.80; N, 4.22. Mass spect. (ESI+) [*m*/*z*]: 334.0 [M + H]^+^. IR (ν, cm^−1^): 1727 (νC=O). ^1^H NMR (300.13 MHz, CDCl_3_): δ = 7.69 (d, *J* = 7.8 Hz, 1H, C_6_H_4_), 7.48 (d, *J* = 7.6 Hz, 1H, C_6_H_4_), 7.31–7.20 (m, 5H, 2 C_6_H_4_ + 3 C_6_H_5_), 6.98 (m, 2H, C_6_H_5_), 3.81 (s, 3H, OCH_3_), 3.75, 3.49 (AB spin system, *J* = 13.4 Hz, 2H, CH_2_), 2.07 (s, 2H, NH_2_). ^13^C{^1^H} NMR (75.47 MHz, CDCl_3_): δ = 175.6 (s, CO), 141.3 (s, C, C_6_H_4_), 135.6 (s, C, C_6_H_5_), 134.0 (s, CH, C_6_H_4_), 130.7 (s, CH, C_6_H_5_), 129.2 (s, CH, C_6_H_4_), 128.5 (s, CH, C_6_H_4_), 127.8 (s, CH, C_6_H_5_), 127.2 (s, CH, C_6_H_4_), 126.8 (s, CH, C_6_H_5_), 122.4 (s, C-Br, C_6_H_4_), 64.6 (s, C), 52.7 (s, OCH_3_), 42.6 (s, CH_2_).

#### 3.3.11. Synthesis of Methyl α-Benzyl-2′-(iodo)phenylglycinate (**3gc**)

Following the general procedure, compound **1g** (100.0 mg, 0.127 mmol) was reacted with iodine, **2c**, (64.3 mg, 0.253 mmol) in CH_2_Cl_2_ (10 mL) to give, after treatment with 1,10-phenanthroline monohydrate (25.2 mg, 0.127 mmol) and chromatographic purification (ethyl acetate), **3gc** as a yellow oil. Obtained: 95.8 mg, 0.251 mmol, 98% yield. Elemental analysis: Calc. for C_16_H_16_INO_2_ (381.21): C, 50.41; H, 4.23; N, 3.67. Exp.: C, 50.37; H, 4.19; N, 3.69. Mass spect. (ESI+) [*m*/*z*]: 381.9 [M + H]^+^. IR (ν, cm^−1^): 1724 (νC=O). ^1^H NMR (300.13 MHz, CDCl_3_): δ = 8.04 (d, *J* = 7.8 Hz, 1H, C_6_H_4_), 7.45 (d, *J* = 8.1 Hz, 1H, C_6_H_4_), 7.32 (t, *J* = 7.8 Hz, 1H, C_6_H_4_), 7.28–7.22 (m, 3H, C_6_H_5_), 7.05 (t, *J* = 7.7 Hz, 1H, C_6_H_4_), 6.97 (m, 2H, C_6_H_5_), 3.84 (s, 3H, OCH_3_), 3.83, 3.48 (AB spin system, *J* = 13.0 Hz, 2H, CH_2_), 2.07 (s, 2H, NH_2_). ^13^C{^1^H} NMR (75.47 MHz, CDCl_3_): δ = 175.4 (s, CO), 143.8 (s, C, C_6_H_4_), 141.5 (s, CH, C_6_H_4_), 135.7 (s, C, C_6_H_5_), 130.7 (s, CH, C_6_H_5_), 129.3 (s, CH, C_6_H_4_), 128.5 (s, CH, C_6_H_4_), 127.9 (s, CH, C_6_H_4_), 127.8 (s, CH, C_6_H_5_), 126.8 (s, CH, C_6_H_5_), 96.7 (s, C-I, C_6_H_4_), 66.2 (s, C_α_), 52.9 (s, OCH_3_), 42.7 (s, CH_2_).

### 3.4. General Alkoxylation Procedure

To a suspension of *ortho*-metallated complex **1a** (1 equivalent) in the corresponding alcohol, (**4a**)–(**4i**) (10 mL), PhI(OAc)_2_ (4 equivalents) was added. The mixture was stirred for 20 h at room temperature, and the resulting brown suspension was evaporated to dryness under vacuum. The residue was redissolved in dichloromethane (10 mL) and filtered through a plug of celite to remove any insoluble solid. The clear orange solution was washed with sodium sulfite 10% (3 × 15 mL) and with saturated sodium chloride solution (2 × 10 mL). The organic phase was dried over anhydrous magnesium sulfate, filtered, and evaporated to dryness. The residue contains alkoxylated amino ester derivatives, (**5aa**)–(**5ai**), as a ligand coordinated to palladium, PdCl_2_L_2_. This residue was purified by silica column chromatography as specified in each case. The fraction containing the coordination complex, PdCl_2_L_2_, was redissolved in dichloromethane (10 mL) and was treated for 3 h at room temperature with the stoichiometric amount of 1,10-phenanthroline monohydrate (1 equivalent). During this time, PdCl_2_(1,10-phen) precipitated, which was removed by filtration. The resulting yellow solution was evaporated to dryness, and diethyl ether (10 mL) was added, generating a suspension that was again filtered and evaporated to dryness, affording the corresponding ortho-alkoxylated amino esters, (**5aa**)–(**5ai**), as yellow oils.

#### 3.4.1. Synthesis of (*R*)-Methyl 2′-(1-Propoxy)phenylglycinate (**5aa**)

Following the general alkoxylation procedure, PhI(OAc)_2_ (421.0 mg, 1.307 mmol) was reacted with **1a** (200.0 mg, 0.327 mmol) in 1-propanol, **4a**, (10 mL) to give, after the chromatographic purification (*n*-hexane:ethyl acetate, 50:50) of the metallic intermediate and treatment with 1,10-phenanthroline monohydrate (64.8 mg, 0.327 mmol), **5aa** as a yellow oil. Obtained: 69.4 mg, 0.311 mmol, 48% yield (87% ee). Optical rotation: [α]_D_^298^ = −45.0 (CHCl_3_, c = 1.90). Elemental analysis: Calc. for C_12_H_17_NO_3_0.17 H_2_O (223.27 + 3.06): C, 63.68; H, 7.72; N, 6.19. Exp.: C, 63.67; H, 7.71; N, 6.51. Mass spect. (ESI+) [*m*/*z*]: 224.0 [M + H]^+^. IR (ν, cm^−1^): 2961, 2925 (νNH_2_), 1738 (νC=O). ^1^H NMR (400.13 MHz, CDCl_3_): δ = 7.24 (m, 2H, C_6_H_4_), 6.93 (td, *J* = 7.6, 1.0 Hz, 1H, C_6_H_4_), 6.86 (d, *J* = 7.8 Hz, 1H, C_6_H_4_), 4.68 (s, 1H, CH), 3.93 (tt, *J* = 6.1, 3.2 Hz, 2H, OCH_2_), 3.69 (s, 3H, OCH_3_), 1.96 (br s, 2H, NH_2_), 1.80 (m, 2H, CH_2_), 1.04 (t, *J* = 7.4 Hz, 3H, CH_3_). ^13^C{^1^H} NMR (100.61 MHz, CDCl_3_): δ = 175.1 (s, CO), 156.3 (s, C, C_6_H_4_), 129.2 (s, CH, C_6_H_4_), 129.1 (s, CH, C_6_H_4_), 120.8 (s, CH, C_6_H_4_), 111.7 (s, CH, C_6_H_4_), 69.7 (s, OCH_2_), 55.7 (s, CH), 52.3 (s, OCH_3_), 22.7 (s, CH_2_), 10.8 (s, CH_3_). The signal due to a quaternary carbon of the C_6_H_4_ ring was not observed, in spite of the use of long accumulation trials.

#### 3.4.2. Synthesis of (*R*)-Methyl 2′-(2-Propoxy)phenylglycinate (**5ab**)

Following the general alkoxylation procedure, PhI(OAc)_2_ (421.0 mg, 1.307 mmol) was reacted with **1a** (200.0 mg, 0.327 mmol) in 2-propanol, **4b**, (10 mL) to give, after the chromatographic purification (ethyl acetate) of the metallic intermediate and treatment with 1,10-phenanthroline monohydrate (64.8 mg, 0.327 mmol), **5ab** as a yellow oil. Obtained: 87.1 mg, 0.390 mmol, 60% yield (91% ee). Optical rotation: [α]_D_^298^ = +25.7 (CHCl_3_, c = 0.01). Elemental analysis: Calc. for C_12_H_17_NO_3_1.07 H_2_O (223.27 + 19.28): C, 59.42; H, 7.95; N, 5.77. Exp.: C, 59.43; H, 7.81; N, 5.90. Mass spect. (ESI+) [*m*/*z*]: 224.2 [M + H]^+^. IR (ν, cm^−1^): 2975 (νNH_2_), 1736 (νC=O). ^1^H NMR (400.13 MHz, CDCl_3_): δ = 7.26–7.21 (m, 2H, C_6_H_4_), 6.91 (td, *J* = 7.5, 1.0 Hz, 1H, C_6_H_4_), 6.86 (d, *J* = 8.4 Hz, 1H, C_6_H_4_), 5.29 (s, 1H, CH), 4.61 (q, *J* = 6.8, 6.2 Hz, 1H, OCH), 3.68 (s, 3H, OCH_3_), 1.94 (s, 2H, NH_2_), 1.34 (d, *J* = 6.1 Hz, 3H, CH_3_), 1.29 (d, *J* = 6.0 Hz, 3H, CH_3_). ^13^C{^1^H} NMR (100.61 MHz, CDCl_3_): δ = 175.2 (s, CO), 156.6 (s, C, C_6_H_4_), 129.9 (s, C, C_6_H_4_), 129.5 (s, CH, C_6_H_4_), 129.1 (s, CH, C_6_H_4_), 120.6 (s, CH, C_6_H_4_), 112.6 (s, CH, C_6_H_4_), 69.8 (s, OCH), 56.1 (s, CH), 52.3 (s, OCH_3_), 22.1 (s, CH_3_), 22.0 (s, CH_3_).

#### 3.4.3. Synthesis of (*R*)-Methyl 2′-(1-Butoxy)phenylglycinate (**5ac**)

Following the general alkoxylation procedure, PhI(OAc)_2_ (421.0 mg, 1.307 mmol) was reacted with **1a** (200.0 mg, 0.327 mmol) in 1-butanol, **4c**, (10 mL) to give, after the chromatographic purification (ethyl acetate) of the metallic intermediate and treatment with 1,10-phenanthroline monohydrate (64.8 mg, 0.327 mmol), **5ac** as a yellow oil. Obtained: 77.4 mg, 0.326 mmol, 50% yield (65% ee). Optical rotation: [α]_D_^298^ = −45.0 (CHCl_3_, c = 1.90). Elemental analysis: Calc. for C_13_H_19_NO_3_ (237.29): C, 65.78; H, 8.07; N, 5.91. Exp.: C, 65.70; H, 8.13; N, 5.96. Mass spect. (ESI+) [*m*/*z*]: 238.0 [M + H]^+^. IR (ν, cm^−1^): 2955 (νNH_2_), 1736 (νC=O). ^1^H NMR (400.13 MHz, CDCl_3_): δ = 7.23 (m, 2H, C_6_H_4_), 6.92 (td, *J* = 7.5, 0.8 Hz, 1H, C_6_H_4_), 6.86 (d, *J* = 8.0 Hz, 1H, C_6_H_4_), 4.67 (s, 1H, CH), 3.96 (m, 2H, OCH_2_), 3.67 (s, 3H, OCH_3_), 2.25 (br s, 2H, NH_2_), 1.74 (m, 2H, CH_2_), 1.48 (m, 2H, CH_2_), 0.96 (t, *J* = 7.4 Hz, 3H, CH_3_). ^13^C{^1^H} NMR (100.61 MHz, CDCl_3_): δ = 175.0 (s, CO), 156.3 (s, C, C_6_H_4_), 129.2 (s, CH, C_6_H_4_), 129.0 (s, CH, C_6_H_4_), 128.7 (s, C, C_6_H_4_), 120.7 (s, CH, C_6_H_4_), 111.6 (s, CH, C_6_H_4_), 67.7 (s, OCH_2_), 55.6 (s, CH), 52.2 (s, OCH_3_), 31.3 (s, CH_2_), 19.3 (s, CH_2_), 13.9 (s, CH_3_).

#### 3.4.4. Synthesis of (*R*)-Methyl 2′-(2-Methyl-1-propoxy)phenylglycinate (**5ad**)

Following the general alkoxylation procedure, PhI(OAc)_2_ (421.0 mg, 1.307 mmol) was reacted with **1a** (200.0 mg, 0.327 mmol) in 2-methyl-1-propanol, **4d**, (10 mL) to give, after the chromatographic purification (ethyl acetate) of the metallic intermediate and treatment with 1,10-phenanthroline monohydrate (64.8 mg, 0.327 mmol), **5ad** as a yellow oil. Obtained: 63.7 mg, 0.268 mmol, 41% yield (84% ee). Optical rotation: [α]_D_^298^ = −14.8 (CHCl_3_, c = 1.6). Elemental analysis: Calc. for C_13_H_19_NO_3_0.12CHCl_3_ (237.30 + 14.32): C, 62.63; H, 5.57; N, 7.66. Exp.: C, 62.69; H, 5.79; N, 7.29. Mass spect. (ESI+) [*m*/*z*]: 238.0 [M + H]^+^. IR (ν, cm^−1^): 2955 (br, νNH_2_), 1736 (νC=O). ^1^H NMR (400.13 MHz, CDCl_3_): δ = 7.28–7.24 (m, 2H, C_6_H_4_), 6.93 (t, *J* = 7.4 Hz, 1H, C_6_H_4_), 6.86 (d, *J* = 8.5 Hz, 1H, C_6_H_4_), 4.69 (s, 1H, CαH), 3.78 (d, *J* = 6.0 Hz, 2H, OCH_2_), 3.69 (s, 3H, OCH_3_), 2.11 (m, 3H, CH + NH_2_), 1.06 (d, *J* = 3.3 Hz, 3H, CH_3_), 1.04 (d, *J* = 3.3 Hz, 3H, CH_3_). ^13^C{^1^H} NMR (100.61 MHz, CDCl_3_): δ = 175.0 (s, CO), 156.3 (s, C, C_6_H_4_), 129.2 (s, CH, C_6_H_4_), 129.1 (s, CH, C_6_H_4_), 128.9 (s, C, C_6_H_4_), 120.7 (s, CH, C_6_H_4_), 111.4 (s, CH, C_6_H_4_), 74.5 (s, OCH_2_), 55.7 (s, CαH), 52.3 (s, OCH_3_), 28.4 (s, CH), 19.4 (s, CH_3_), 19.3 (s, CH_3_).

#### 3.4.5. Synthesis of (*R*)-Methyl 2′-(3-Methyl-1-butoxy)phenylglycinate (**5ae**)

Following the general alkoxylation procedure, PhI(OAc)_2_ (421.0 mg, 1.307 mmol) was reacted with **1a** (200.0 mg, 0.327 mmol) in 3-methyl-1-butanol, **4e**, (10 mL) to give, after the chromatographic purification (ethyl acetate) of the metallic intermediate and treatment with 1,10-phenanthroline monohydrate (64.8 mg, 0.327 mmol), **5ae** as a yellow oil. Obtained: 86.3 mg, 0.343 mmol, 53% yield (76% ee). Optical rotation: [α]_D_^298^ = −10.5 (CHCl_3_, c = 0.48). Elemental analysis: Calc. for C_14_H_21_NO_3_ (251.33): C, 66.91; H, 8.42; N, 5.57. Exp.: C, 66.92; H, 8.10; N, 5.90. Mass spect. (ESI+) [*m*/*z*]: 252.1 [M + H]^+^. IR (ν, cm^−1^): 2954 (νNH_2_), 1739 (νC=O). ^1^H NMR (400.13 MHz, CDCl_3_): δ = 7.26–7.21 (m, 2H, C_6_H_4_), 6.92 (td, *J* = 7.5, 1.0 Hz, 1H, C_6_H_4_), 6.87 (dd, *J* = 8.1, 4.2 Hz, 1H, C_6_H_4_), 4.67 (s, 1H, CαH), 4.00 (m, 2H, OCH_2_), 3.68 (s, 3H, OCH_3_), 2.00 (br s, 2H, NH_2_), 1.83 (m, 1H, CH), 1.67 (m, 2H, CH_2_), 0.98–0.93 (m, 6H, CH_3_). ^13^C{^1^H} NMR (100.61 MHz, CDCl_3_): δ = 175.0 (s, CO), 156.2 (s, C, C_6_H_4_), 137.6 (s, C, C_6_H_4_), 129.2 (s, CH, C_6_H_4_), 129.0 (s, CH, C_6_H_4_), 120.8 (s, CH, C_6_H_4_), 111.7 (s, CH, C_6_H_4_), 66.5 (s, OCH_2_), 55.6 (s, CαH), 52.3 (s, OCH_3_), 38.0 (s, CH_2_), 25.1 (s, CH), 22.7 (s, CH_3_), 22.6 (s, CH_3_).

#### 3.4.6. Synthesis of (*R*)-Methyl 2′-(Benzyloxy)phenylglycinate (**5af**)

Following the general alkoxylation procedure, PhI(OAc)_2_ (421.0 mg, 1.307 mmol) was reacted with **1a** (200.0 mg, 0.327 mmol) in benzyl alcohol, **4f**, (10 mL) to give, after the chromatographic purification (ethyl acetate:methanol, 99:1) of the metallic intermediate and treatment with 1,10-phenanthroline monohydrate (64.8 mg, 0.327 mmol), **5af** as a yellow oil. Obtained: 50.3 mg, 0.185 mmol, 28% yield (71% ee). Optical rotation: [α]_D_^298^ = −9.3 (CHCl_3_, c = 0.36). Elemental analysis: Calc. for C_16_H_17_NO_3_ (271.32): C, 70.83; H, 6.32; N, 5.16. Exp.: C, 70.88; H, 6.30; N, 4.99. Mass spect. (ESI+) [*m*/*z*]: 272.1 [M + H]^+^. IR (ν, cm^−1^): 3030, 2949 (νNH_2_), 1736 (νC=O). ^1^H NMR (400.13 MHz, CDCl_3_): δ = 7.30–7.28 (m, 3H, C_6_H_5_), 7.25 (m, 2H, C_6_H_5_), 7.19–7.15 (m, 2H, C_6_H_4_), 6.89–6.82 (m, 2H, C_6_H_4_), 5.02 (d, *J* = 11.9 Hz, 1H, OCH_2_), 5.00 (d, *J* = 11.9 Hz, 1H, OCH_2_), 4.67 (s, 1H, CH), 3.53 (s, 3H, OCH_3_), 1.96 (br s, 2H, NH_2_). ^13^C{^1^H} NMR (100.61 MHz, CDCl_3_): δ = 174.9 (s, C, CO), 155.9 (s, C, Ar), 136.8 (s, C, Ar), 129.3 (s, CH, Ar), 128.7 (s, CH, Ar), 128.1 (s, CH, Ar), 127.2 (s, CH, Ar), 127.0 (s, CH, Ar), 121.3 (s, CH, Ar), 112.3 (s, CH, Ar), 70.2 (s, OCH_2_), 55.2 (s, CH), 52.3 (s, OCH_3_). Despite long accumulation trials, one quaternary aromatic carbon was not detected.

#### 3.4.7. Synthesis of (*R*)-Methyl 2′-(3,4-Dimethoxybenzyloxy)phenylglycinate (**5ag**)

Following the general alkoxylation procedure, PhI(OAc)_2_ (421.0 mg, 1.307 mmol) was reacted with **1a** (200.0 mg, 0.327 mmol) in 3,4-dimethoxybenzyl alcohol, **4g**, (10 mL) to give, after the chromatographic purification (ethyl acetate) of the metallic intermediate and treatment with 1,10-phenanthroline monohydrate (64.8 mg, 0.327 mmol), **5ag** as a yellow oil. Obtained: 40.5 mg, 0.122 mmol, 19% yield (93% ee). Optical rotation: [α]_D_^298^ = −28.9 (CHCl_3_, c = 0.76). Elemental analysis: Calc. for C_18_H_21_NO_5_ (331.37): C, 64.24; H, 6.39; N, 4.23. Exp.: C, 64.26; H, 5.97; N, 4.52. Mass spect. (ESI+) [*m*/*z*]: 332.1 [M + H]^+^. IR (ν, cm^−1^): 2949 (br, νNH_2_), 1734 (νC=O). ^1^H NMR (400.13 MHz, CDCl_3_): δ = 7.27 (m, 1H, C_6_H_4_), 7.00–6.92 (m, 5H, C_6_H_4_ + C_6_H_3_), 6.86 (d, *J* = 8.2 Hz, 1H, C_6_H_4_), 5.05 (d, *J* = 12.2 Hz, 1H, OCH_2_), 5.02 (d, *J* = 12.2 Hz, 1H, OCH_2_), 4.75 (s, 1H, CH), 3.92 (s, 3H, Ar-OCH_3_), 3.89 (s, 3H, Ar-OCH_3_), 3.63 (s, 3H, COOCH_3_), 1.91 (s, 2H, NH_2_). ^13^C{^1^H} NMR (100.61 MHz, CDCl_3_): δ = 175.0 (s, CO), 156.0 (s, C, C_6_H_4_), 149.2 (s, C, C_6_H_3_), 148.9 (s, C, C_6_H_3_), 129.35 (s, C, Ar), 129.31 (s, C, Ar), 129.28 (s, CH, Ar), 129.1 (s, CH, Ar), 121.3 (s, CH, Ar), 119.8 (s, CH, Ar), 112.4 (s, CH, Ar), 111.1 (s, CH, Ar), 110.7 (s, CH, Ar), 70.2 (s, OCH_2_), 56.1 (s, OCH_3_), 56.0 (s, OCH_3_), 55.5 (s, CH), 52.3 (s, OCH_3_).

#### 3.4.8. Synthesis of (*R*)-Methyl 2′-(Cyclopentyloxy)phenylglycinate (**5ah**)

Following the general alkoxylation procedure, PhI(OAc)_2_ (421.0 mg, 1.307 mmol) was reacted with **1a** (200.0 mg, 0.327 mmol) in cyclopentanol, **4h**, (10 mL) to give, after the chromatographic purification (ethyl acetate) of the metallic intermediate and treatment with 1,10-phenanthroline monohydrate (64.8 mg, 0.327 mmol), **5ah** as a yellow oil. Obtained: 80.0 mg, 0.321 mmol, 49% yield (84% ee). Optical rotation: [α]_D_^298^ = +10.8 (CHCl_3_, c = 0.03). Elemental analysis: Calc. for C_14_H_19_NO_3_ (249.31): C, 67.45; H, 7.68; N, 5.62. Exp.: 67.21; H, 7.71; N, 5.39. Mass spect. (ESI+) [*m*/*z*]: 250.1 [M + H]^+^. IR (ν, cm^−1^): 3060, 2953 (νNH_2_), 1740 (νC=O). ^1^H NMR (400.13 MHz, CDCl_3_): δ = 7.23 (m, 2H, C_6_H_4_), 6.91 (t, *J* = 7.5 Hz, 1H, C_6_H_4_), 6.86 (d, *J* = 8.5 Hz, 1H, C_6_H_4_), 4.80 (m, 1H, OCH), 4.58 (s, 1H, CH), 3.68 (s, 3H, OCH_3_), 2.00 (br s, 4H, NH_2_ + CH_2_), 1.90–1.86 (m, 2H, CH_2_), 1.81–1.75 (m, 2H, CH_2_), 1.66–1.61 (m, 2H, CH_2_). ^13^C{^1^H} NMR (100.61 MHz, CDCl_3_): δ = 174.8 (s, CO), 154.9 (s, C, C_6_H_4_), 146.1 (s, C, C_6_H_4_), 129.6 (s, CH, C_6_H_4_), 129.1 (s, CH, C_6_H_4_), 120.4 (s, CH, C_6_H_4_), 112.5 (s, CH, C_6_H_4_), 79.3 (s, OCH), 56.2 (s, CH), 52.3 (s, OCH_3_), 33.0 (s, CH_2_), 32.9 (s, CH_2_), 24.2 (s, CH_2_), 24.2 (s, CH_2_).

#### 3.4.9. Synthesis of (*R*)-Methyl 2′-(Cyclohexyloxy)phenylglycinate (**5ai**)

Following the general alkoxylation procedure, PhI(OAc)_2_ (421.0 mg, 1.307 mmol) was reacted with **1a** (200.0 mg, 0.327 mmol) in cyclohexanol, **4i**, (10 mL) to give, after the chromatographic purification (ethyl acetate) of the metallic intermediate and treatment with 1,10-phenanthroline monohydrate (64.8 mg, 0.327 mmol), **5ai** as a yellow oil. Obtained: 92.1 mg, 0.350 mmol, 53% yield (79% ee). Optical rotation: [α]_D_^298^ = −1.2 (CHCl_3_, c = 0.37). Elemental analysis: Calc. for C_15_H_21_NO_3_ (263.34): C, 68.42; H, 8.04; N, 5.32. Exp.: C, 68.40; H, 7.89; N, 5.69. Mass spect. (ESI+) [*m*/*z*]: 264.0 [M + H]^+^. IR (ν, cm^−1^): 2933 (br, νNH_2_), 1739 (νC=O). ^1^H NMR (400.13 MHz, CDCl_3_): δ = 7.23–7.19 (m, 2H, C_6_H_4_), 6.86 (td, *J* = 7.5, 1.0 Hz, 1H, C_6_H_4_), 6.83 (d, *J* = 8.2 Hz, 1H, C_6_H_4_), 4.59 (s, 1H, CH), 4.29 (tt, *J* = 8.2, 3.6 Hz, 1H, OCH), 3.65 (s, 3H, OCH_3_), 2.06 (br s, 2H, NH_2_), 1.92–1.85 (m, 2H, C_6_H_11_), 1.76–1.69 (m, 2H, C_6_H_11_), 1.55–1.46 (m, 3H, C_6_H_11_), 1.38–1.31 (m, 3H, C_6_H_11_). ^13^C{^1^H} NMR (100.61 MHz, CDCl_3_): δ = 175.1 (s, CO), 154.8 (s, C, C_6_H_4_), 146.3 (s, C, C_6_H_4_), 129.4 (s, CH, C_6_H_4_), 129.0 (s, CH, C_6_H_4_), 120.4 (s, CH, C_6_H_4_), 112.4 (s, CH, C_6_H_4_), 74.9 (s, OCH), 56.1 (s, CH), 52.2 (s, OCH_3_), 31.6 (s, CH_2_), 25.6 (s, CH_2_), 23.48 (s, CH_2_).

#### 3.4.10. Synthesis of Compound (**7**)

Acetylacetone (25.2 μL, 0.245 mmol) was added to a solution of compound **1a** (75.0 mg, 0.123 mmol) in *N*,*N*-dimethylformamide (5 mL). The resulting solution was heated at 100 °C for 2 h. Once cooled, the reaction mixture was filtered over celite to eliminate any remaining solids, washing the filter with ethyl acetate (10 mL). The resulting solution was evaporated to dryness under vacuum. The residue was dissolved in dichloromethane (10 mL) and washed with water (5 × 5 mL). The organic phase was dried over magnesium sulfate, filtered, and evaporated. Further purification was required, using a silica gel column chromatography with dichloromethane as eluent, to yield compound **7** as a yellow solid. Obtained: 20.2 mg, 0.055 mmol, 22% yield. Elemental analysis: Calc. for C_14_H_15_NO_4_Pd (367.70): C, 45.73; H, 4.11; N, 3.81. Exp.: C, 45.68; H, 4.15; N, 3.83. Mass spect. (ESI+) [*m*/*z*]: 308.8 [M-(COOMe)+H]^+^. IR (ν, cm^−1^): 3290 (νN-H), 1717 (νC=O), 1571, 1540 (νC$\dddot{-}$O, acac). ^1^H NMR (300.13 MHz, CDCl_3_): δ = 9.85 (s, 1H, NH), 7.95 (dd, *J* = 7.6, 1.1 Hz, 1H, C_6_H_4_), 7.63 (dd, *J* = 7.7, 0.8 Hz, 1H, C_6_H_4_), 7.30 (td, *J* = 7.5, 1.1 Hz, 1H, C_6_H_4_), 7.09 (td, *J* = 7.5, 0.8 Hz, 1H, C_6_H_4_), 5.43 (s, 1H, CH, acac), 4.01 (s, 3H, OCH_3_), 2.11 (s, 3H, CH_3_, acac), 2.04 (s, 3H, CH_3_, acac). ^13^C{^1^H} NMR (75.47 MHz, CDCl_3_): δ = 188.4 (s, CO, acac), 187.0 (s, CO, acac), 172.1 (s, CO_2_), 159.7, 159.3 (2s, C=N + Pd-C), 142.7 (s, Cq, C_6_H_4_), 132.2 (s, CH, C_6_H_4_), 131.3 (s, CH, C_6_H_4_), 130.5 (s, CH, C_6_H_4_), 124.7 (s, CH, C_6_H_4_), 100.9 (s, CH, acac), 53.7 (s, OCH_3_), 27.9 (s, CH_3_, acac), 27.8 (s, CH_3_, acac).

## 4. Conclusions

In summary, it is possible to achieve the functionalization of orthopalladated phenylglycines through oxidative coupling reactions in two simple steps. In the first step, the actual functionalization takes place, and the modified phenylglycine remains bound to Pd. In the second step, the phenylglycine ligand is released by displacing Pd with 1,10-phenanthroline. This approach enables selective monohalogenation and monoalkoxylation at the ortho position of the aromatic ring in various types of phenylglycines substituted on the aromatic ring, the nitrogen atom, and the α-carbon. Both halogenation and alkoxylation are general processes and tolerate the presence of different substituents. In the case of alkoxylation, it has been confirmed that the reactions preserve a high percentage of the precursor’s chirality, although losses in enantiomeric excess have been observed. These losses are associated with the acidity of the hydrogen on the α-carbon of the phenylglycine and its tendency to degrade into an imine. The presence of imine and azavinylidene species as Pd ligands in the degradation processes of phenylglycine has been demonstrated and characterized. These findings do not undermine the validity of the method but rather highlight key aspects to consider when functionalizing this type of substrate.

## Data Availability

Data are contained within the article and Supplementary Materials.

## Supplementary Materials

The following supporting information can be downloaded at: https://www.mdpi.com/article/10.3390/molecules30020236/s1; Table S1: Optimization of the reaction conditions for the alkoxylation of phenyglycines; copies of ^1^H, ^13^C and ^19^F NMR spectra of all new species; Table S2: Crystal data and structure refinement for **6**; Table S3: Selected bond distances (Å) and angles (°) for **6**; Table S4: Crystal data and structure refinement for **7**; Table S5: Selected bond distances (Å) and angles (°) for **7**.
