# Supplementary material for: o-Halogenation and -Alkoxylation of Phenylglycine Derivatives by Pd-Mediated C-H Functionalization: Scope and Limitations
Source: Molecules. 2025 Jan 9;30(2):236. doi: 10.3390/molecules30020236 (PMC11767792; doi:10.3390/molecules30020236)
Supplement: Supplementary file 1 [file molecules-30-00236-s001.zip › molecules-3392698-supplementary.pdf]

Ortho-halogenation and -alkoxylation of phenylglycine derivatives by Pd-mediated C-H functionalization: scope and limitations

Eduardo Laga, Sonia Nieto, Carlos Cativiela, Esteban P. Urriolabeitia*

Instituto de Síntesis Química y Catálisis Homogénea, ISQCH (CSIC – Universidad de Zaragoza), Pedro Cerbuna 12, 50009 Zaragoza, Spain

E-mail: esteban.u.a@csic.es; esteban@unizar.es.

Supplementary Material

Table S1 **S2**

NMR spectra of all new species prepared in this work **S3**

Crystallographic Tables **S29**

Table S1. Optimization of the reaction conditions for the alkoxylation of phenyglycines[a]

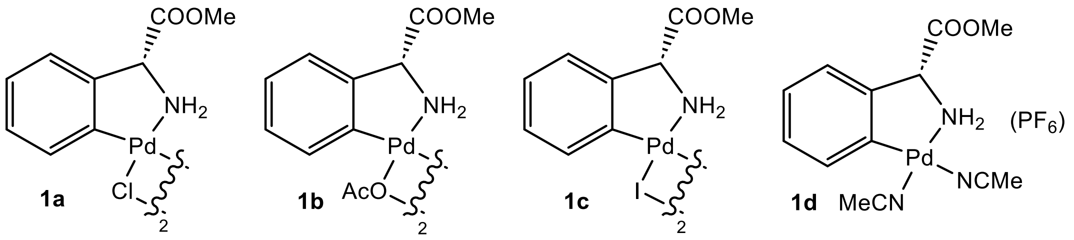

Entry	Pd source	Alcohol	Oxidant	T	Yield (%)	ee (%)
1	**1a**	MeOH	$PhI(OAc)_2$	25	72	96
2	**1b**	MeOH	$PhI(OAc)_2$	25	72	70
3	**1c**	MeOH	$PhI(OAc)_2$	25	16	-
4	**1d**	MeOH	$PhI(OAc)_2$	25	-	-
5	**1a**	EtOH	$PhI(OAc)_2$	25	38	96
6	**1b**	EtOH	$PhI(OAc)_2$	25	-	-
7	**1a**	iPrOH	$PhI(OAc)_2$	25	42	94
8	**1b**	iPrOH	$PhI(OAc)_2$	25	-	-
9	**1a**	MeOH	$Cu(OAc)_2$	25	-	
10	**1a**	MeOH	benzoquinone	25	-	
11	**1a**	MeOH	H_2O_2	25	-	
12	**1a**	MeOH	Oxone	25	-	
13	**1a**	MeOH	$PhI(OAc)_2$	100	30	-

a) General conditions: **1x (x = a, b, c, d)** (0.3 mmol) reacted with oxidant (1.2 mmol) in alcohol (10 mL) at room temperature (except entry 13, T = 100 ºC) for 20h. The intermediate PdCl2L2 was isolated, purified and treated with 1,10-phen (0.3 mmol) to give **5a**, **5b** or **5c** depending of the alcohol.

NMR spectra of all new species prepared in this work

Methyl 2’-(bromo)-6’-(iodo)phenylglycinate (3bc)

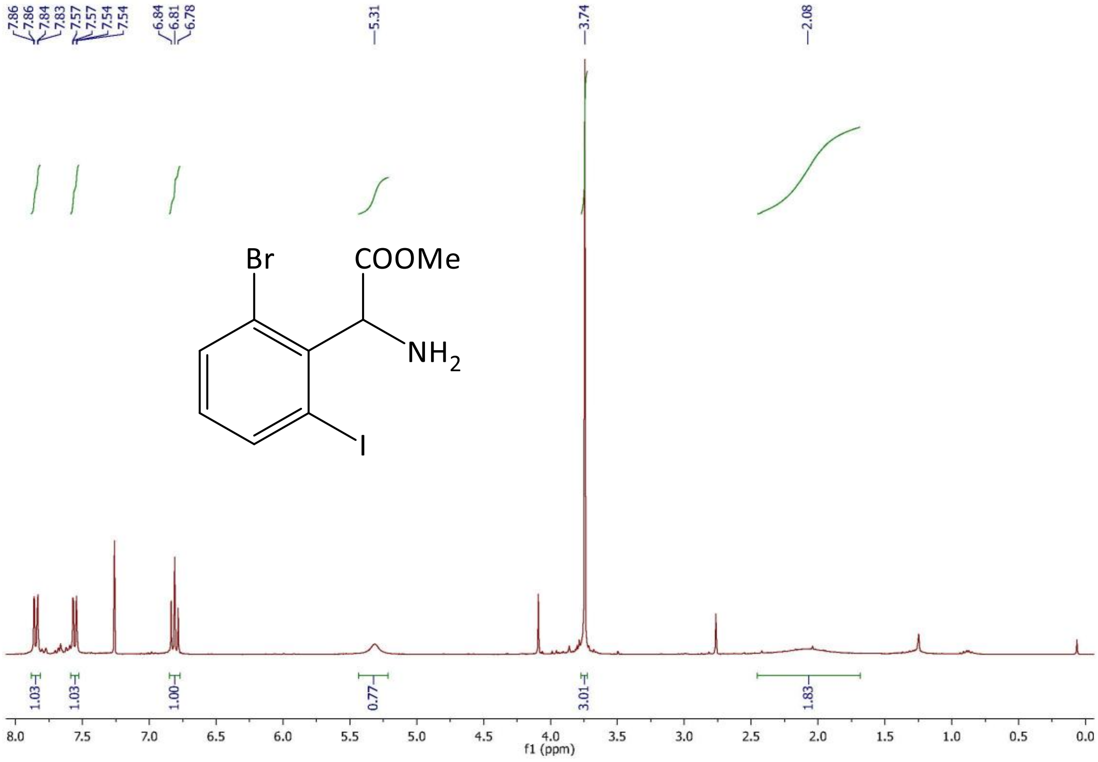

^{1}H NMR ($CDCl_3$, 300.13 MHz, 298K) of 3bc

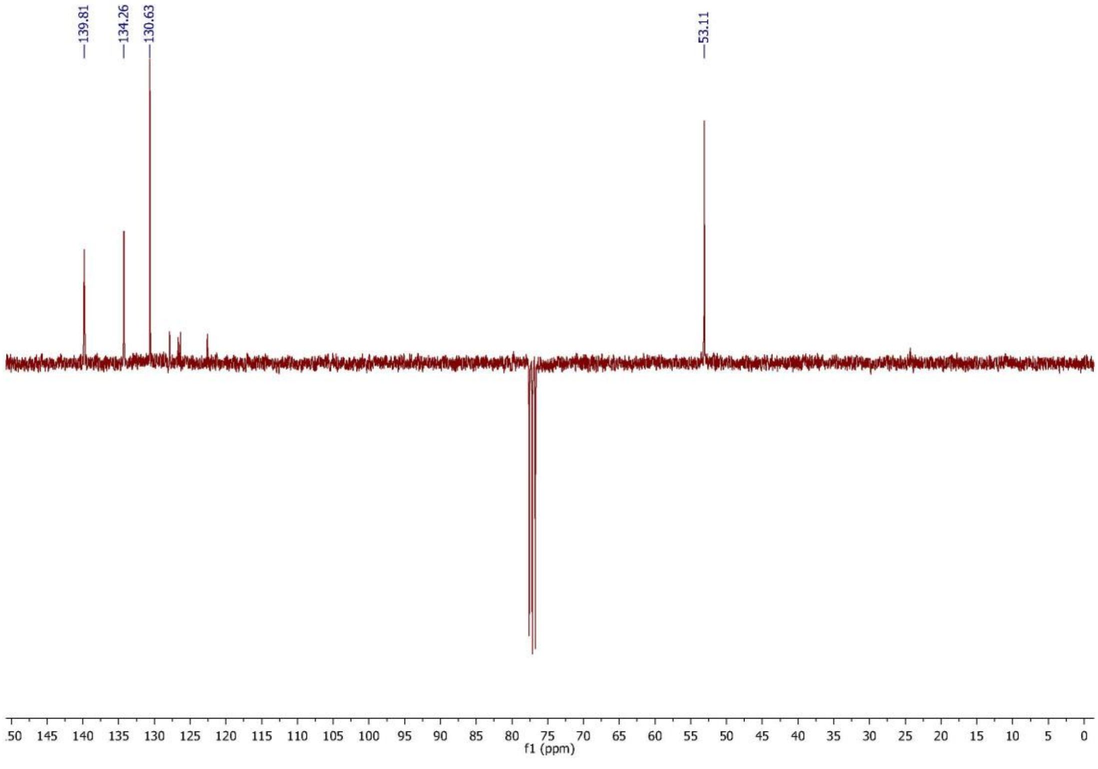

$^{13}C\{^{1}H\}$ NMR (APT, $CDCl_3$, 75.47 MHz, 298K) of 3bc

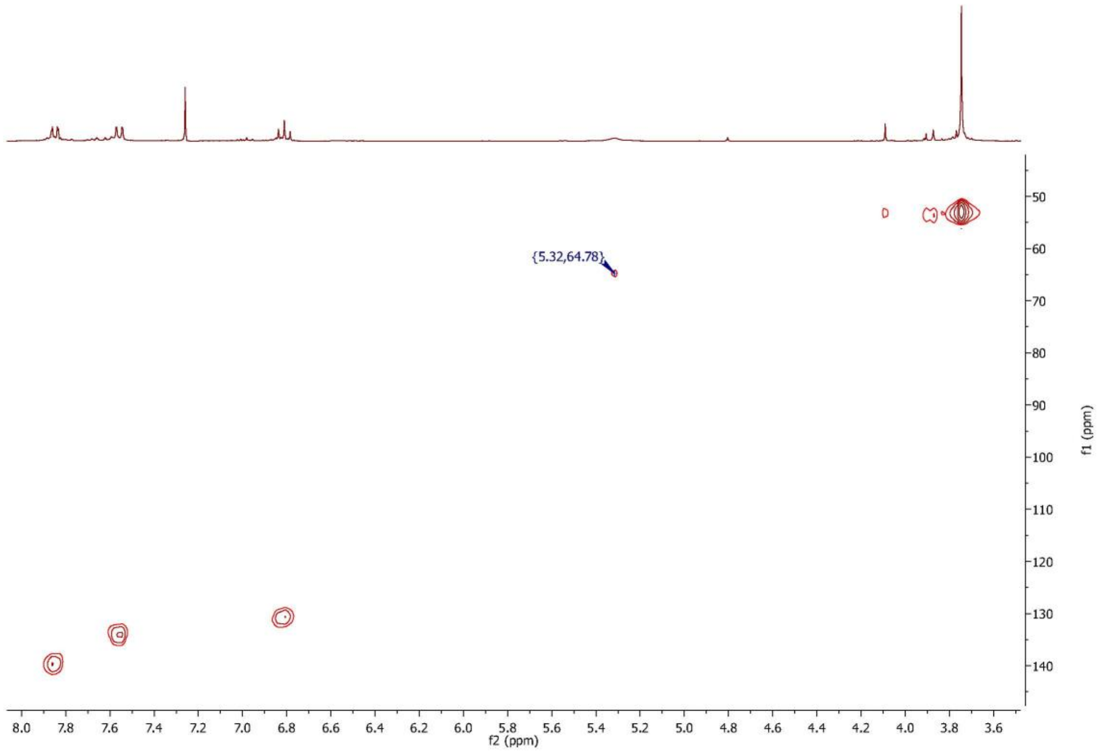

^{1}H-^{13}C HSQC correlation ($CDCl_3$, 298K)

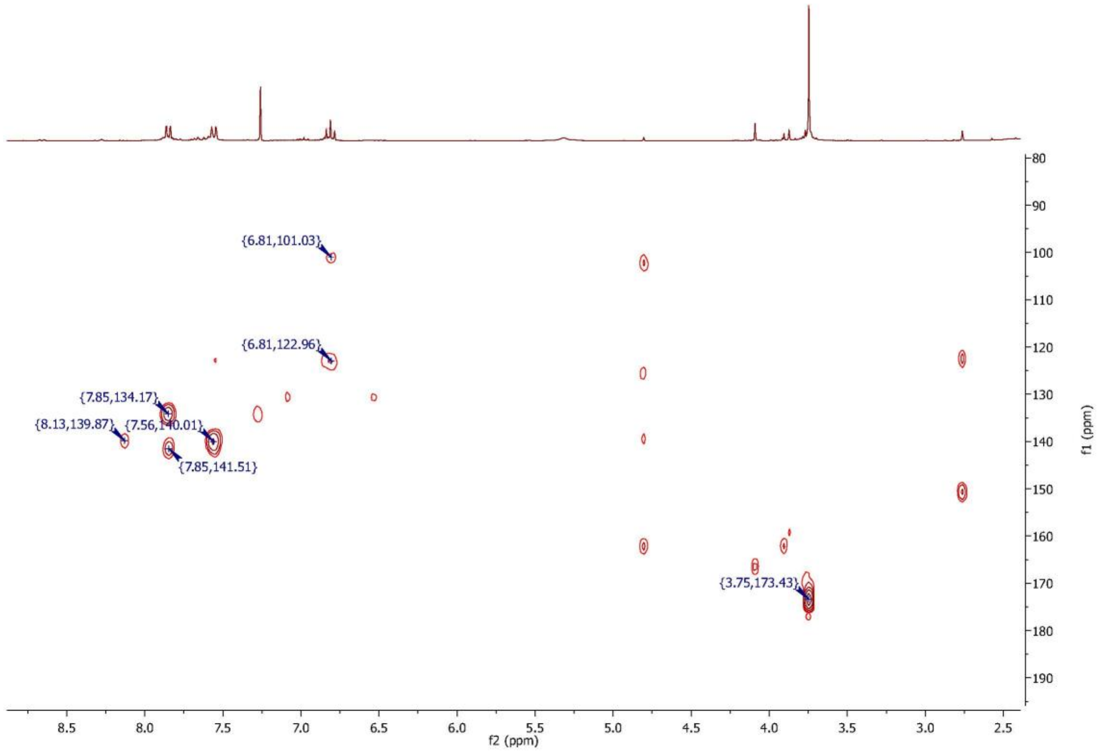

^{1}H-^{13}C HMBC correlation ($CDCl_3$, 298K)

Methyl 5'-(bromo)-2'-(iodo)phenylglycinate (3cc)

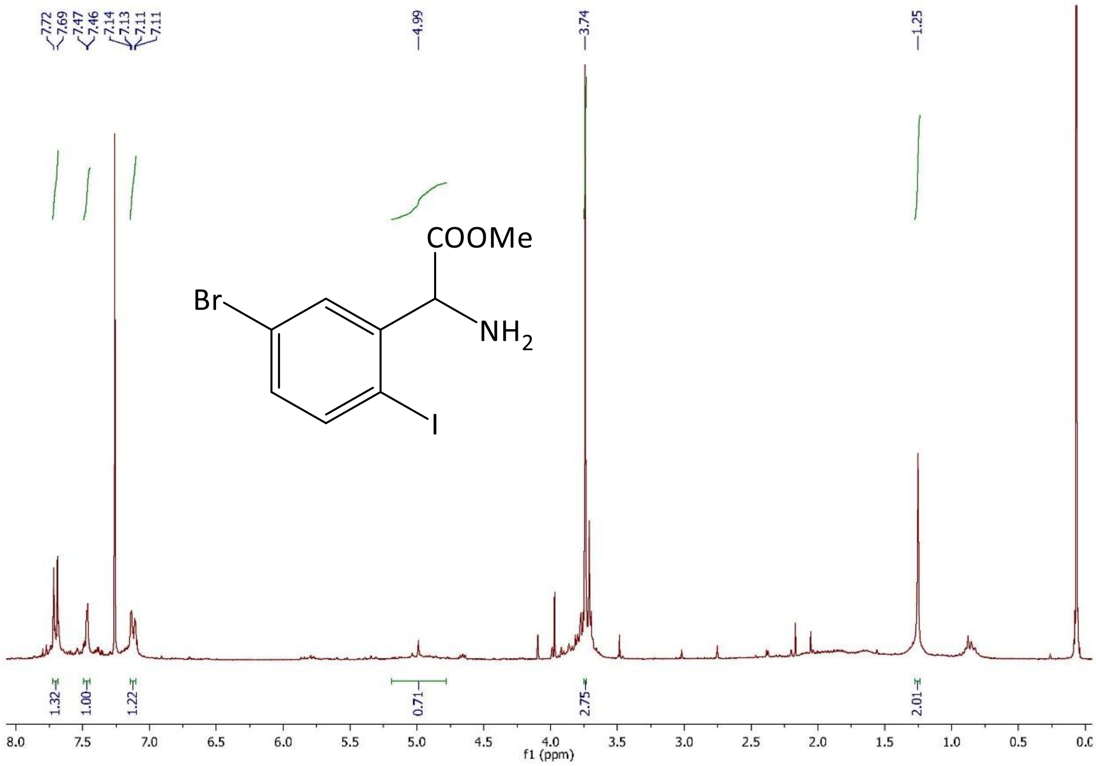

^{1}H NMR ($CDCl_3$, 300.13 MHz, 298K) of 3cc

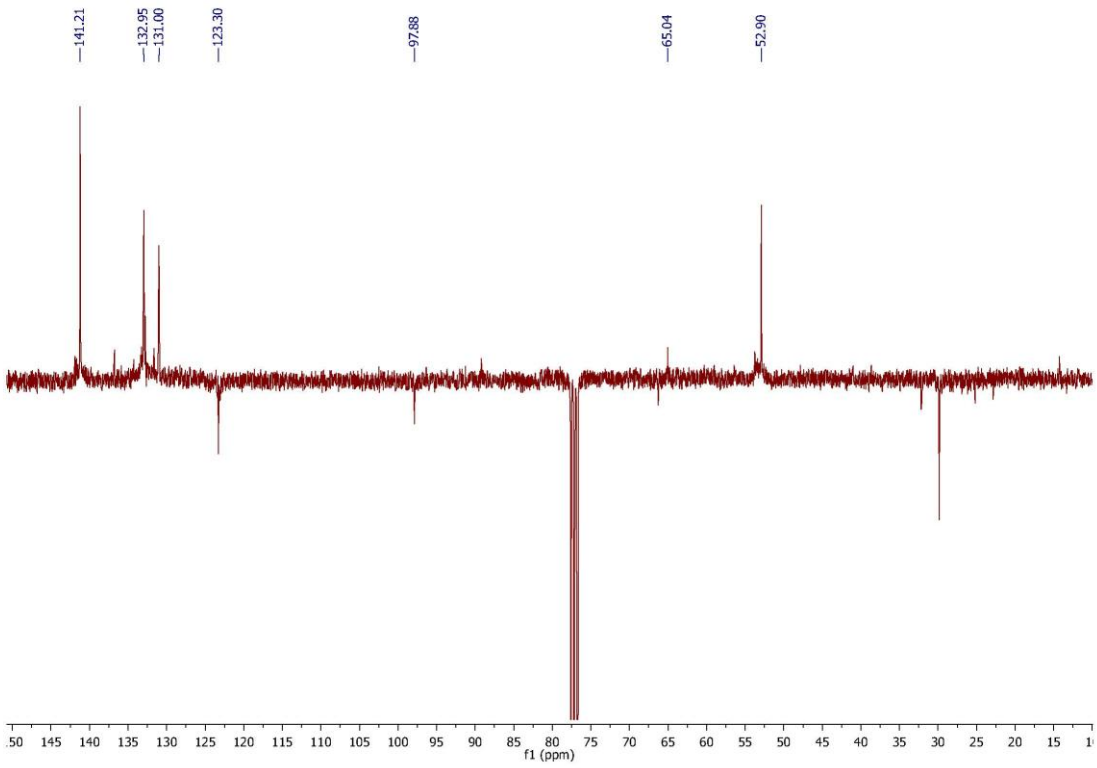

$^{13}C\{^1H\}$ NMR (APT, $CDCl_3$, 75.47 MHz, 298K) of 3cc

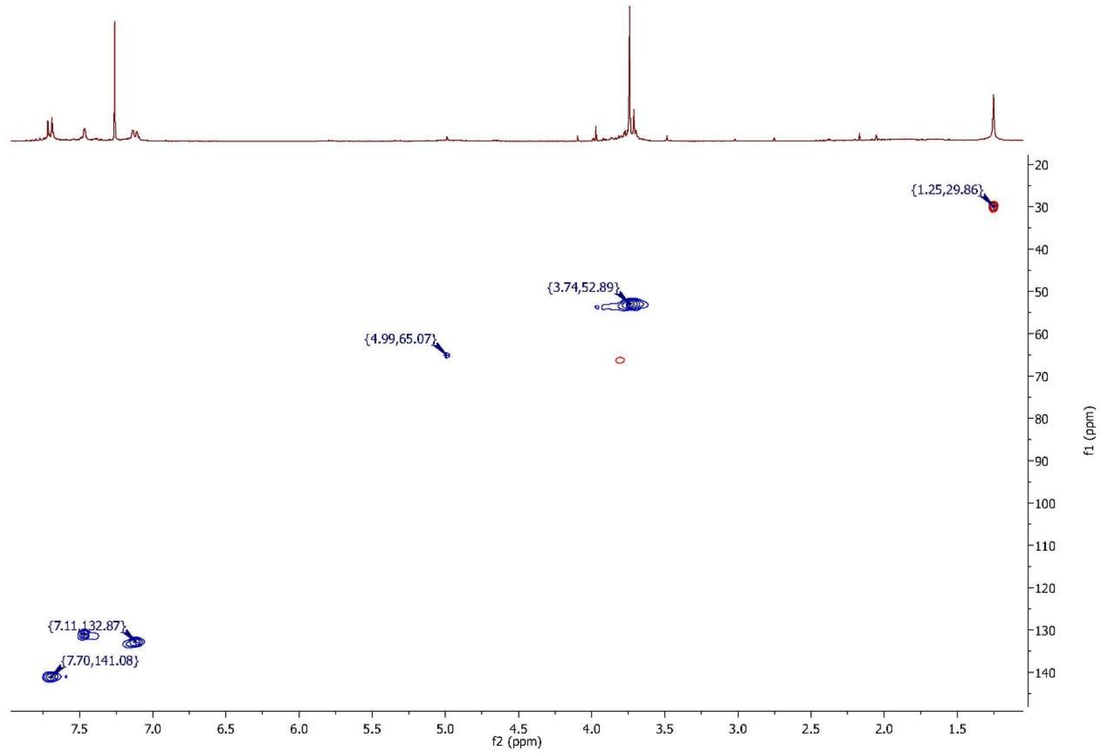

^1H-^{13}C HSQC correlation ($CDCl_3$, 298K)

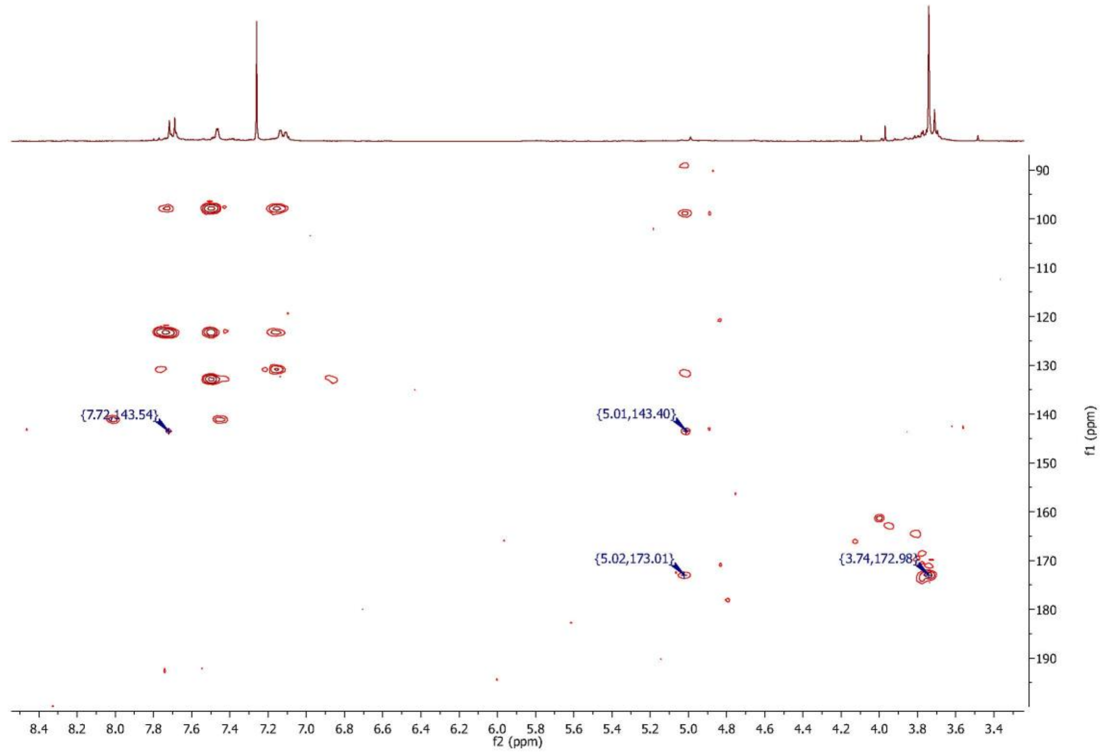

^1H-^{13}C HMBC correlation ($CDCl_3$, 298K)

Methyl 4’-(bromo)-2’-(iodo)-phenylglycinate (3dc)

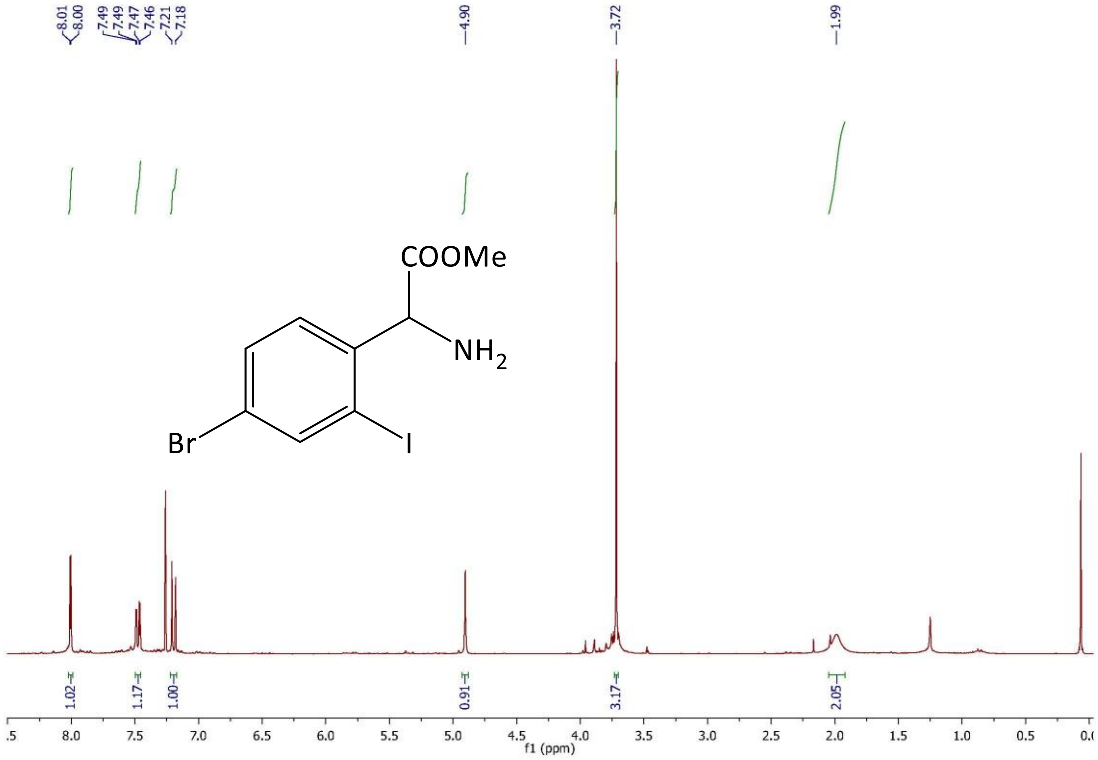

^{1}H NMR ($CDCl_3$, 300.13 MHz, 298K) of 3dc

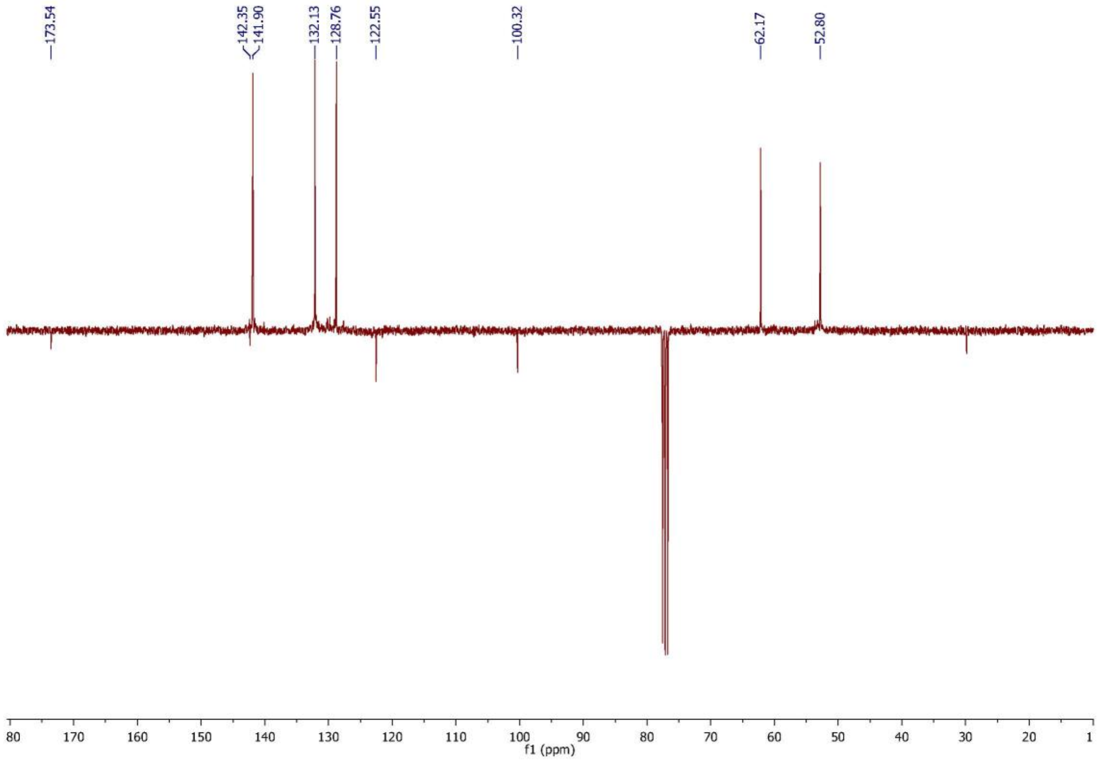

^{13}C{^{1}H} NMR (APT, $CDCl_3$, 75.47 MHz, 298K) of 3dc

Methyl *N*,*N*-dimethyl-2'-(iodo)phenylglycinate (3ec)

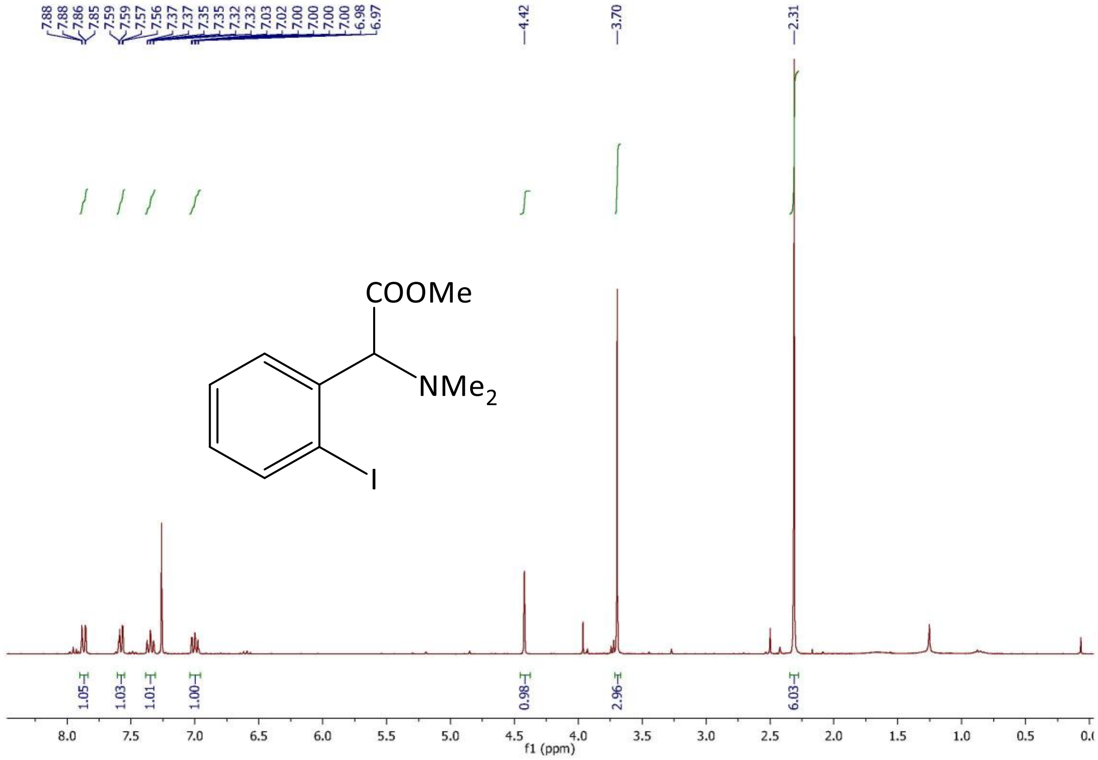

^{1}H NMR (CDCl$_3$, 300.13 MHz, 298K) of 3ec

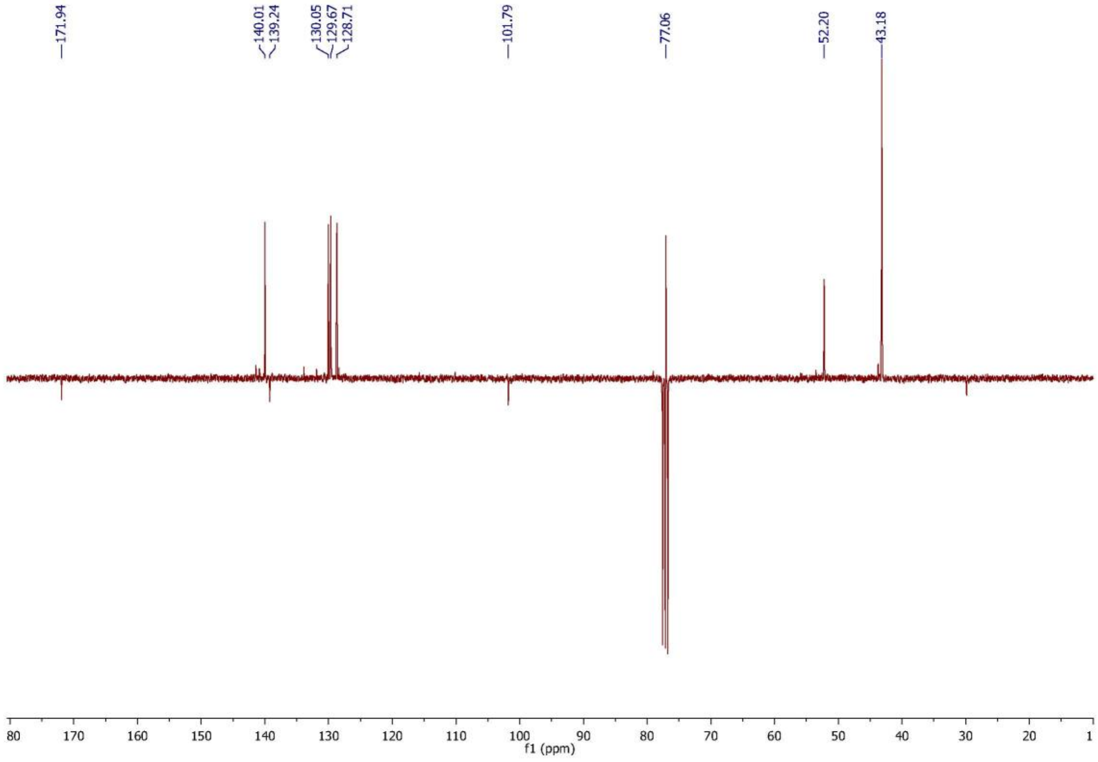

^{13}C{^{1}H} NMR (APT, CDCl$_3$, 75.47 MHz, 298K) of 3ec

Methyl α-methyl-2'-(chloro)phenylglycinate (3fa)

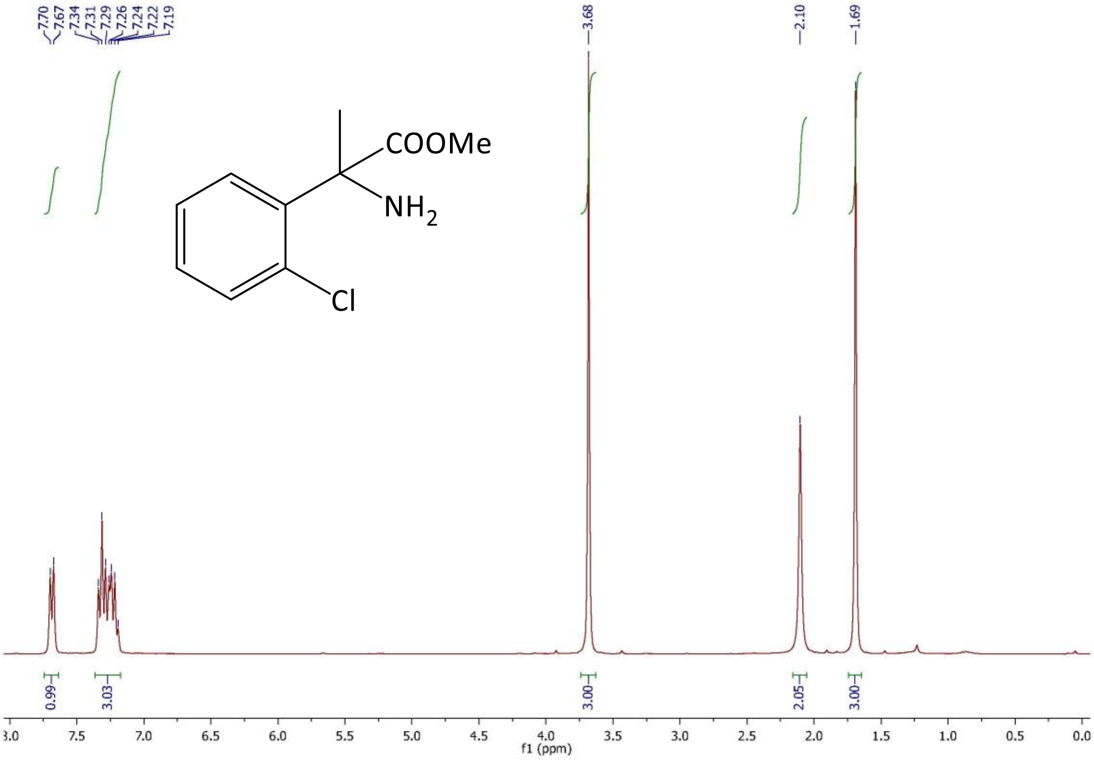

^{1}H NMR ($CDCl_3$, 300.13 MHz, 298K) of 3fa

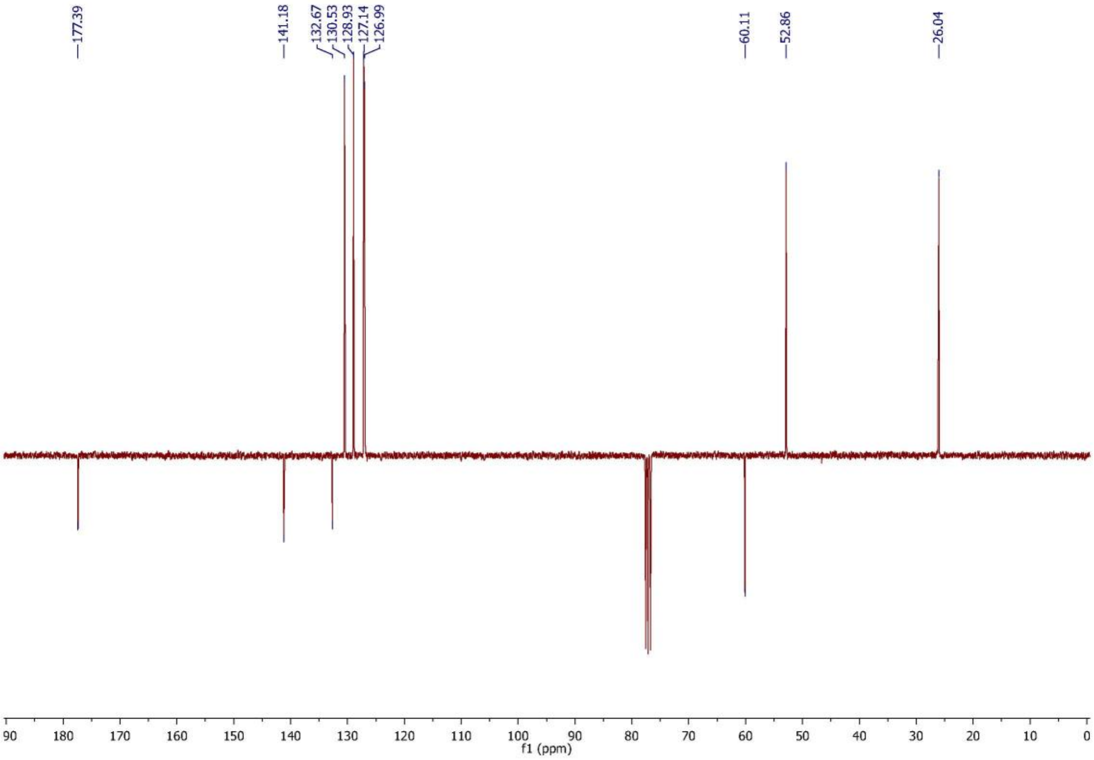

$^{13}C\{^{1}H\}$ NMR (APT, $CDCl_3$, 75.47 MHz, 298K) of 3fa

Methyl α-methyl-2’-(bromo)phenylglycinate (3fb)

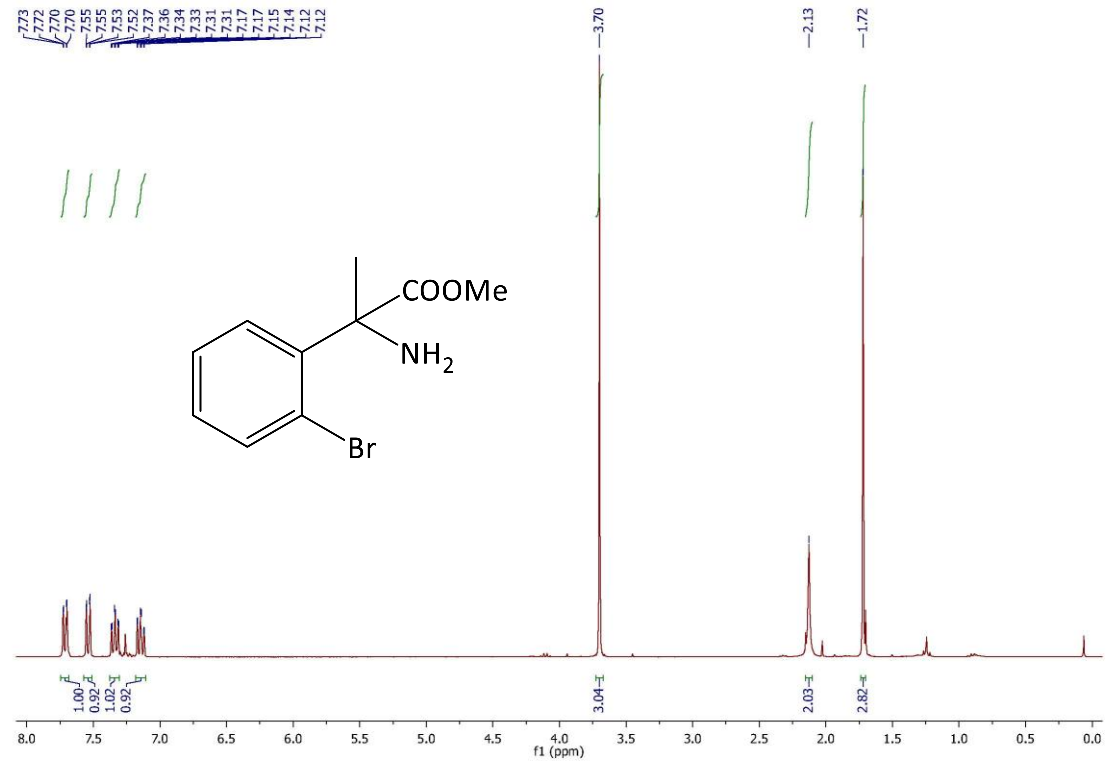

^{1}H NMR ($CDCl_3$, 300.13 MHz, 298K) of 3fb

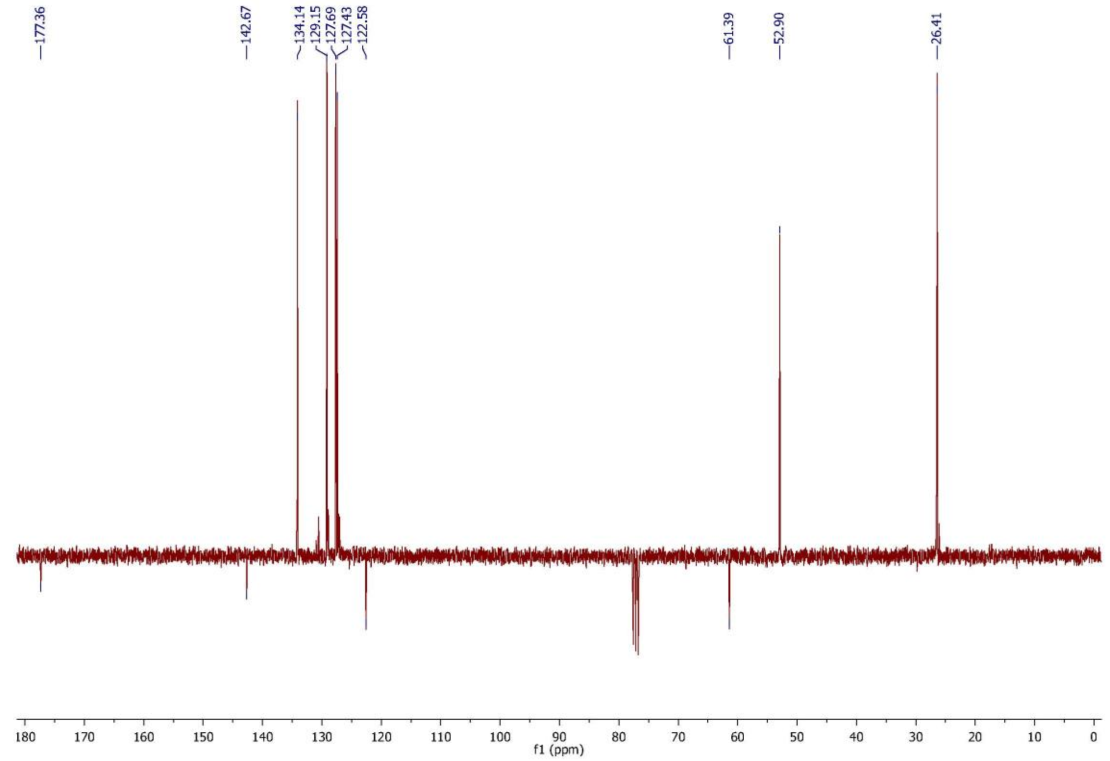

$^{13}C\{^1H\}$ NMR (APT, $CDCl_3$, 75.47 MHz, 298K) of 3fb

Methyl α-methyl-2'-(iodo)phenylglycinate (3fc)

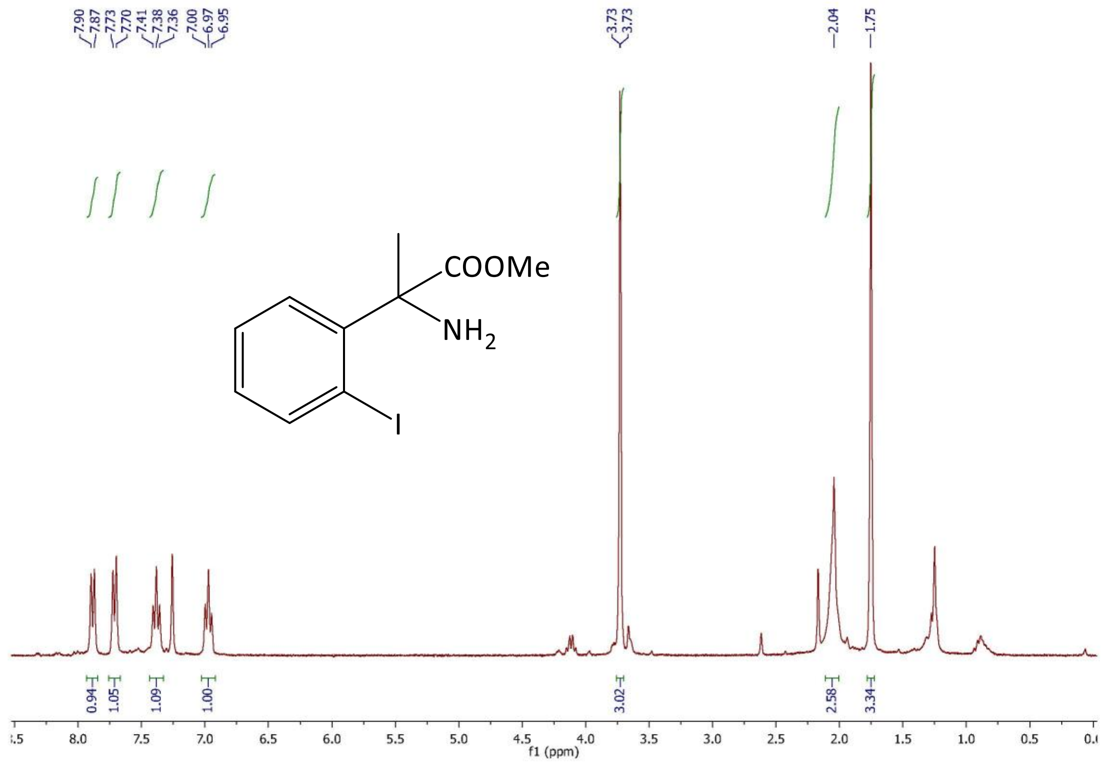

^{1}H NMR ($CDCl_3$, 300.13 MHz, 298K) of 3fc

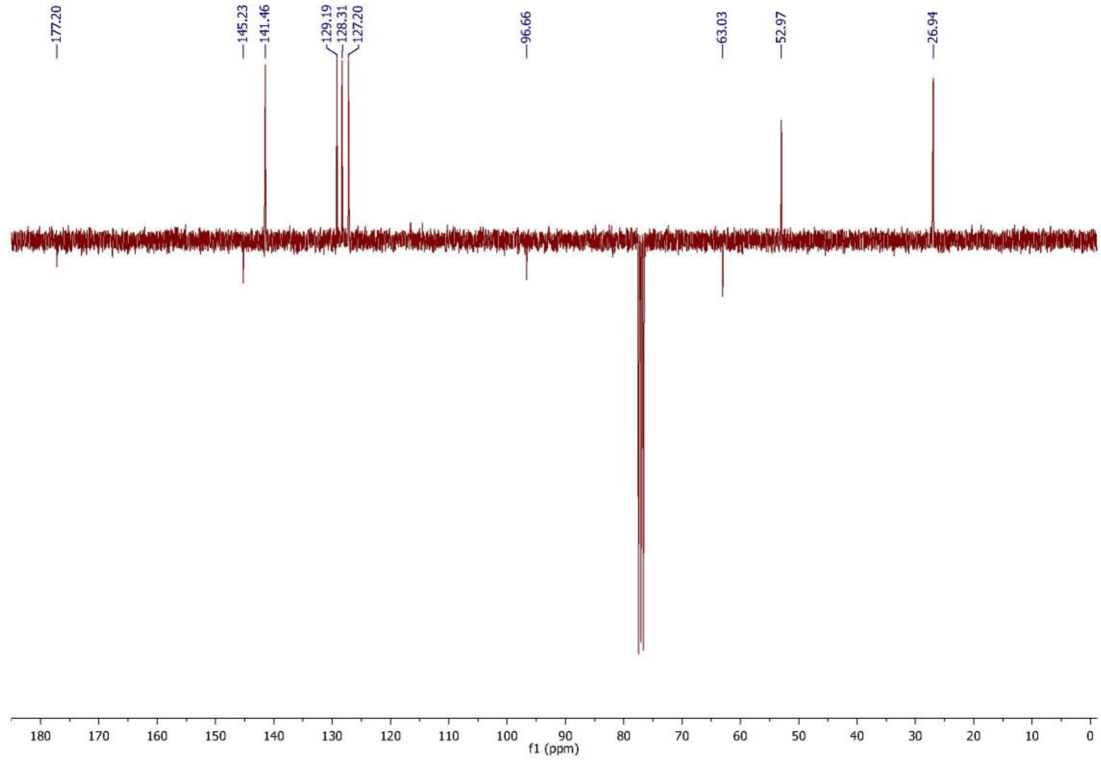

$^{13}C\{^{1}H\}$ NMR (APT, $CDCl_3$, 75.47 MHz, 298K) of 3fc

Methyl α-benzyl-2’-(chloro)phenylglycinate (3ga)

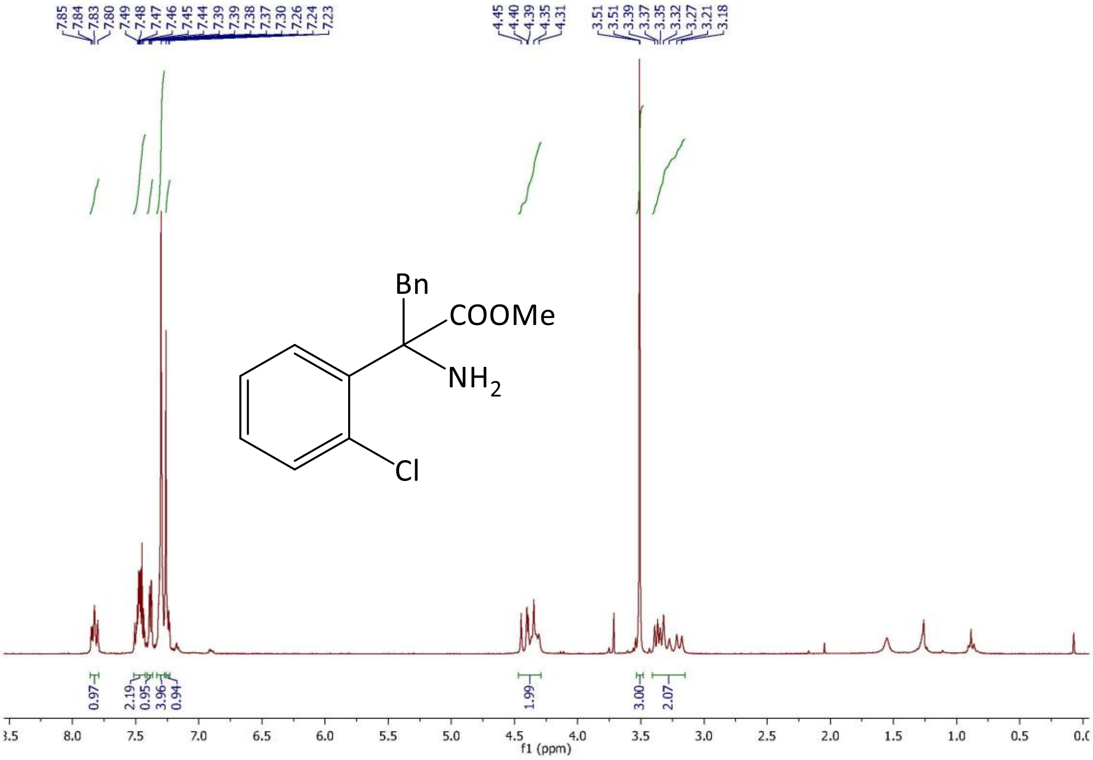

^{1}H NMR (CDCl$_3$, 300.13 MHz, 298K) of 3ga

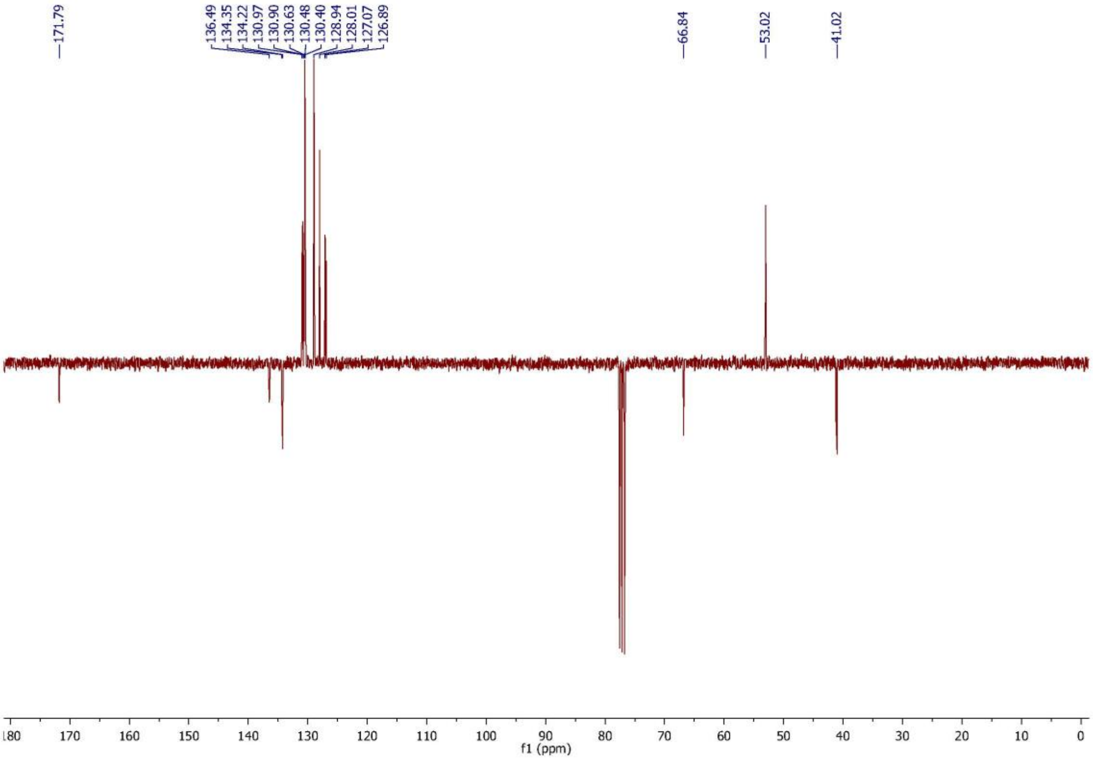

^{13}C{^{1}H} NMR (APT, CDCl$_3$, 75.47 MHz, 298K) of 3ga

Methyl α-benzyl-2'-(bromo)phenylglycinate (3gb)

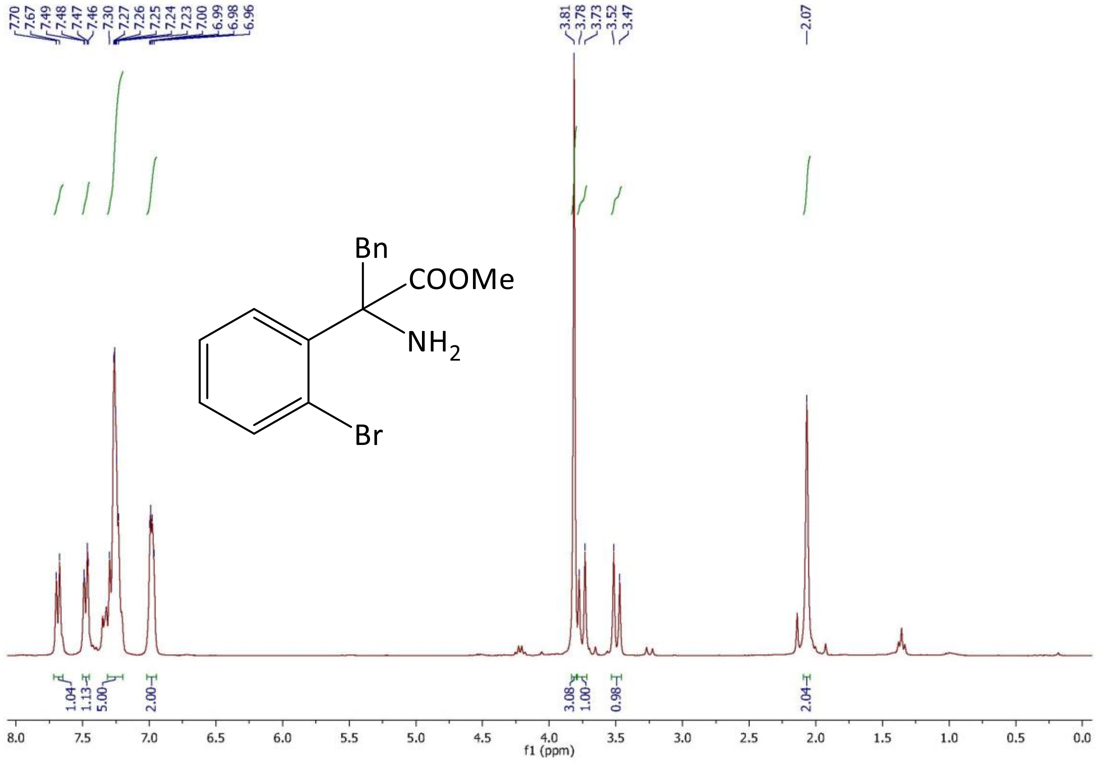

^{1}H NMR (CDCl$_3$, 300.13 MHz, 298K) of 3gb

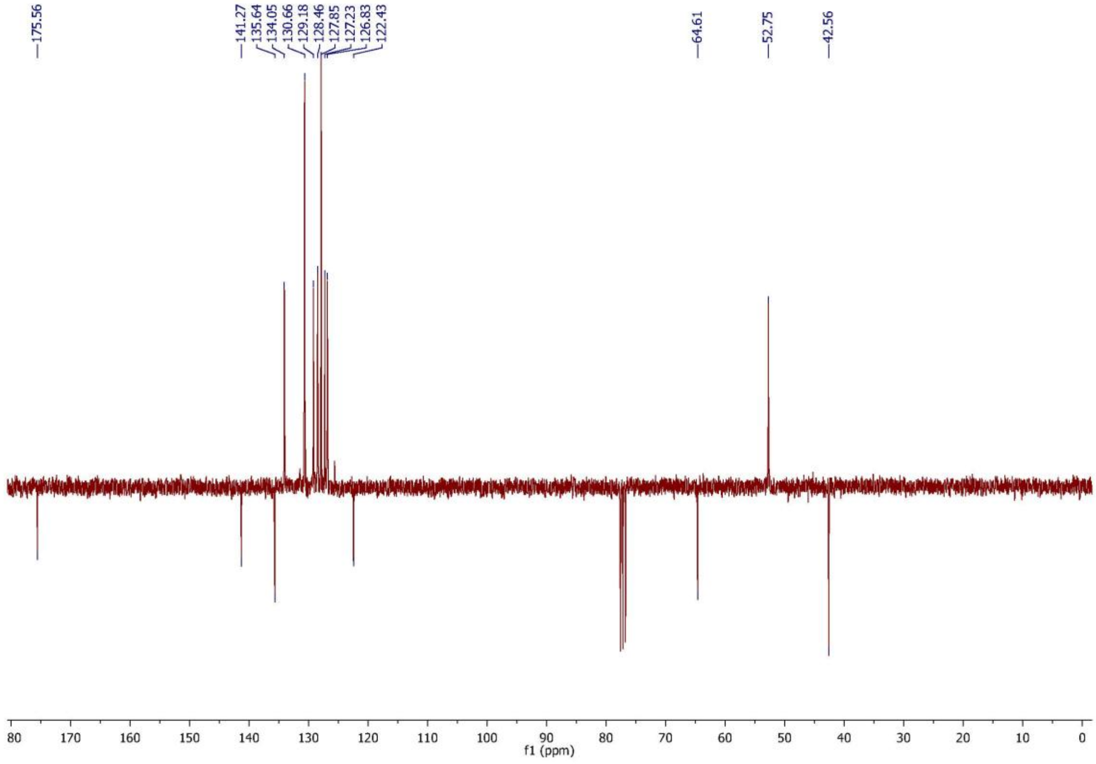

^{13}C{^{1}H} NMR (APT, CDCl$_3$, 75.47 MHz, 298K) of 3gb

Methyl α-benzyl-2’-(iodo)phenylglycinate (3gc)

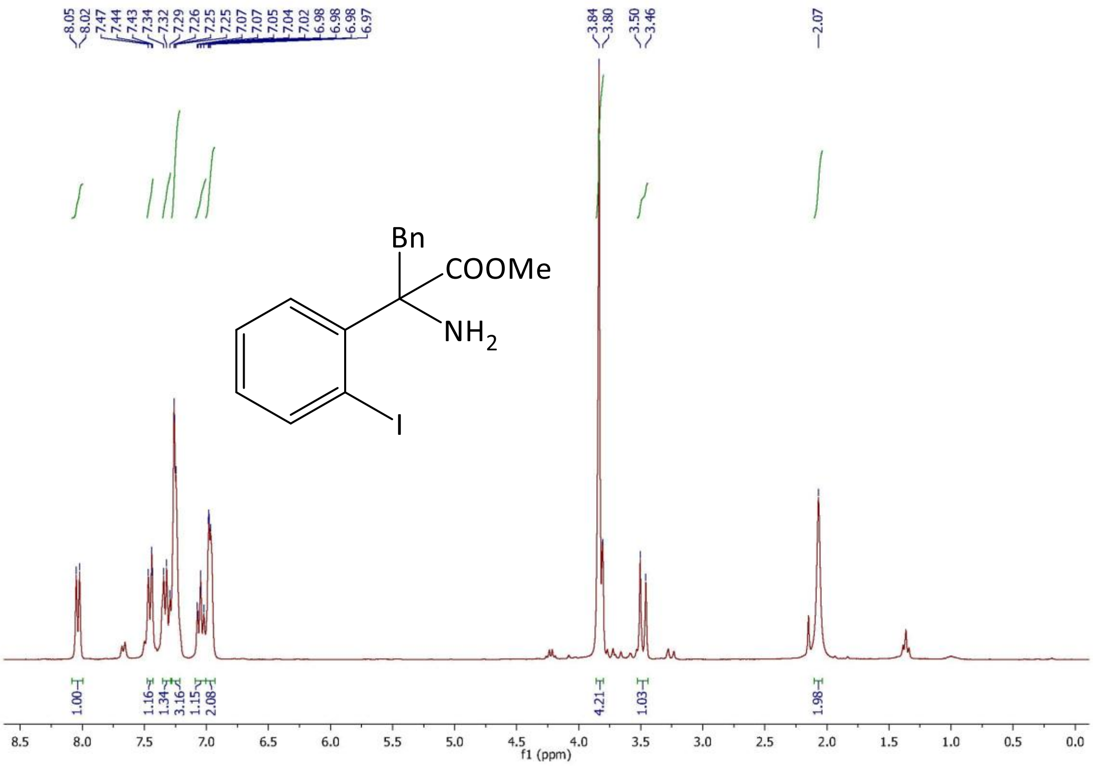

^{1}H NMR ($CDCl_3$, 300.13 MHz, 298K) of 3gc

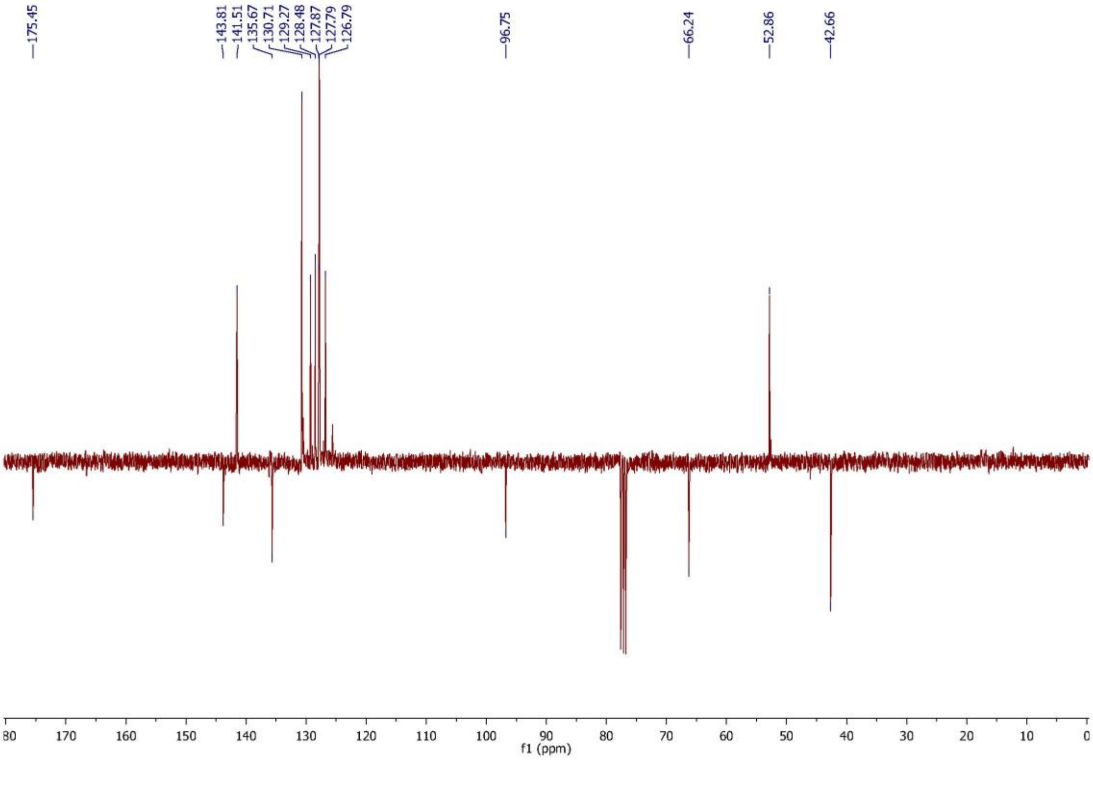

$^{13}C\{^{1}H\}$ NMR (APT, $CDCl_3$, 75.47 MHz, 298K) of 3gc

(*R*)-Methyl 2’-(1-propoxy)phenylglycinate (5aa)

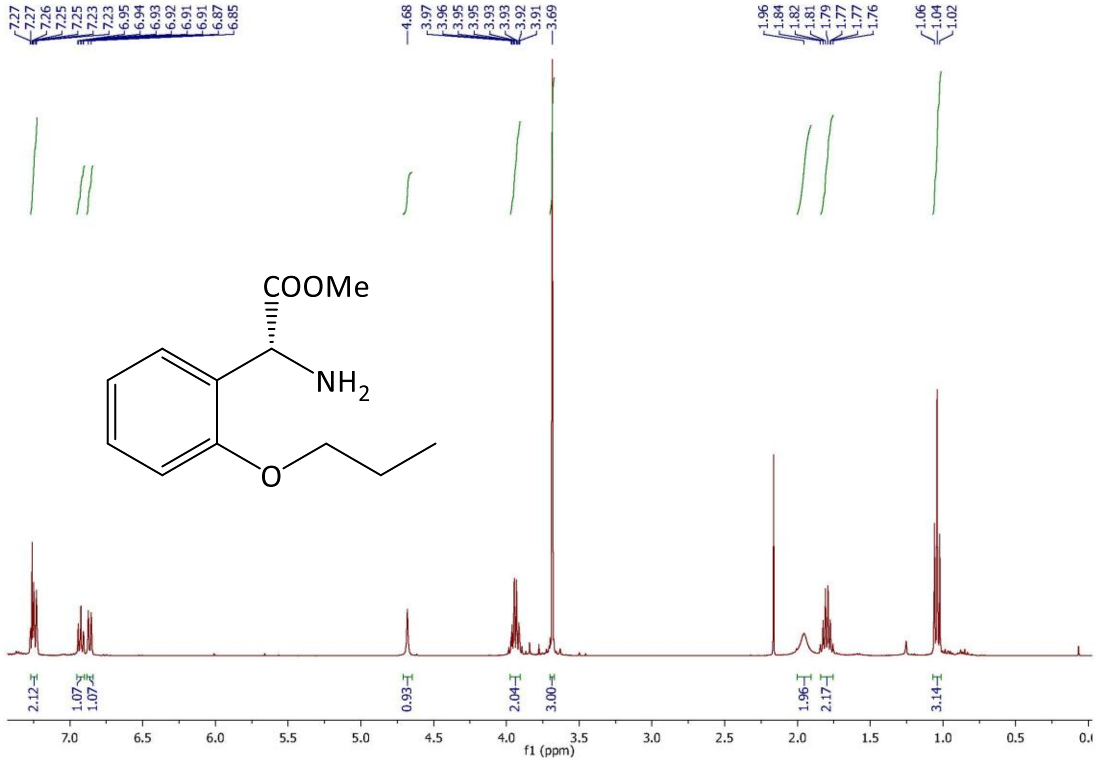

^{1}H NMR ($CDCl_3$, 400.13 MHz, 298K) of 5aa

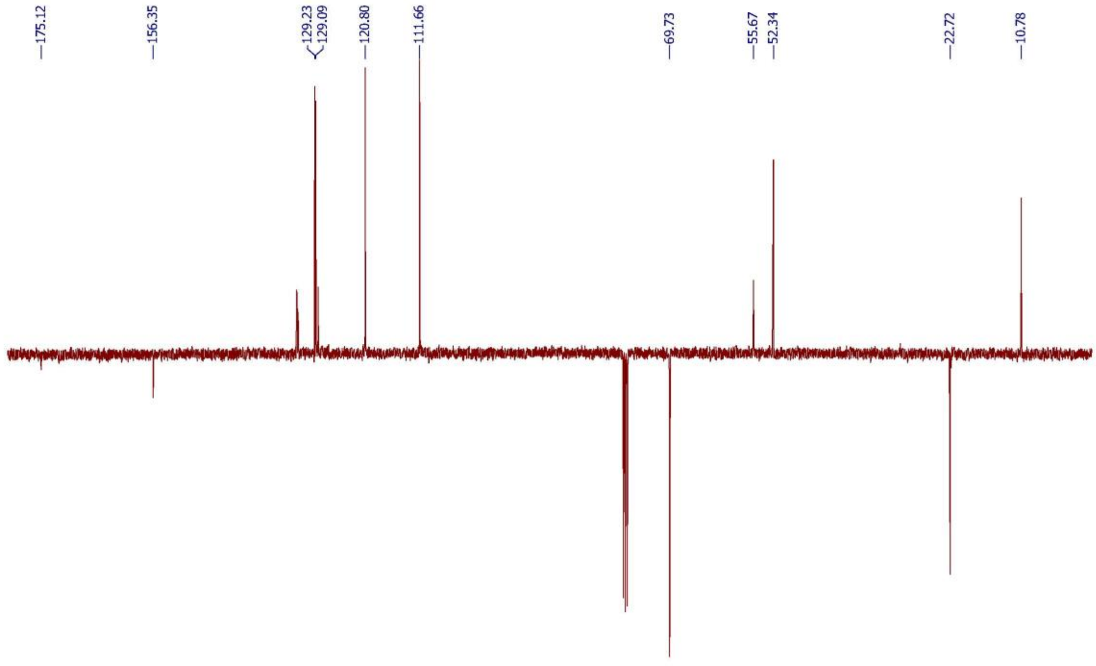

$^{13}C\{^{1}H\}$ NMR (APT, $CDCl_3$, 100.61 MHz, 298K) of 5aa

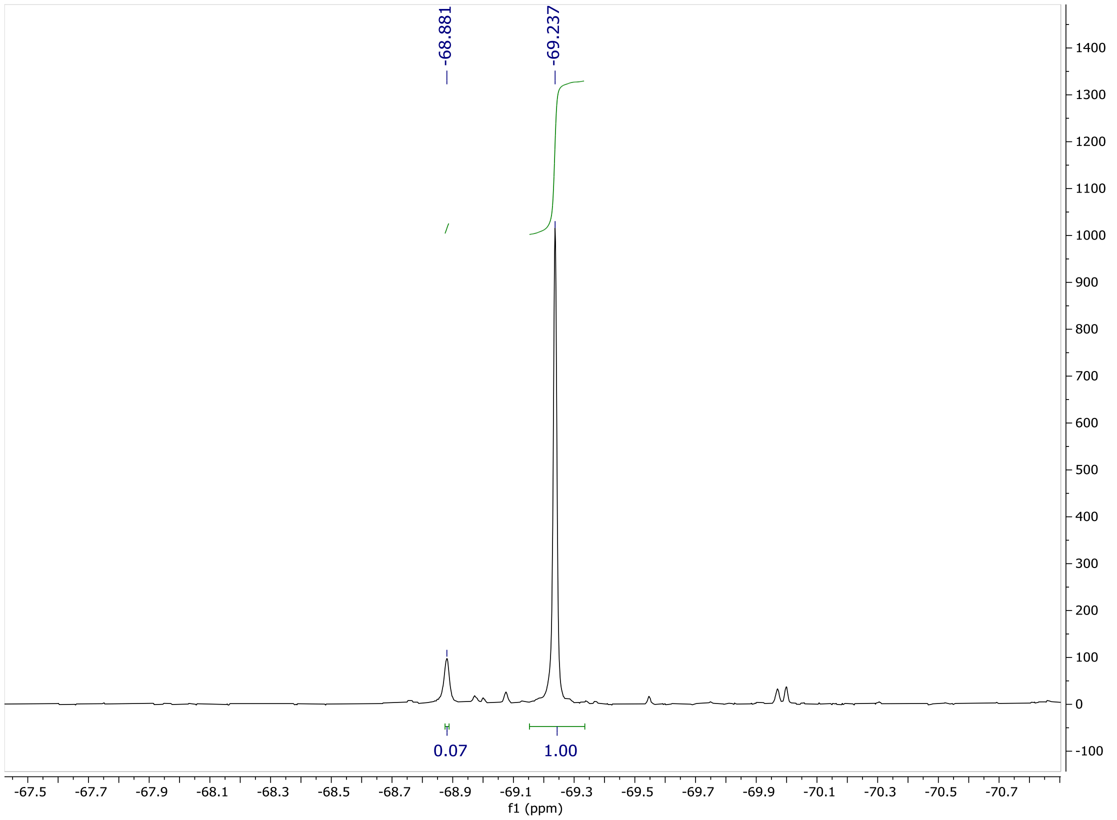

^{19}F NMR ($CDCl_3$, 282.40 MHz, 298K) of 5aa

(*R*)-Methyl 2’-(2-propoxy)phenylglycinate (5ab)

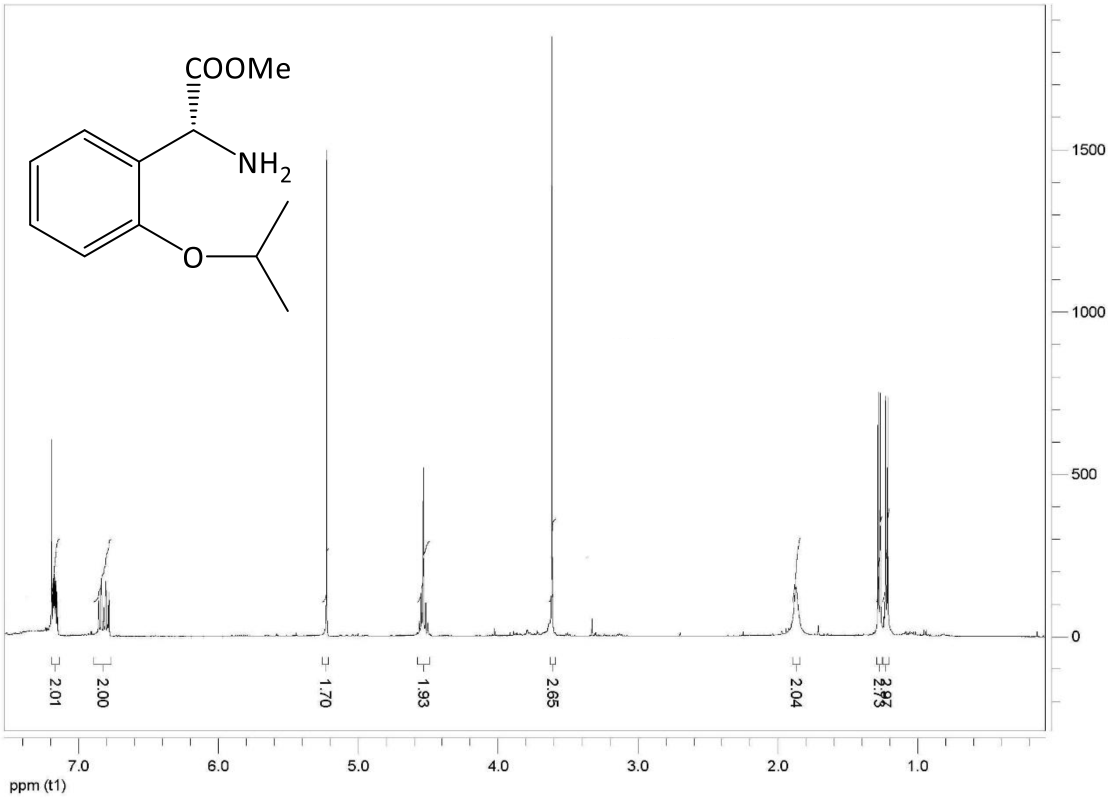

^{1}H NMR ($CDCl_3$, 400.13 MHz, 298K) of 5ab

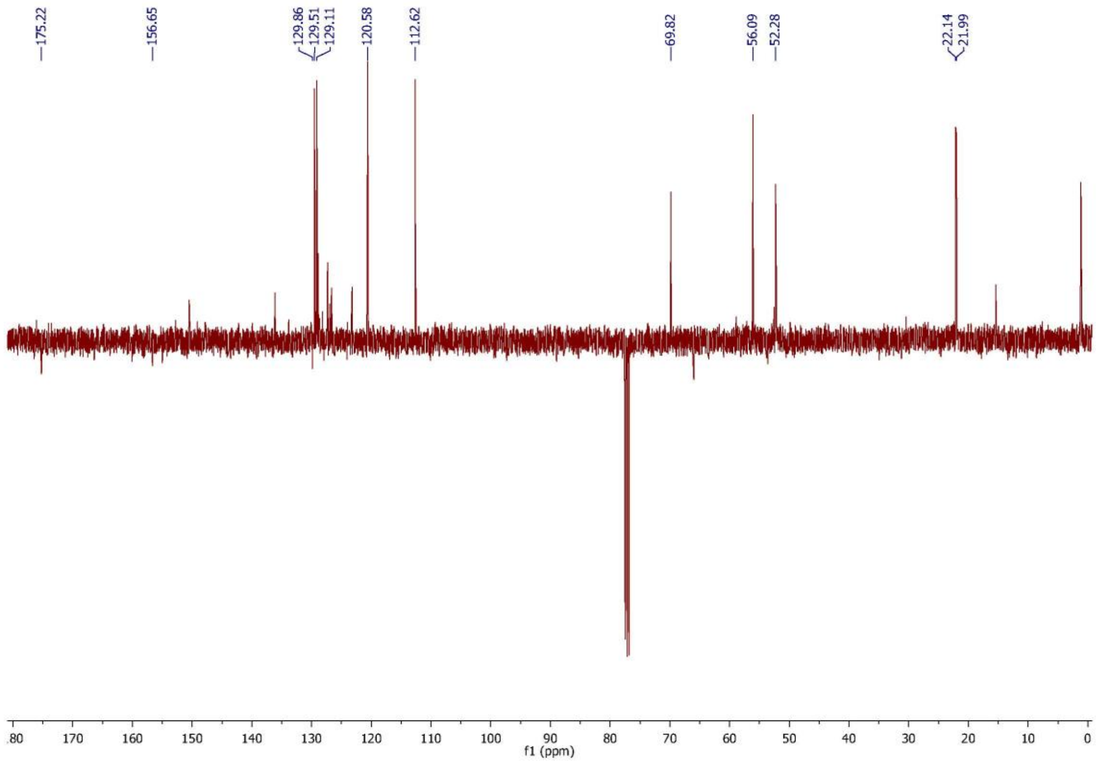

$^{13}C\{^{1}H\}$ **NMR (APT, $CDCl_3$, 100.61 MHz, 298K) of 5ab**

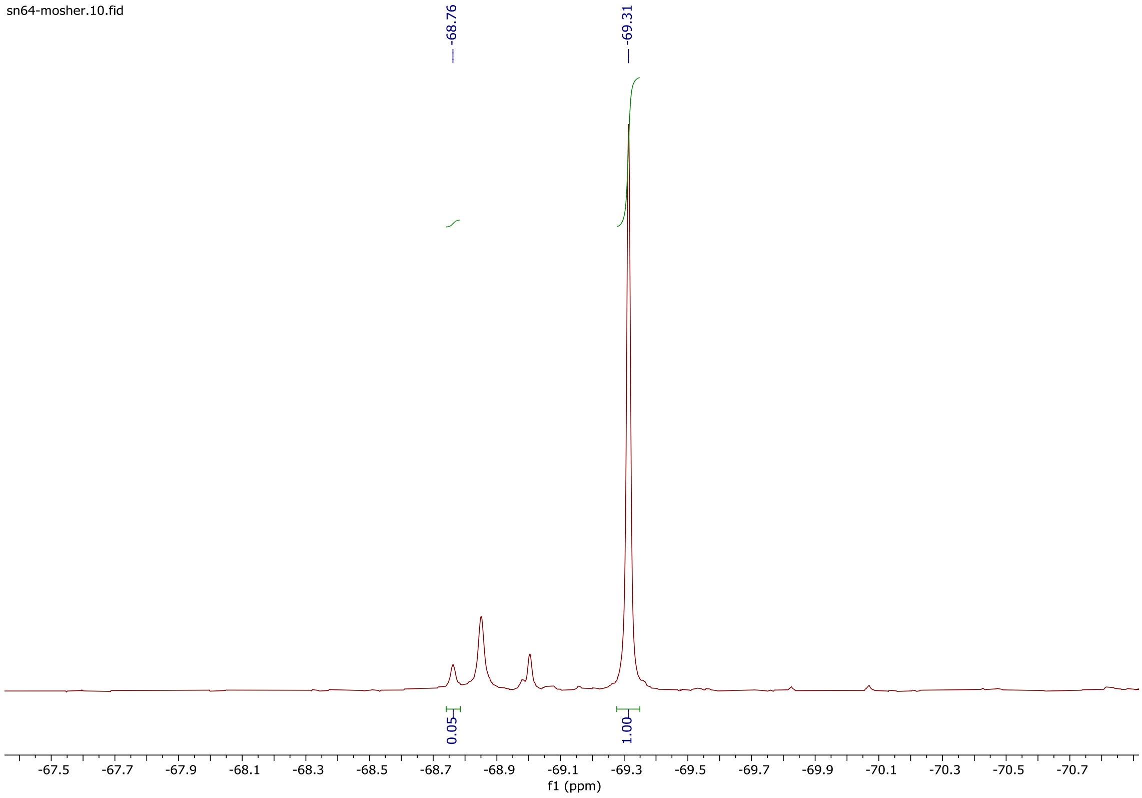

^{19}F **NMR ($CDCl_3$, 282.40 MHz, 298K) of 5ab**

(*R*)-Methyl 2’-(1-butoxy)phenylglycinate (5ac)

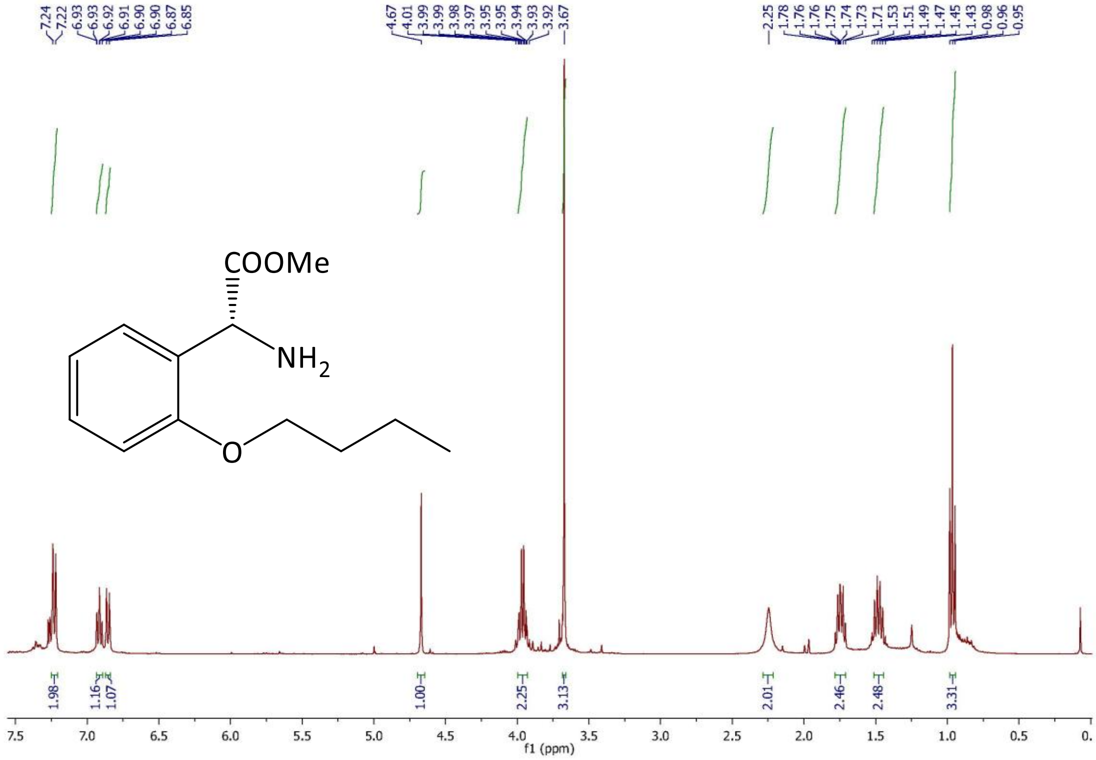

^{1}H NMR ($CDCl_3$, 400.13 MHz, 298K) of 5ac

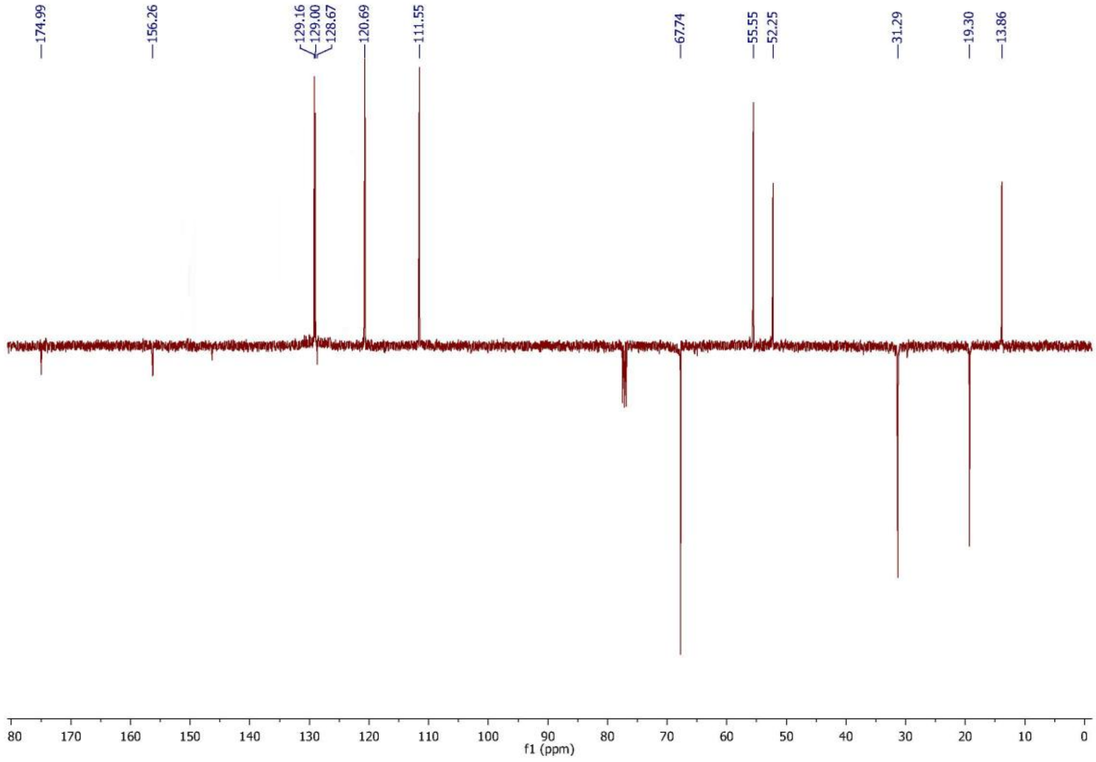

^{13}C{^{1}H} NMR (APT, $CDCl_3$, 100.61 MHz, 298K) of 5ac

(*R*)-Methyl 2'-(2-methyl-1-propoxy)phenylglycinate (5ad)

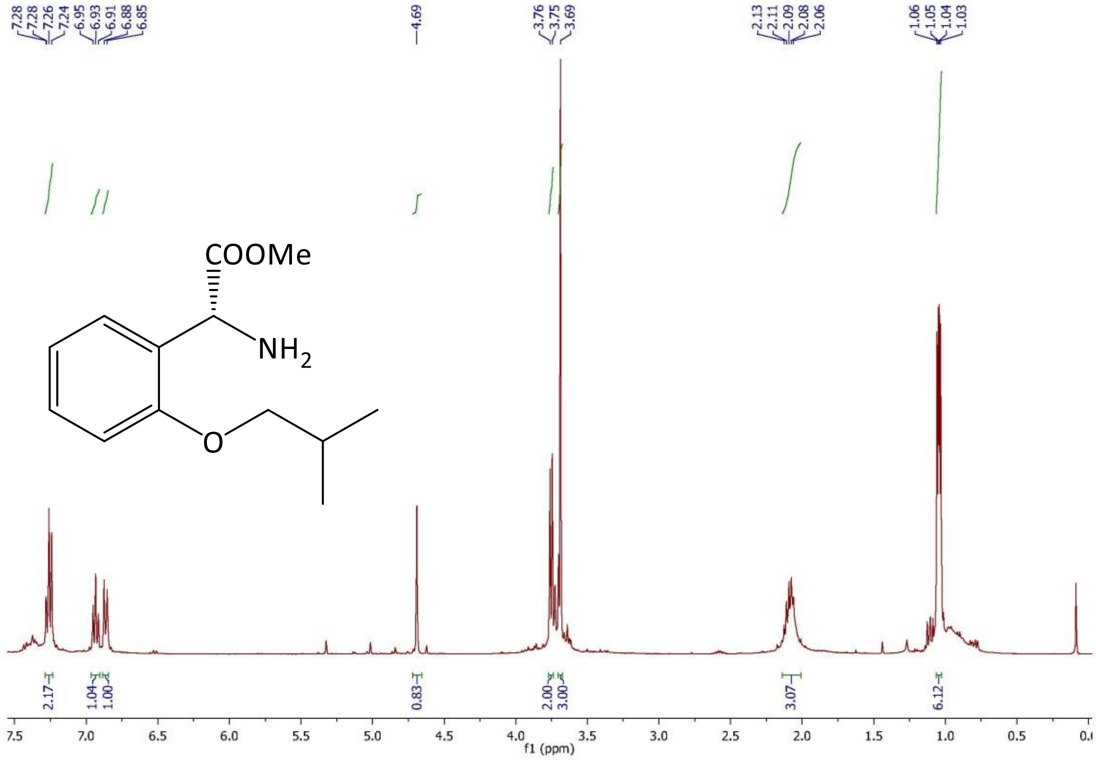

^{1}H NMR ($CDCl_3$, 400.13 MHz, 298K) of 5ad

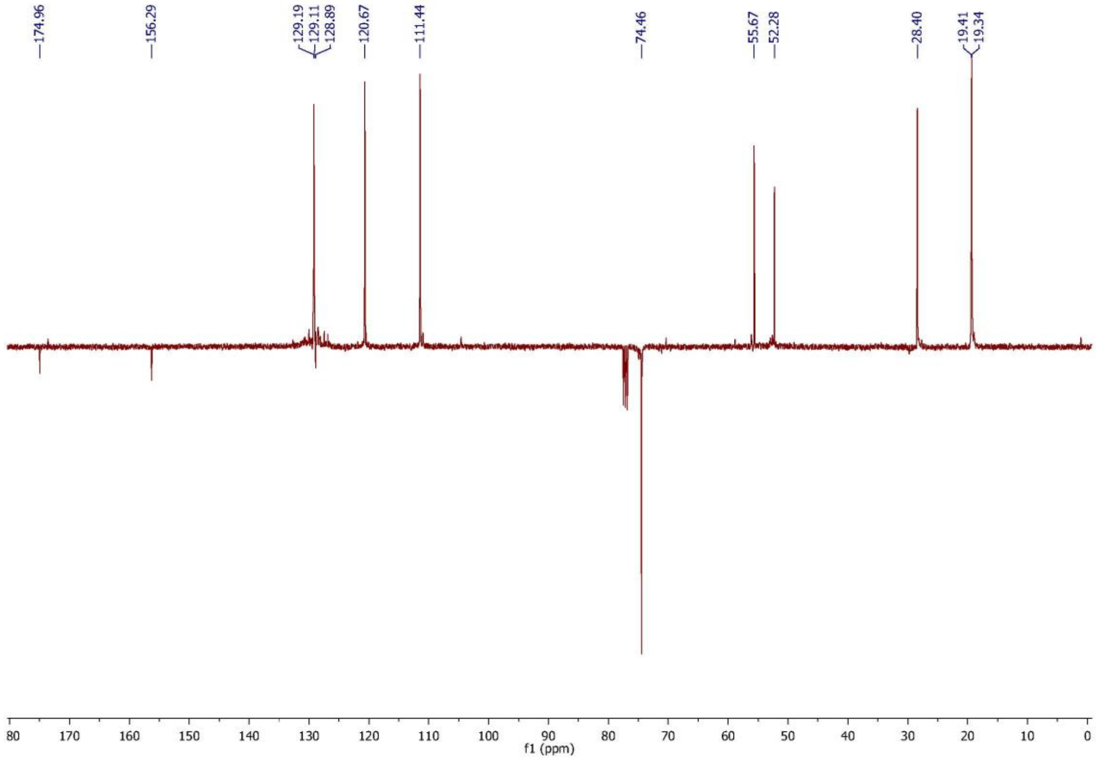

$^{13}C\{^{1}H\}$ NMR (APT, $CDCl_3$, 100.61 MHz, 298K) of 5ad

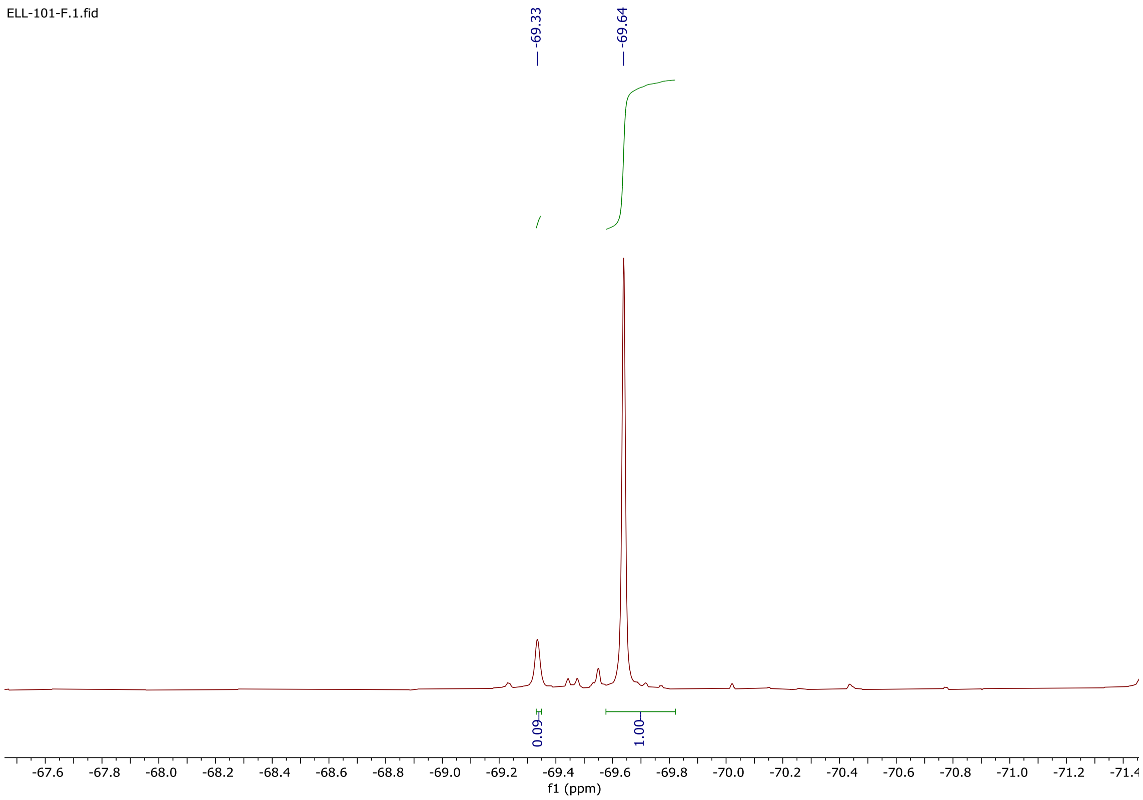

^{19}F NMR ($CDCl_3$, 282.40 MHz, 298K) of 5ad

(*R*)-Methyl 2’-(3-methyl-1-butoxy)phenylglycinate (5ae)

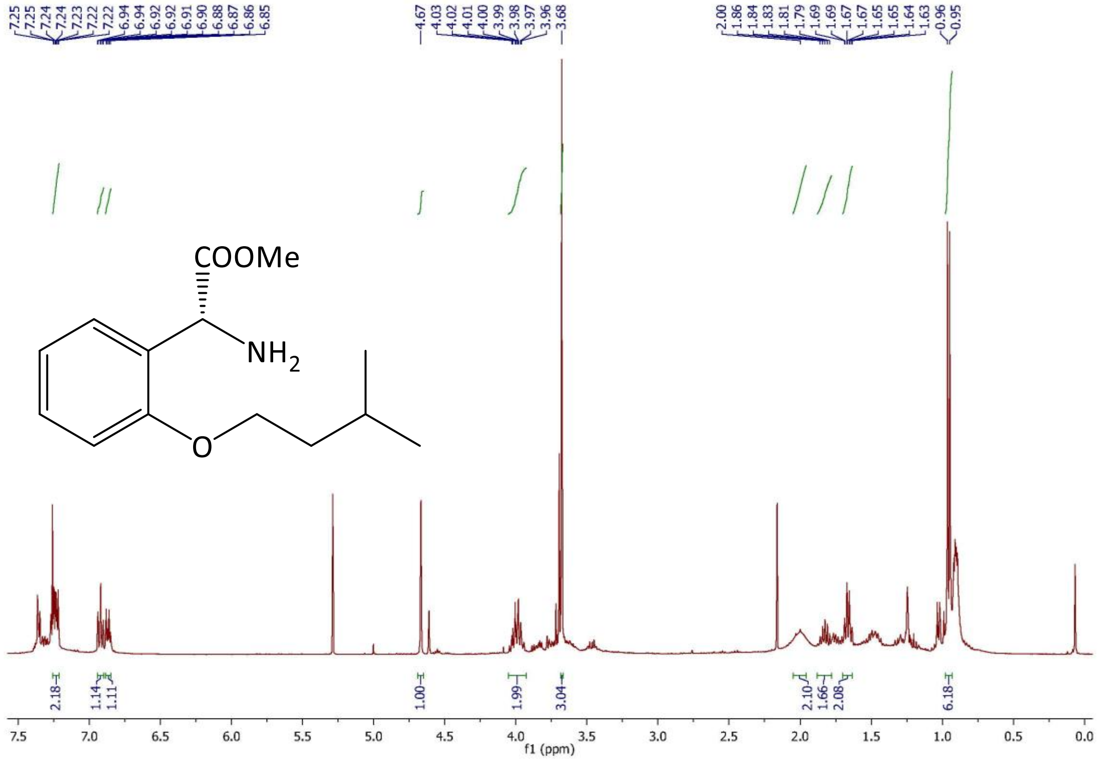

^{1}H NMR ($CDCl_3$, 400.13 MHz, 298K) of 5ae

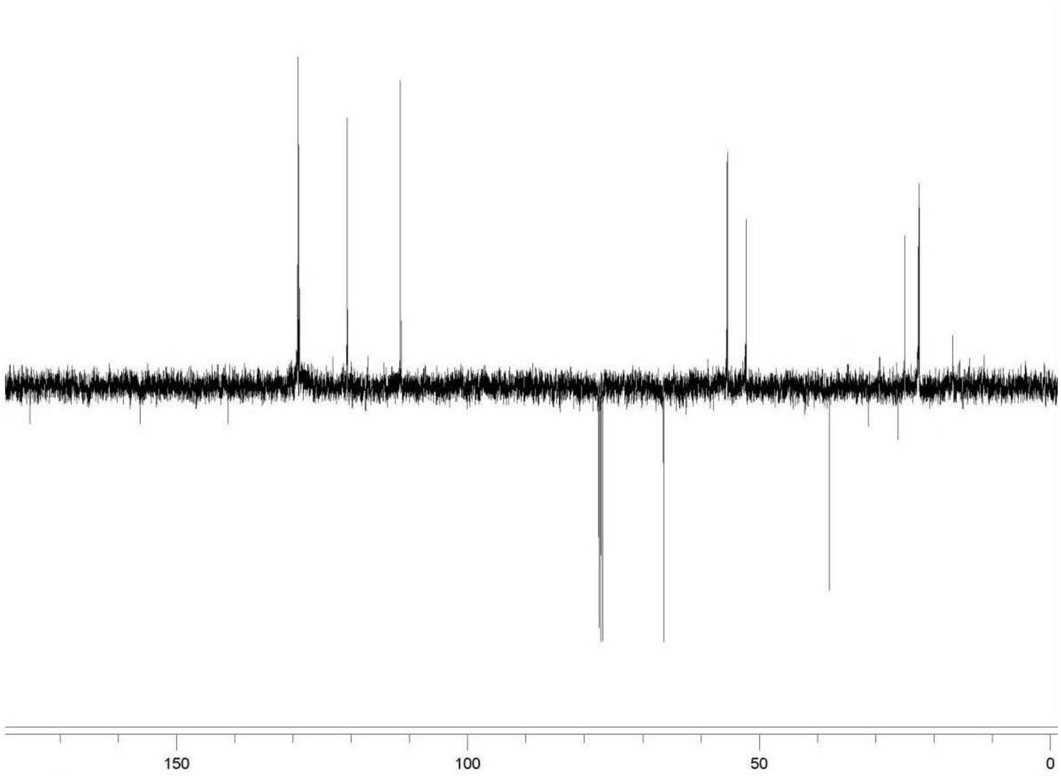

$^{13}C\{^{1}H\}$ NMR (APT, $CDCl_3$, 100.61 MHz, 298K) of 5ae

(*R*)-Methyl 2’-(benzyloxy)phenylglycinate (5af)

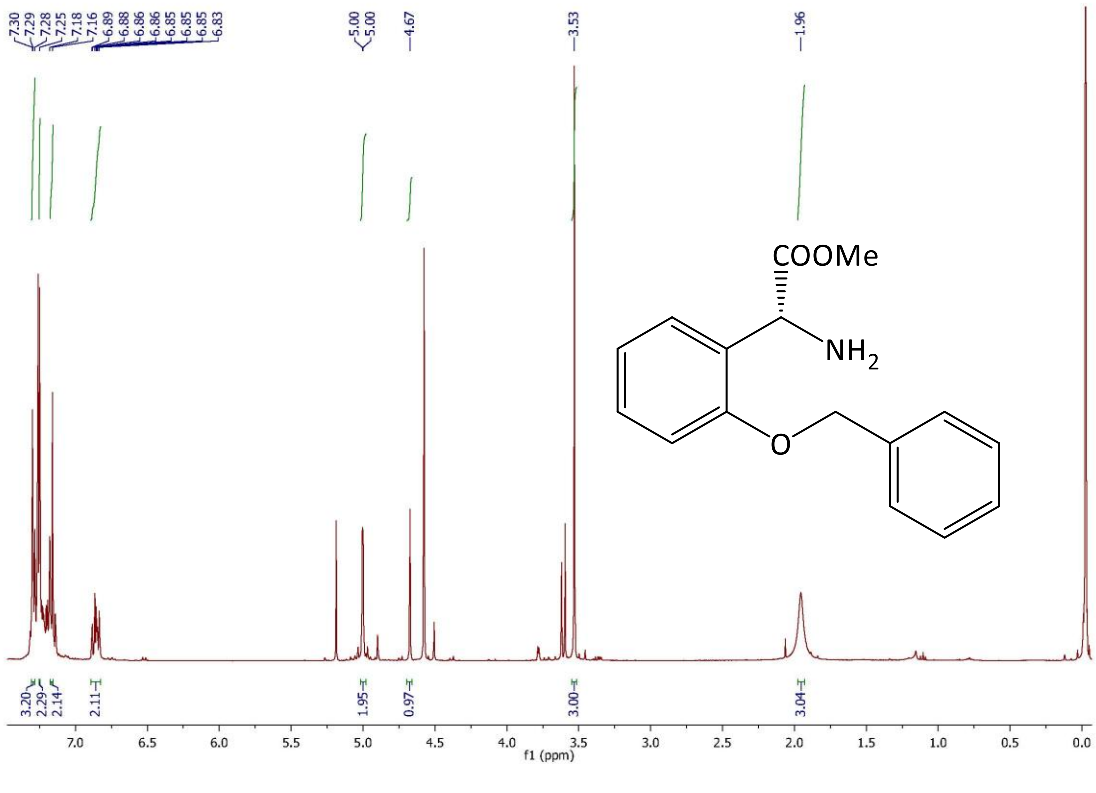

^{1}H NMR ($CDCl_3$, 400.13 MHz, 298K) of 5af

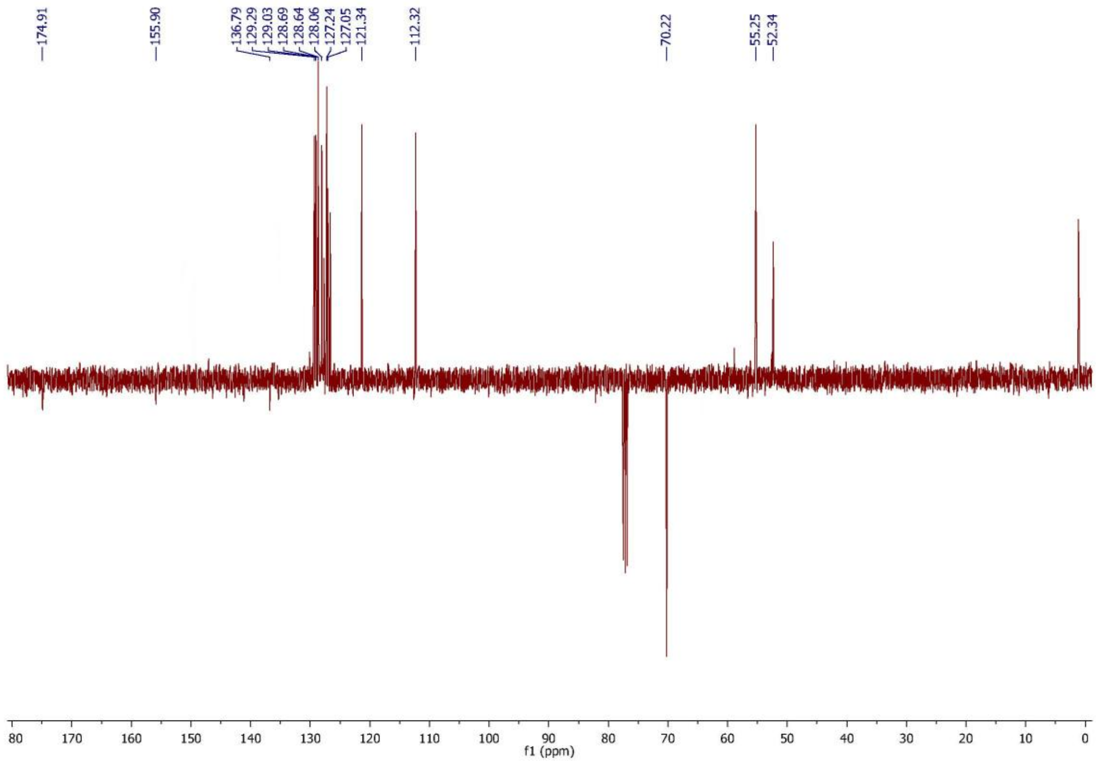

$^{13}C\{^{1}H\}$ **NMR (APT, $CDCl_3$, 100.61 MHz, 298K) of 5af**

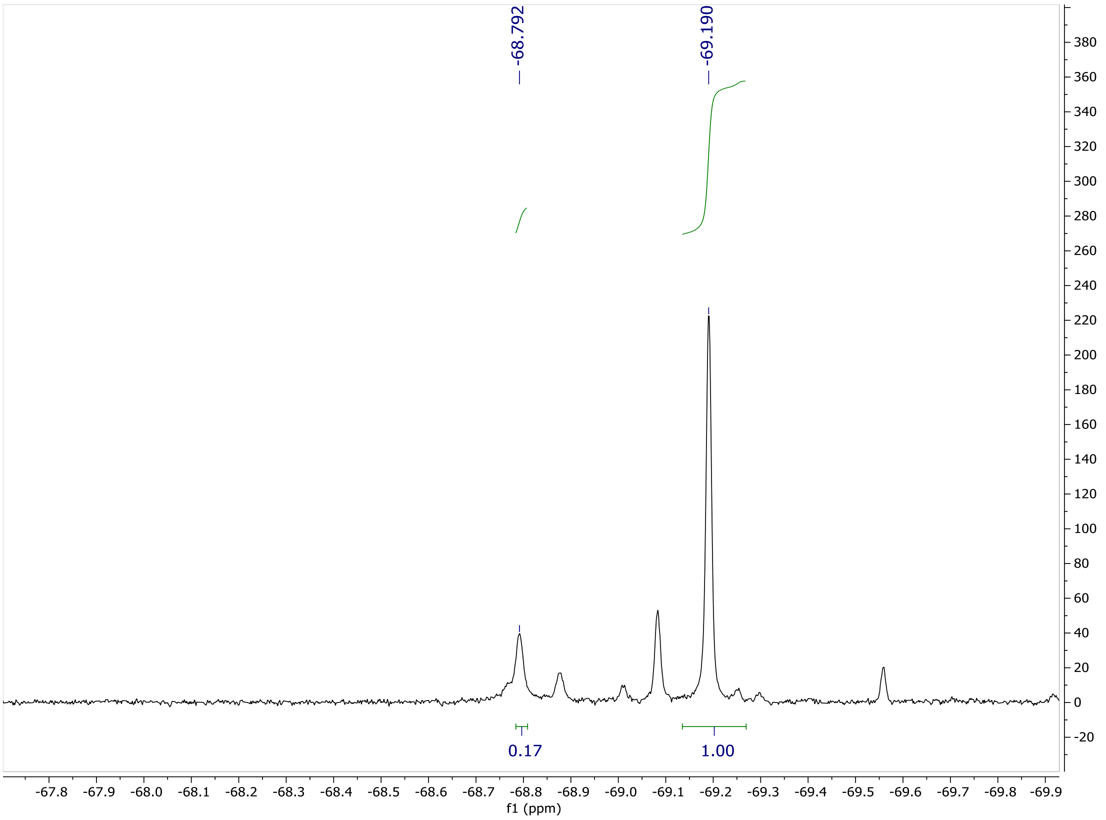

^{19}F **NMR ($CDCl_3$, 282.40 MHz, 298K) of 5af**

(*R*)-Methyl 2’-(3,4-dimethoxybenzyloxy)phenylglycinate (5ag)

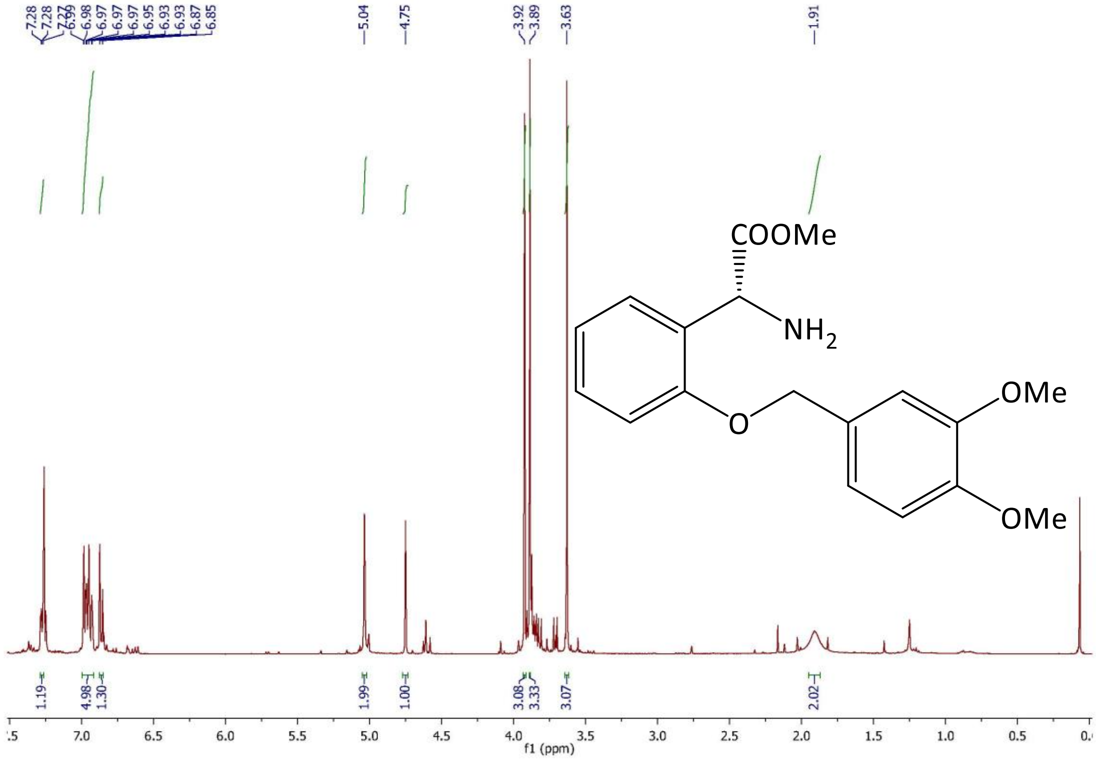

^{1}H NMR (CDCl$_3$, 400.13 MHz, 298K) of 5ag

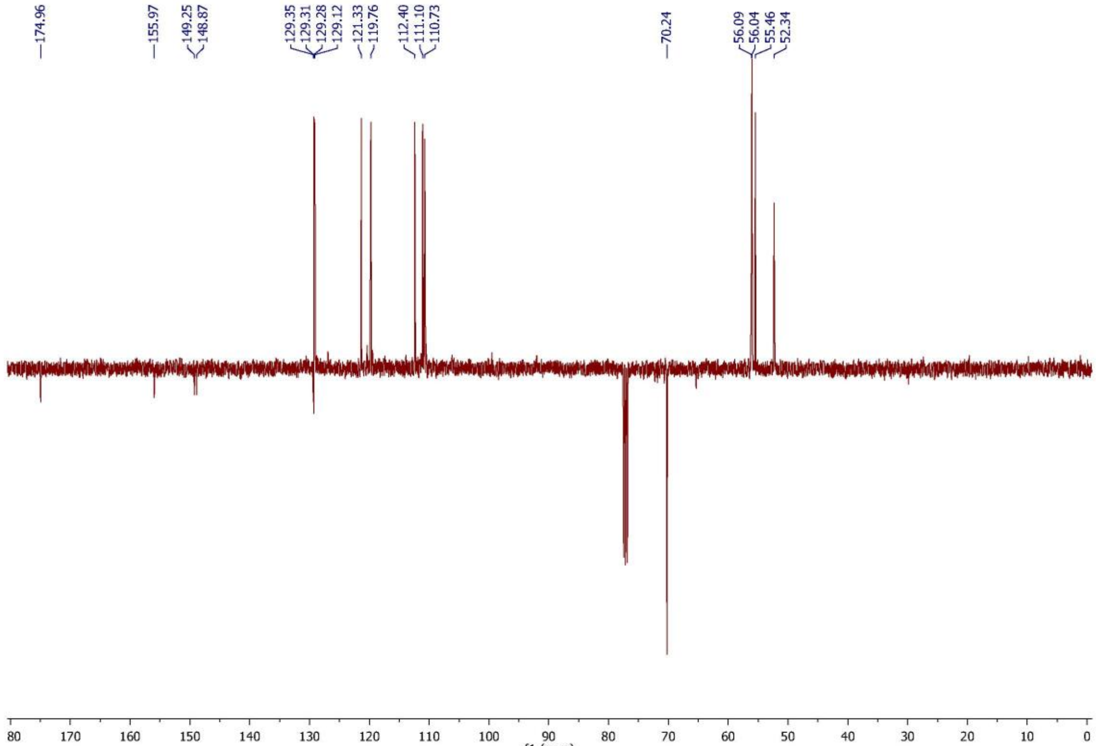

^{13}C{^{1}H} NMR (APT, CDCl$_3$, 100.61 MHz, 298K) of 5ag

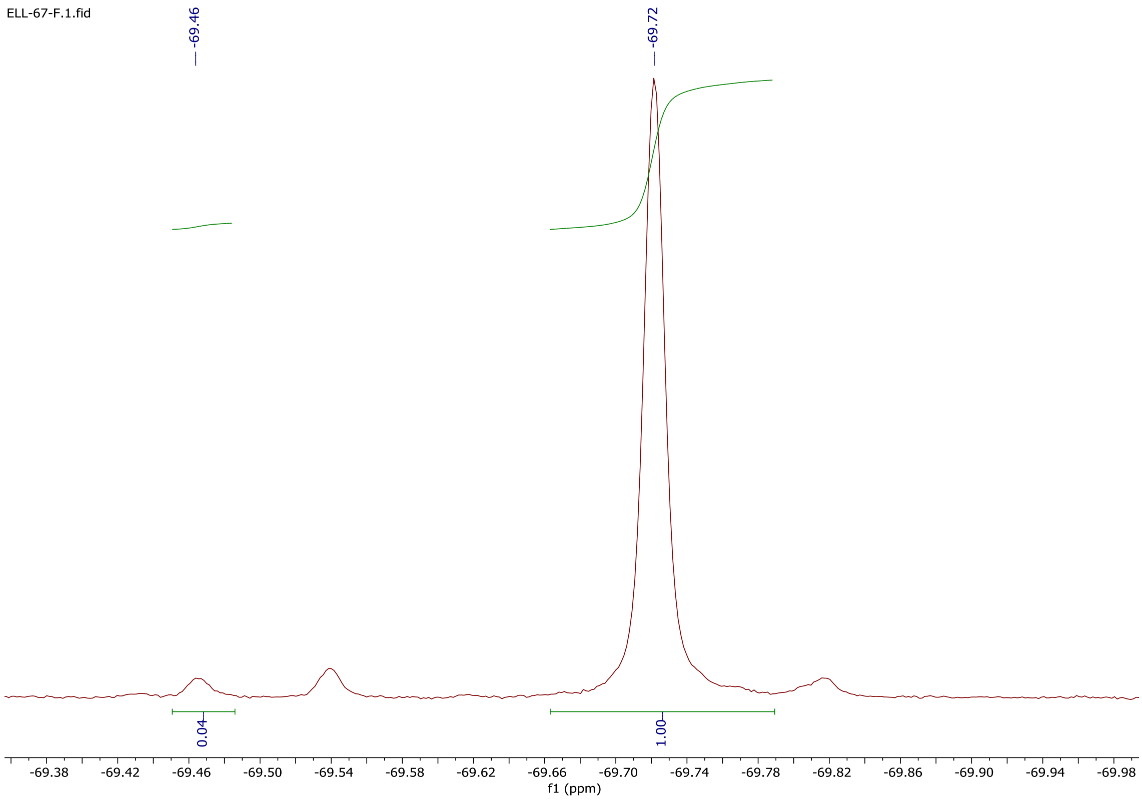

^{19}F NMR (CDCl$_3$, 282.40 MHz, 298K) of 5ag

(*R*)-Methyl 2'-(cyclopentyloxy)phenylglycinate (5ah)

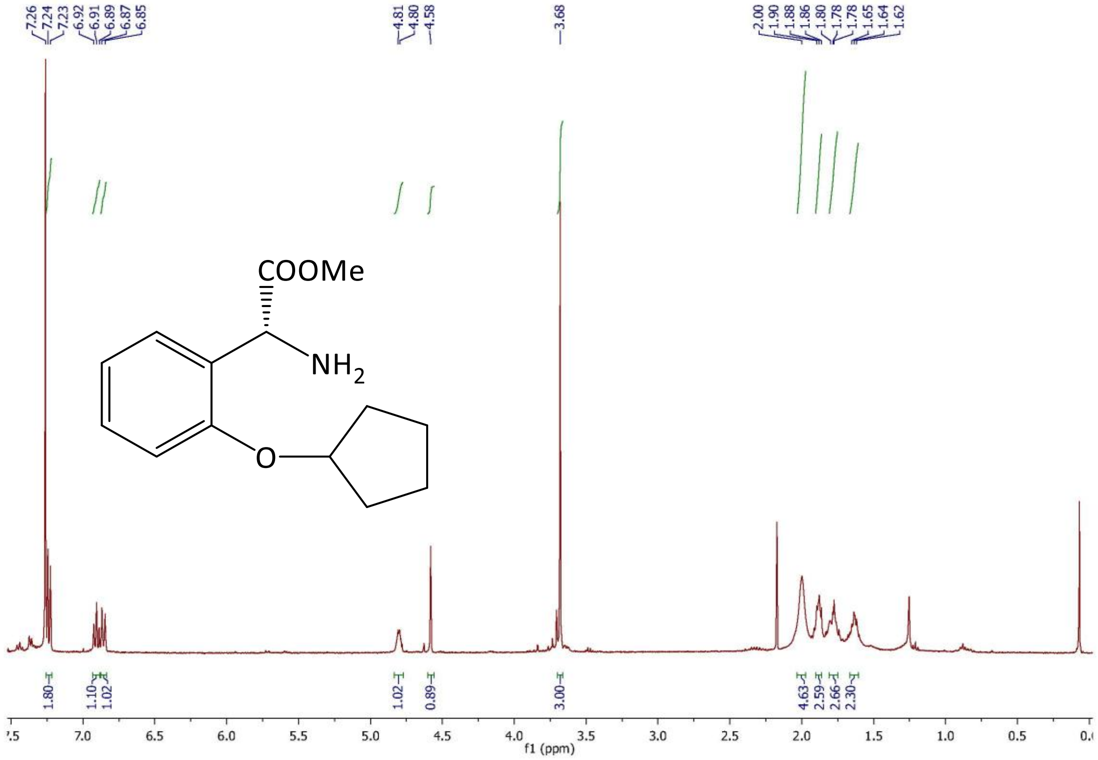

^{1}H NMR (CDCl$_3$, 400.13 MHz, 298K) of 5ah

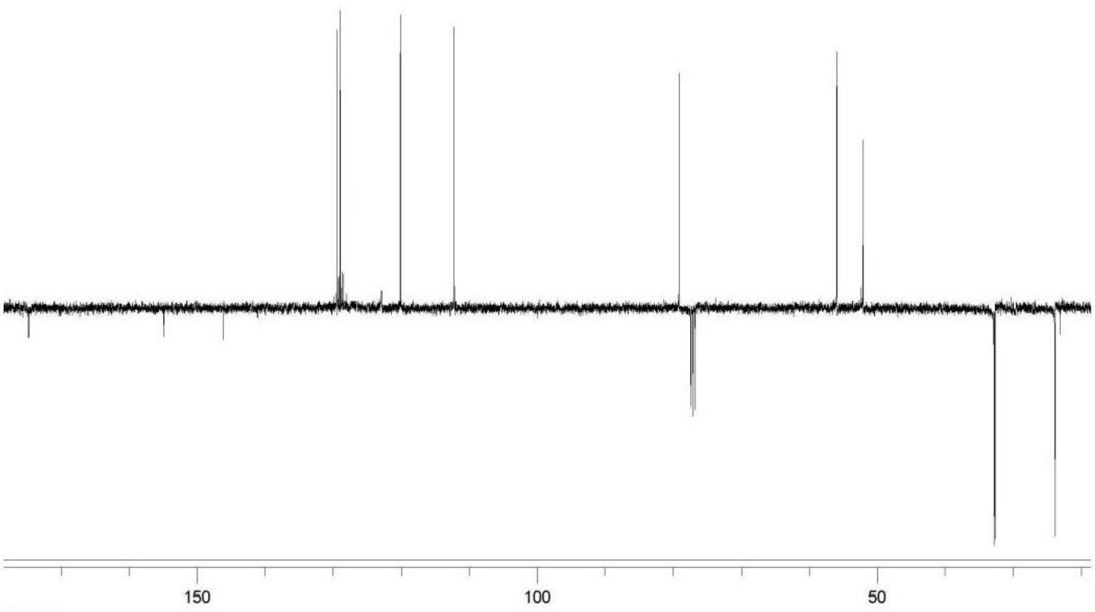

$^{13}C\{^1H\}$ NMR (APT, $CDCl_3$, 100.61 MHz, 298K) of 5ah

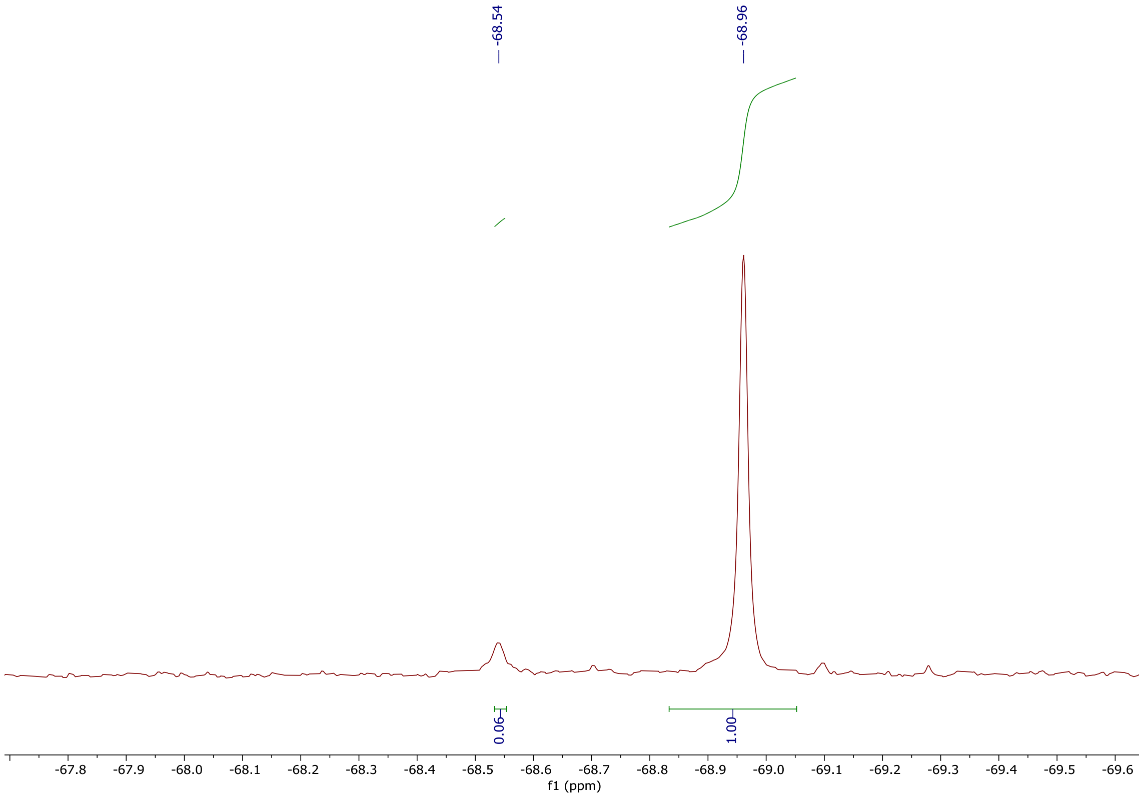

^{19}F NMR ($CDCl_3$, 282.40 MHz, 298K) of 5ah

(*R*)-Methyl 2'-(cyclohexyloxy)phenylglycinate (5ai)

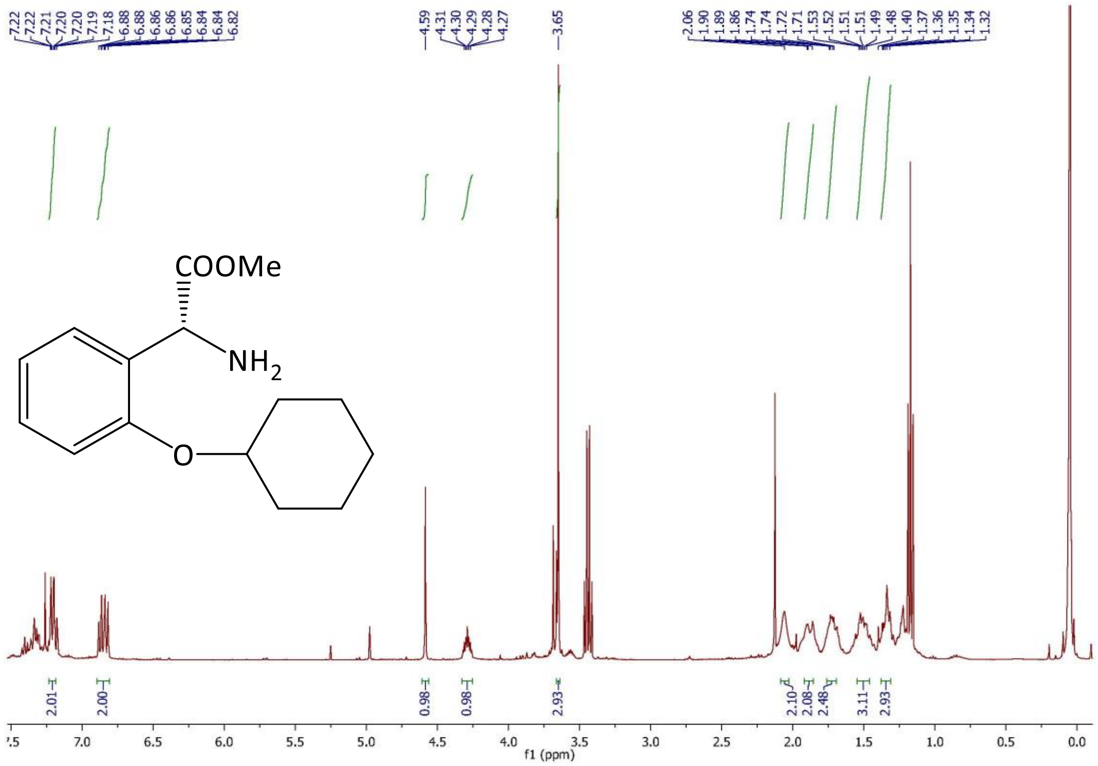

^{1}H NMR (CDCl$_3$, 400.13 MHz, 298K) of 5ai

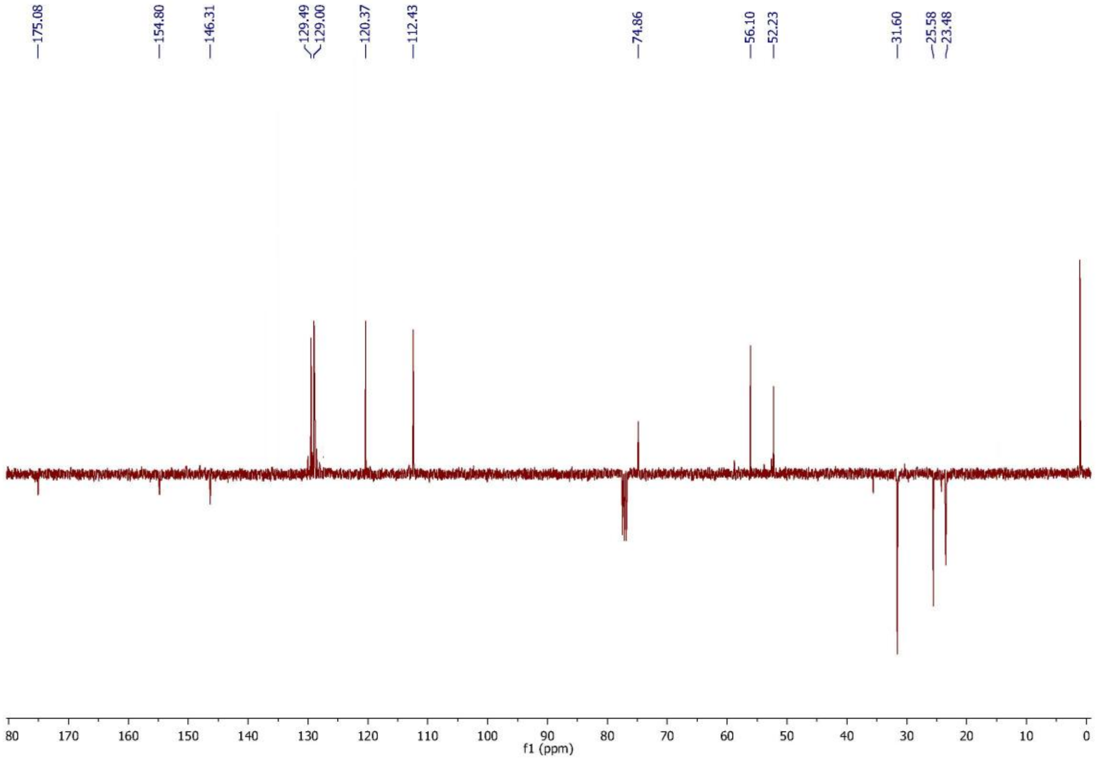

^{13}C{^{1}H} NMR (APT, CDCl$_3$, 100.61 MHz, 298K) of 5ai

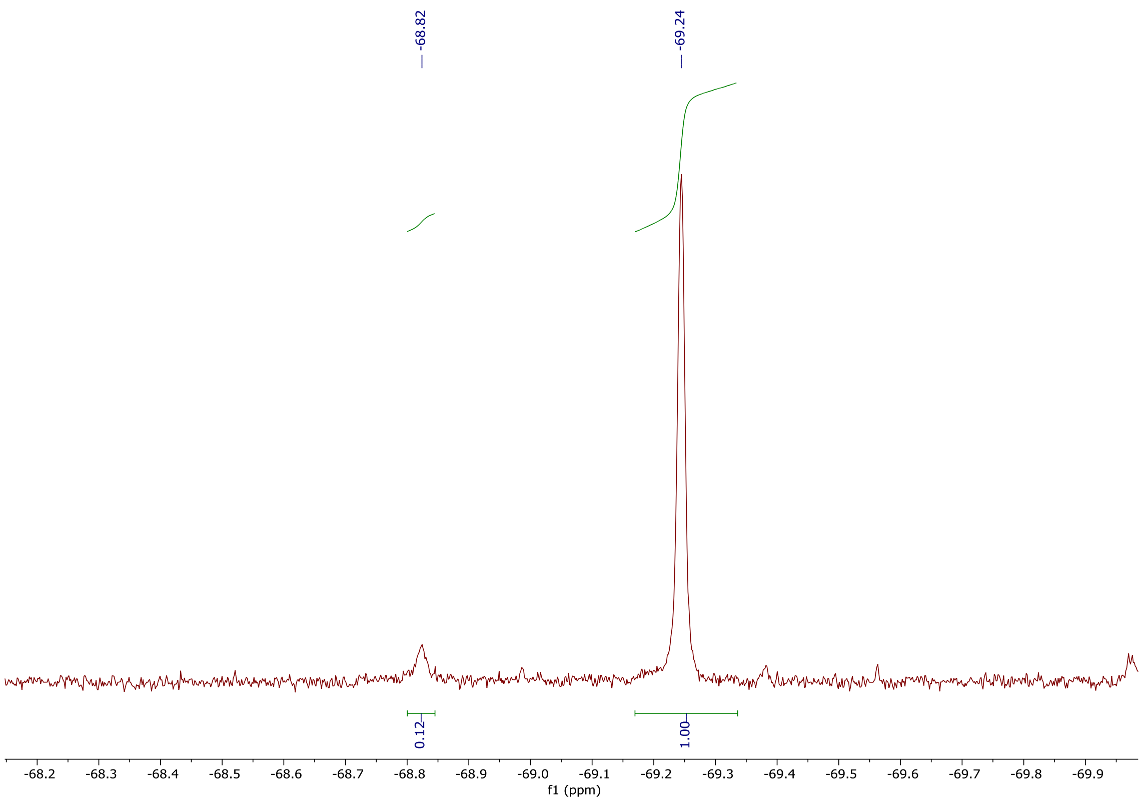

^{19}F NMR ($CDCl_3$, 282.40 MHz, 298K) of 5ai

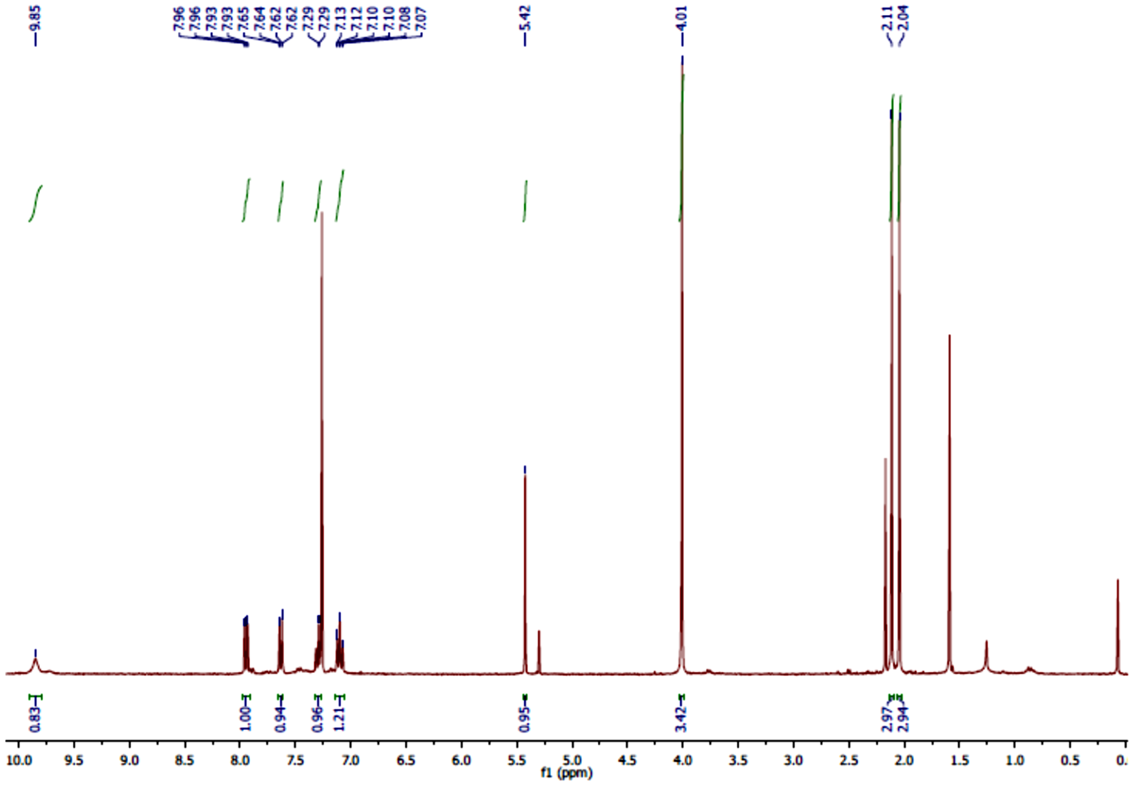

^{1}H NMR ($CDCl_3$, 300.13 MHz, 298K) of 7

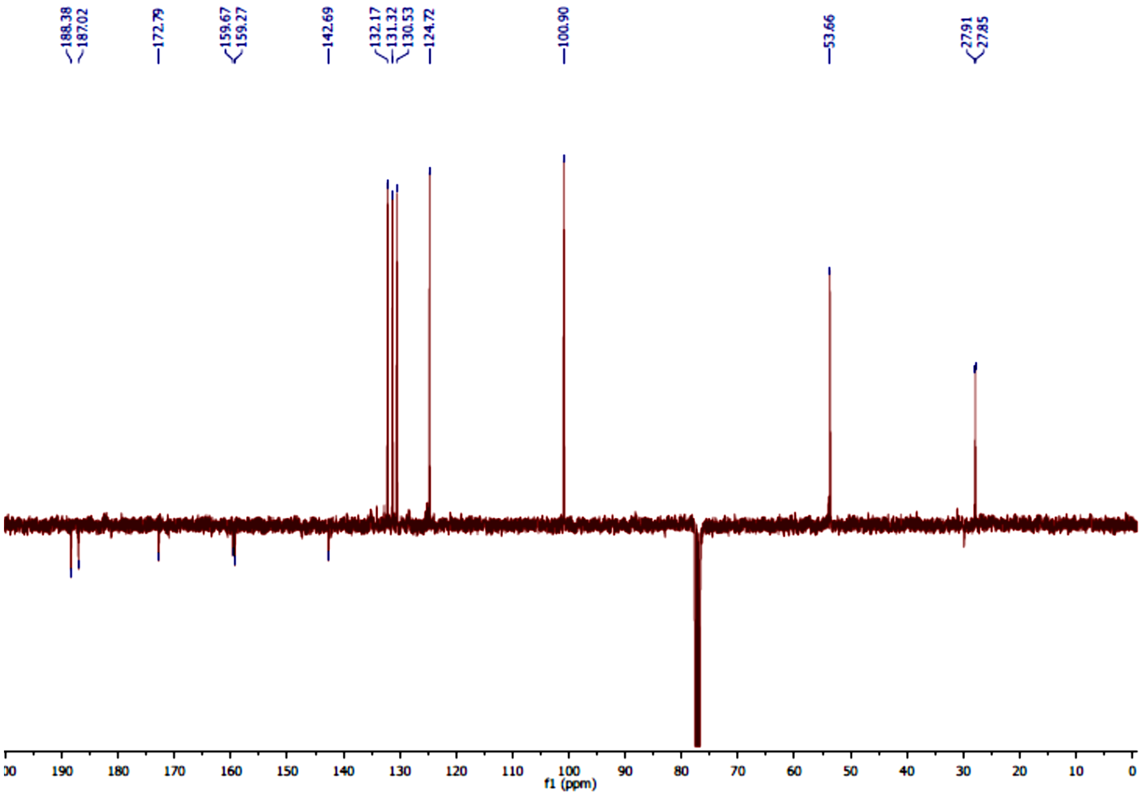

$^{13}C\{^{1}H\}$ NMR (APT, $CDCl_3$, 75.47 MHz, 298K) of 7

X-ray crystal structure of 6

Table S2: Crystallographic data: collection, solution and refinement

Empirical formula	$C_{14}H_{15}ClNO_7Pd_2$	
Formula weight	557.52	
Temperature	100 K	
Wavelength	Mo Kα radiation, λ = 0.71073 Å	
Crystal system	Orthorhombic	
Space group	Pnma	
Unit cell dimension	a = 9.8915(10) Å	α = 90°
	b = 21.855(2) Å	β = 90°
	c = 16.4995(16) Å	γ = 90°
Volume	3566.9(6) $Å^3$	
Z	8	
Density(calculated)	2.076 mg/m^3	
Absorption coefficient	2.20 mm^{-1}	
F(000)	2168	
Crystal size	0.13 × 0.07 × 0.06 mm	
Theta (θ) range for data collection	1.6 to 19.7°	
Index ranges	-9≤h≤9, -20≤k≤20, -15≤l≤15	
Reflections collected	14871	
Independent reflections	1649	
Completeness to theta = 19.7°	99.9 %	
Max. and min. transmission	Tmax = 0.889, Tmin = 0.769	
Refinement method	Full-matrix least-squares on F^2	
Data / restraints / parameters	1649 / 10 / 237	
Goodness-of-fit on F^2	1.05	
Final R indices [I>2sigma(I)]	R_1 = 0.035, wR_2 = 0.092	
R indices(all data)	R_1 = 0.048, wR_2 = 0.099	
Largest diff. peak and hole	0.74 and −0.60 e.$Å^{-3}$	

Table S3: Selected bond distances and angles for compound 6

Bond distances (Å):

Pd(1)—N	1.945(8)	O(6)—C(8)	1.177(11)
Pd(1)—O(3)	2.013(6)	O(7)—C(15)	1.164(13)
Pd(1)—O(1)	2.016(6)	O(7)—C(16)	1.287(17)
Pd(1)—Cl(2)	2.317(2)	O(7')—C(11)	1.110(9)
Pd(1)—Pd(2)	3.0951(10)	O(7')—C(16')	1.36(3)
Pd(1)—Pd(1')	3.1945(15)	N—C(7)	1.273(11)
Pd(2)—N	1.967(7)	C(1)—C(2)	1.509(12)
Pd(2)—O(2)	2.042(6)	C(3)—O(4')	1.261(8)
Pd(2)—O(4)	2.048(6)	C(3)—C(4)	1.503(17)
Pd(2)—Cl(1)	2.344(2)	C(5)—O(3')	1.274(8)
Pd(2)—Pd(2')	3.1546(15)	C(5)—C(6)	1.432(18)
Cl(1)—Pd(2')	2.344(2)	C(7)—C(10)	1.481(12)
Cl(2)—Pd(1')	2.317(2)	C(7)—C(8)	1.522(14)
O(1)—C(1)	1.261(12)	C(10)—C(11)	1.3900
O(2)—C(1)	1.271(11)	C(10)—C(15)	1.3900
O(3)—C(5)	1.274(8)	C(11)—C(12)	1.3900
O(4)—C(3)	1.261(8)	C(12)—C(13)	1.3900
O(5)—C(8)	1.308(12)	C(13)—C(14)	1.3900
O(5)—C(9)	1.451(11)	C(14)—C(15)	1.3900

Bond angles (°):

N—Pd(1)—O(3)	175.1(3)	C(3)—O(4)—Pd(2)	128.8(7)
N—Pd(1)—O(1)	91.8(3)	C(8)—O(5)—C(9)	115.8(8)
O(3)—Pd(1)—O(1)	83.4(2)	C(15)—O(7)—C(16)	116.2(13)
N—Pd(1)—Cl(2)	92.6(2)	C(11)—O(7')—C(16')	116(2)
O(3)—Pd(1)—Cl(2)	92.14(19)	C(7)—N—Pd(1)	132.6(6)
O(1)—Pd(1)—Cl(2)	175.53(19)	C(7)—N—Pd(2)	122.7(6)
N—Pd(1)—Pd(2)	38.0(2)	Pd(1)—N—Pd(2)	104.6(4)
O(3)—Pd(1)—Pd(2)	140.95(17)	O(1)—C(1)—O(2)	128.4(9)
O(1)—Pd(1)—Pd(2)	79.34(17)	O(1)—C(1)—C(2)	116.2(9)
Cl(2)—Pd(1)—Pd(2)	104.58(8)	O(2)—C(1)—C(2)	115.2(10)
N—Pd(1)—Pd(1')	107.4(2)	O(4)—C(3)—O(4')	127.5(12)
O(3)—Pd(1)—Pd(1')	76.66(18)	O(4)—C(3)—C(4)	116.2(6)
O(1)—Pd(1)—Pd(1')	132.34(17)	O(4')—C(3)—C(4)	116.2(6)

Cl(2)—Pd(1)—Pd(1’)	46.41(6)	O(3)—C(5)—O(3’)	125.6(13)
Pd(2)—Pd(1)—Pd(1’)	89.631(19)	O(3)—C(5)—C(6)	117.2(6)
N—Pd(2)—O(2)	91.4(3)	O(3’)—C(5)—C(6)	117.2(6)
N—Pd(2)—O(4)	174.8(3)	N—C(7)—C(10)	121.0(8)
O(2)—Pd(2)—O(4)	84.9(2)	N—C(7)—C(8)	123.3(9)
N—Pd(2)—Cl(1)	91.9(2)	C(10)—C(7)—C(8)	115.7(9)
O(2)—Pd(2)—Cl(1)	176.51(17)	O(6)—C(8)—O(5)	125.1(9)
O(4)—Pd(2)—Cl(1)	91.84(18)	O(6)—C(8)—C(7)	122.1(10)
N—Pd(2)—Pd(1)	37.5(2)	O(5)—C(8)—C(7)	112.9(10)
O(2)—Pd(2)—Pd(1)	77.46(17)	C(11)—C(10)—C(15)	120.0
O(4)—Pd(2)—Pd(1)	144.20(17)	C(11)—C(10)—C(7)	118.9(9)
Cl(1)—Pd(2)—Pd(1)	104.62(8)	C(15)—C(10)—C(7)	121.0(9)
N—Pd(2)—Pd(2’)	107.8(2)	O(7’)—C(11)—C(12)	115.3(15)
O(2)—Pd(2)—Pd(2’)	129.98(16)	O(7’)—C(11)—C(10)	124.5(15)
O(4)—Pd(2)—Pd(2’)	77.41(17)	C(12)—C(11)—C(10)	120.0
Cl(1)—Pd(2)—Pd(2’)	47.70(5)	C(11)—C(12)—C(13)	120.0
Pd(1)—Pd(2)—Pd(2’)	90.369(19)	C(12)—C(13)—C(14)	120.0
Pd(2)—Cl(1)—Pd(2’)	84.59(10)	C(15)—C(14)—C(13)	120.0
Pd(1)—Cl(2)—Pd(1’)	87.17(11)	O(7)—C(15)—C(14)	120.5(12)
C(1)—O(1)—Pd(1)	126.8(6)	O(7)—C(15)—C(10)	119.5(12)
C(1)—O(2)—Pd(2)	127.9(6)	C(14)—C(15)—C(10)	120.0
C(5)—O(3)—Pd(1)	130.5(7)		

Symmetry code: (i) x,-y+3/2,-z

X-ray crystal structure of 7

Table S4: Crystallographic data: collection, solution and refinement for 7

Empirical formula	$C_{14}H_{15}NO_4Pd$	
Formula weight	367.67	
Temperature	173 K	
Wavelength	Mo Kα radiation, λ = 0.71073 Å	
Crystal system	Triclinic	
Space group	P-1	
Unit cell dimension	a = 7.4456(7) Å	α = 68.132(2)°
	b = 8.6432(9) Å	β = 84.629(2)°
	c = 11.4793(12) Å	γ = 84.610(2)°
Volume	681.17(12) $Å^3$	
Z	2	
Density(calculated)	1.793 mg/m3	
Absorption coefficient	1.37 mm-1	
F(000)	368	
Crystal size	0.30 × 0.23 × 0.22mm	
Theta (θ) range for data collection	1.9 to 30.0°	
Index ranges	-10≤h≤10, -12≤k≤12, -16≤l≤15	
Reflections collected	8530	
Independent reflections	3945	
Completeness to theta = 30.0°	99.1 %	
Max. and min. transmission	Tmax = 0.739, Tmin = 0.679	
Refinement method	Full-matrix least-squares on F2	
Data / restraints / parameters	3945 / 0 / 187	
Goodness-of-fit on F2	1.06	
Final R indices [I>2sigma(I)]	R1 = 0.021, wR2 = 0.053	
R indices(all data)	R1 = 0.023, wR2 = 0.054	
Largest diff. peak and hole	0.41 and −0.74 e.Å-3	

Table S5: Selected bond distances and angles for compound 7

Bond distances (Å):

Pd(1)—C(7)	1.9598(16)	C(5)—C(6)	1.389(3)
Pd(1)—N(1)	1.9832(14)	C(6)—C(7)	1.392(2)
Pd(1)—O(4)	2.0048(12)	C(8)—O(1)	1.204(2)
Pd(1)—O(3)	2.0806(13)	C(8)—O(2)	1.316(2)
N(1)—C(1)	1.287(2)	O(2)—C(9)	1.448(2)
C(1)—C(2)	1.461(2)	O(3)—C(13)	1.269(2)
C(1)—C(8)	1.513(2)	C(10)—O(4)	1.279(2)
C(2)—C(3)	1.395(2)	C(10)—C(12)	1.390(3)
C(2)—C(7)	1.419(2)	C(10)—C(11)	1.505(2)
C(3)—C(4)	1.387(2)	C(12)—C(13)	1.404(2)
C(4)—C(5)	1.383(3)	C(13)—C(14)	1.504(3)

Bond angles (°):

C(7)—Pd(1)—N(1)	80.29(6)	C(6)—C(7)—C(2)	118.25(15)
C(7)—Pd(1)—O(4)	92.18(6)	C(6)—C(7)—Pd(1)	126.75(12)
N(1)—Pd(1)—O(4)	172.38(5)	C(2)—C(7)—Pd(1)	114.99(12)
C(7)—Pd(1)—O(3)	174.69(6)	O(1)—C(8)—O(2)	125.42(15)
N(1)—Pd(1)—O(3)	94.52(5)	O(1)—C(8)—C(1)	121.33(15)
O(4)—Pd(1)—O(3)	93.04(5)	O(2)—C(8)—C(1)	113.25(14)
C(1)—N(1)—Pd(1)	117.61(12)	C(8)—O(2)—C(9)	115.67(14)
N(1)—C(1)—C(2)	115.28(14)	C(13)—O(3)—Pd(1)	122.53(12)
N(1)—C(1)—C(8)	116.24(14)	O(4)—C(10)—C(12)	127.35(16)
C(2)—C(1)—C(8)	128.47(14)	O(4)—C(10)—C(11)	114.17(16)
C(3)—C(2)—C(7)	121.00(15)	C(12)—C(10)—C(11)	118.48(16)
C(3)—C(2)—C(1)	127.17(15)	C(10)—C(12)—C(13)	127.27(16)
C(7)—C(2)—C(1)	111.81(14)	O(3)—C(13)—C(12)	126.16(16)
C(4)—C(3)—C(2)	119.17(16)	O(3)—C(13)—C(14)	115.51(16)
C(5)—C(4)—C(3)	120.32(16)	C(12)—C(13)—C(14)	118.32(15)
C(4)—C(5)—C(6)	120.89(16)	C(10)—O(4)—Pd(1)	123.50(12)
C(5)—C(6)—C(7)	120.35(16)		
